# Diverse Structures of Tea Polyphenols from Rougui Wuyi Rock Tea and Their Potential as Inhibitor of 3C-like Protease

**DOI:** 10.3390/molecules30051024

**Published:** 2025-02-23

**Authors:** Qing Sun, Xiaojuan Chen, Jie Zhang, Juan Song, Lan Yao, Yang Zhao, Guang Yang, Xiu Wang, Haizhen Liang, Baiping Ma

**Affiliations:** Beijing Institute of Radiation Medicine, Beijing 100850, China; sunqing20200808@foxmail.com (Q.S.); chenxj626@163.com (X.C.); zhangjie061003@163.com (J.Z.); fltcsong@163.com (J.S.); ycl_0510@163.com (L.Y.); young_106@163.com (Y.Z.); wangxiu0627@foxmail.com (X.W.)

**Keywords:** tea polyphenols, Rougui Wuyi Rock tea, antiviral, 3CLpro

## Abstract

Tea polyphenols, the primary bioactive constituents responsible for the various health benefits of tea, can be categorized into different subgroups according to their structural characteristics. However, the distinctions in antiviral activity among the diverse types of polyphenolic compounds remain unexplored. In the present study, fifty-eight tea polyphenols with varied structures, including eleven undescribed compounds, were isolated from Rougui Wuyi rock tea. Their molecular structures were elucidated using comprehensive analytical approaches of NMR, HRMS, CD spectroscopic data and acid hydrolysis. The isolated polyphenol analogs could be structurally classified into two main categories: flavan-3-ols, which include catechins, flavoalkaloids, procyanidins and theasinensins, and flavones, encompassing kaempferol, quercetin, myricetin, and their respective glycosides. The inhibitory activities of fifty-eight tea polyphenols against 3CLpro were assessed in vitro, and eighteen phenolic compounds exhibited inhibitory effects on 3CLpro, with IC_50_ values ranging from 9.8 μM to 61.1 μM. Among them, two types of tea polyphenols, catechin and flavoalkaloid derivatives, demonstrated superior inhibitory effects compared to other categories. The structure-activity relationship was further explored, and molecular docking analysis revealed that the differing inhibitory effects of catechin and flavoalkaloid derivatives were attributed to the variations in the number and positions of the hydrogen bond interactions with 3CLpro. This study provides a valuable understanding of tea polyphenols and supplies potential lead compounds for antiviral drugs.

## 1. Introduction

Tea, a time-honored drink with a documented history spanning millennia in China, has become the most consumed beverage around the world. Commercial tea, manufactured from the fresh tender leaves of *Camellia sinensis*, is categorized into various types based on the varying degree of fermentation, such as green tea (non-fermented), oolong tea (semi-fermented), and black tea (fermented) [1]. Beyond its role as a relaxation drink, the market demand for tea has steadily increased due to its extensive health benefits supported by plenty of scientific research, such as antioxidant [2], antidiabetic [3], antiviral [4], antimicrobial [5] and so on. Particularly, recent studies have highlighted the potent antiviral activity of tea against various viruses, including influenza virus [6], herpes simplex virus [7], human immunodeficiency virus (HIV) [8], and even the rapid spread of a novel coronavirus called SARS-CoV-2 in the past few years [9,10,11].

Tea polyphenols are the major bioactive constituents responsible for the unique flavor and diverse health-promoting properties [12]. The chemical structures of polyphenols are characterized by the multiple hydroxyl groups attached to different positions. Nowadays, various tea polyphenols have been identified and could be classified into different subgroups based on the structural characteristics. Catechins are the most critical constituents in tea, with (−)-epigallocatechin-3-gallate (EGCG) usually being the most abundant [13]. For example, catechins account for 60–80% of all polyphenols present in green tea, with EGCG being 30–40% of all catechins [14]. Procyanidin oligomers (B type) are mainly composed of (epi)catechin and (epi)gallocatechin. The substitution patterns, characterized by C4→C8 or C4→C6 linkages, vary depending on the C-2 and C-3 configurations of their constituent units [15]. Theasinensins, primarily found in semi- and fully fermented tea, are catechin dimers formed through the oxidative coupling of C-C bonds on the B-ring [16,17]. Theaflavins, a class of polyphenol produced during fermentation, are characterized by the benzotropolone skeleton [18], which mainly exists in fermented tea, such as black tea. Flavoalkaloids represent a unique class of compounds in tea, featuring an *N*-ethyl-2-pyrrolidinone moiety substituted at the A ring of a flavan-3-ol skeleton [19]. While the aforementioned subgroups of tea polyphenols all share a C6-C3-C6 flavan-3-ol skeleton, flavones, another large group of phenolic derivatives, could also be found in tea, including quercetin, myricetin, and kaempferol, along with their respective glycosides [1]. Notably, teaghrelins are unique acylated flavonoid tetraglycosides found in oolong tea, which have been shown to possess effects similar to those of the endogenous hunger hormone ghrelin [20]. Despite the fact that tea polyphenols have been explored extensively, there are still many unknown polyphenolic compounds in tea that have not been discovered [21,22].

Currently, significant progress has been made in exploring the bioactive constituents in tea that contribute to its antiviral properties. Partial tea polyphenols, including EGCG, theaflavin, and kaempferol, were shown to exert potential anti-SARS-CoV-2 effects via various pathways [23,24,25]. 3CLpro, a dimeric protease consisting of two identical protomers, could cleave the non-structural proteins at 11 different sites, thereby interrupting the viral replication cycle, making 3CLpro a primary target for the development of antiviral drugs [26,27]. Extensive molecular docking studies have demonstrated that EGCG and theaflavin derivatives, such as theaflavin (TF) and theaflavin-3,3′-di-gallate (TFDG), exhibited positive binding affinities towards 3CLpro [28,29,30]. In vitro experiments further confirmed the inhibitory effects of EGCG and TF on SARS-CoV-2 3CLpro protein activity, with IC_50_ values being 16.6 μM for EGCG and 15.0 μM for theaflavin, respectively [31]. Although there is increasing evidence suggesting that polyphenolic compounds are linked to the antiviral activity of tea, most studies have only focused on some compounds, such as EGCG, theaflavin derivatives, and simple flavones (kaempferol, quercetin, myricetin, etc.). The impact of other subgroup constituents from tea on the essential viral proteins remains lacking.

Oolong tea is a semi-fermented tea that offers a flavor profile lying between black and green tea [32], and varying degrees of oxidation diversify the varieties of oolong tea. Rougui Wuyi rock tea (RGWRT) is one of the most common varieties of oolong tea in China. Previous chemical studies on RGWRT utilizing LC-MS analysis have demonstrated the presence of a diverse array of phenolic compounds [1,33]. Therefore, in this study, the chemical constituents of RGWRT were systematically investigated to obtain the various types of tea polyphenols by utilizing a combination of SP825 macroporous resin column, silica gel column and preparative high-performance liquid chromatography. As a result, fifty-eight diverse structures of tea polyphenols, including eleven undescribed compounds, were isolated and elucidated. These compounds could be classified into different categories according to the structural features: such as catechins, procyanidins, flavoalkaloids, theasinensins, quercetin glycosides, myricetin glycosides, as well as kaempferol and its glycosides. Moreover, the potential of various types of isolated tea polyphenols as 3CLpro inhibitors was assessed and the structure-activity relationship was further explored. Finally, the possible mechanism between polyphenols and 3CLpro was preliminarily discussed.

## 2. Results and Discussion

### 2.1. Isolation and LC-MS Analysis of RGWRT

**Figure 1:**
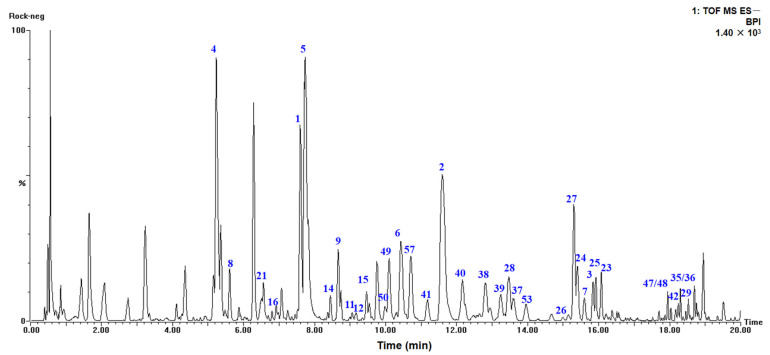
The LC-MS fingerprint of RGWRT in the negative mode and the serial numbers of peaks were consistent with the isolated compounds. The retention times of isolated compounds ranged from 5 to 19 min.

**Figure 2:**
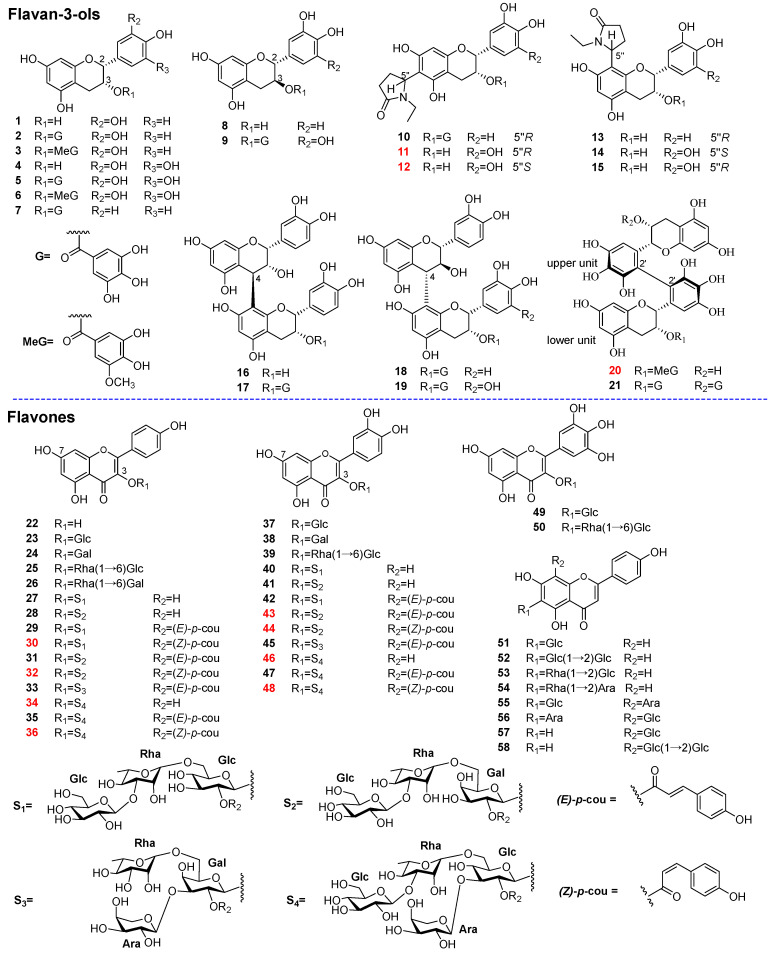
Chemical structures of compounds **1**–**58** from RGWRT. The compounds shown in red numbers are undescribed structures.

In order to obtain the diverse structures of tea polyphenols, the chemical components of RGWRT were systematically studied. As a result, fifty-eight polyphenolic compounds were afforded and could be classified into two main categories: flavan-3-ols (**1**–**21**) and flavones (**22**–**58**). These isolated substances were used as standards in the UPLC-Q-TOF-MS analysis of RGWRT extract. The chromatographic peaks were identified by the comparison with retention time, accurate parent mass, and fragment ion spectrogram of standards, and the results confirmed that the main tea polyphenols have been obtained in the present study (Figure 1). Furthermore, the flavan-3-ols in RGWRT could be further subdivided into several subgroups, including catechins, flavoalkaloids, procyanidins, theasinensins and their corresponding derivatives. The flavones could be further categorized into different classes based on the number of hydroxyl groups, encompassing kaempferol, quercetin, and myricetin, along with their respective *O*-glycosides. The *C*-glycosides of flavone are presented as a distinct subclass. The specific structures of various types of tea polyphenols are illustrated in Figure 2. This is the first time that a wide variety of tea polyphenols with diverse structural types were obtained for sequential studies.

The structures of all polyphenols were elucidated on the basis of comprehensive analysis of 1D and 2D NMR and HRMS spectroscopic data, including eleven undescribed compounds (**11**, **12**, **20**, **30**, **32**, **34**, **36**, **43**, **44**, **46**, and **48**) and forty-seven known tea polyphenols. The known compounds were identified by comparing their spectral data with those reported in the literature as (−)-epicatechin (**1**) [34], (−)-epicatechin-3-*O*-gallate (**2**) [35], (−)-epicatechin-3-(3′-*O*-methyl)-gallate (**3**) [36], (−)-epigallocatechin (**4**) [37], (−)-epigallocatechin-3-*O*-gallate (**5**) [38], (−)-epigallocatechin-3-(3′-*O*-methyl)-gallate (**6**) [39], (−)-epiafzelechin-3-*O*-gallate (**7**) [40], (+)-catechin (**8**) [41], (−)-gallocatechin-3-*O*-gallate (**9**) [38], etc-pyrrolidinone F (**10**) [42], puerin IV (**13**) [19], puerin V (**14**) [19], puerin VI (**15**) [19], procyanidin B2 (**16**) [43], procyanidin B2-3′-*O*-gallate (**17**) [44], procyanidin B4-3′-*O*-gallate (**18**) [45], catechin-(4*α*→8)-epigallocatechin-3-*O*-gallate (**19**) [46], theasinensin A (**21**) [47], kaempferol (**22**) [48], astragalin (**23**) [49], kaempferol-3-*O*-*β*-D-galactopyranoside (**24**) [50], kaempferol-3-*O*-rutinoside (**25**) [51], kaempferol-3-*O*-robinobioside (**26**) [52], kaempferol-3-*O*-[*β*-D-glucopyranosyl-(1→3)-*α*-L-rhamnopyranosyl-(1→6)]-*β*-D-glucopyranoside (**27**) [53], kaempferol-3-*O*-[*β*-D-glucopyranosyl-(1→3)-*α*-L-rhamnopyranosyl-(1→6)]-*β*-D-galactopyranoside (**28**) [54], kaempferol-3-*O*-[2″-*O*-(*E*)-*p*-coumaroyl] [*β*-D-glucopyranosyl-(1→3)-*α*-L-rhamnopyranosyl-(1→6)]-*β*-D-glucopyranoside (**29**) [55], kamoreokchaside I (**31**) [56], camellikaempferoside A (**33**) [57], camellikaempferoside C (**35**) [58], quercetin-3-*O*-*β*-D-glucoside (**37**) [59], quercetin-3-*O*-*β*-D-galactoside (**38**) [59], rutin (**39**) [60], quercetin-3-*O*-[*β*-D-glucopyranosyl-(1→3)-*α*-L-rhamnopyranosyl-(1→6)]-*β*-D-glucopyranoside (**40**) [53], quercetin-3-*O*-[*β*-D-glucopyranosyl-(1→3)-*α*-L-rhamnopyranosyl-(1→6)]-*β*-D-galcopyranoside (**41**) [53], camelliquercetiside B (**42**) [61], camelliquercetiside C (**45**) [61], camelliquercetiside A (**47**) [61], myricetin-3-*O*-*β*-D-glucopyranoside (**49**) [62], myricetin-3-*O*-*β*-D-rutinoside (**50**) [63], isovitexin (**51**) [64], isovitexin-2″-*O*-glucoside (**52**) [65], apigenin-6-*C*-*α*-L-rhamnopyranosyl-(1→2)-*β*-D-glucopyranoside (**53**) [66], apigenin-6-*C*-*α*-L-rhamnopyranosyl-(1→2)-*α*-L-arabinopyranoside (**54**) [67], schaftoside (**55**) [68], isoschaftoside (**56**) [53], vitexin (**57**) [69], vitexin-2″-*O*-glucoside (**58**) [65], respectively.

### 2.2. Structure Elucidation of New Tea Polyphenols from RGWRT

**Figure 3:**
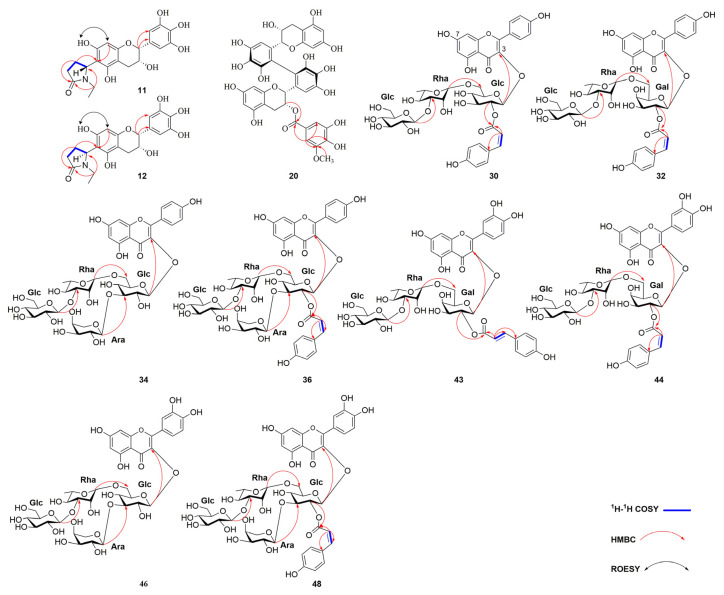
Key ^1^H−^1^H COSY (blue bold bonds), HMBC (red arrows), and ROESY (black double arrows) correlations of the new compounds.

Compound **11** was obtained as white amorphous powder. The molecular formula was deduced as C_21_H_23_NO_8_ from a molecular ion peak at *m/z* 416.1331 [M − H]^−^ (calcd for C_21_H_22_NO_8_, 416.1345) in the HR-ESI-MS. The presence of a flavan-3-ol skeleton was inferred obviously from the ^1^H and ^13^C NMR spectra (recorded in methanol-*d_4_*) of **11** (Table 1, Figure S2) [37]. More specifically, the typical proton signals at *δ*_H_ 6.01 (1H, s) for ring A, at *δ*_H_ 6.51 (2H, s, H-2′, H-6′) for ring B, and at *δ*_H_ 4.76 (1H, s, H-2), *δ*_H_ 4.20 (1H, s, H-3), *δ*_H_ 2.86 (1H, m, H-4*_β_*), *δ*_H_ 2.76 (1H, m, H-4*_α_*) for ring C, were similar to those of (−)-epigallocatechin (EGC) (Table 1). A single proton signal on the A ring indicated a replacement at C-6 or C-8. Besides the similar signals with EGC skeleton, the ^1^H NMR spectrum of **11** indicated the presence of three sets of methylene signals at *δ*_H_ 2.66, 2.42 (each 1H, m) for H-3″, *δ*_H_ 2.17 and 2.34 (each 1H, m) for H-4″, *δ*_H_ 2.66 and 3.50 (each 1H, m) for H-6″, a methine proton at *δ*_H_ 5.45 (1H, s) for H-5″, and one methyl group at *δ*_H_ 1.01 (3H, t, *J* = 7.2 Hz) for H-7″, and ^13^C NMR spectrum showed the corresponding signals attributable to one carbonyl (*δ*_C_ 177.6, C-2″), two methylenes (*δ*_C_ 32.6, 24.5, C-3″ and 4″), a methine (*δ*_C_ 54.1, C-5″), and an ethyl group (*δ*_C_ 36.3, 12.6, C-6″ and 7″), which was further supported by HSQC correlations. The HMBC correlations between H-3″ with C-5″, C-4″, and C-2″ suggested the presence of a *N*-ethyl-2-pyrrolidinone ring [70] (Figure 3). The existence of a partial structure of –C(3″)H_2_−C(4″)H_2_−C(5″)H− could be further inferred through the ^1^H−^1^H COSY correlations of H-4″ with H-3″/H-5″ [42]. The combination of the HMBC correlations between H-4″ to C-6/C-2″/C-5″/C-3″ and H-8 to C-6/C-7/C-9/C-10, as well as the ROESY (recorded in DMSO-*d_6_*) correlation of the signal (*δ*_H_ 5.89, H-8) with the 7-OH proton (*δ*_H_ 9.23) (Figure S8) showed that the *N*-ethyl-2-pyrrolidinone moiety was connected to the C-6 position [71]. Thus, the planar structure of compound **11** was determined as 6-*N*-ethyl-2-pyrrolidinone-epigallocatechin.

Compound **12** was found to have the same molecular formula as **11** (C_21_H_23_NO_8_) based on a molecular ion peak at *m/z* 416.1336 [M − H]^−^ (calcd for C_21_H_22_NO_8_, 416.1345) in the HR-ESI-MS. The ^1^H NMR and ^13^C NMR spectra of **12** were similar to those of **11**, indicating they possess the same structural scaffold. The position of *N*-ethyl-2-pyrrolidinone in compound **12** was also determined to be at C-6 via the similar key correlations of ROESY (recorded in DMSO-*d*_6_, Figure S17) and HMBC spectra (recorded in methanol-*d_4_*, Figure S16).

**Figure 4:**
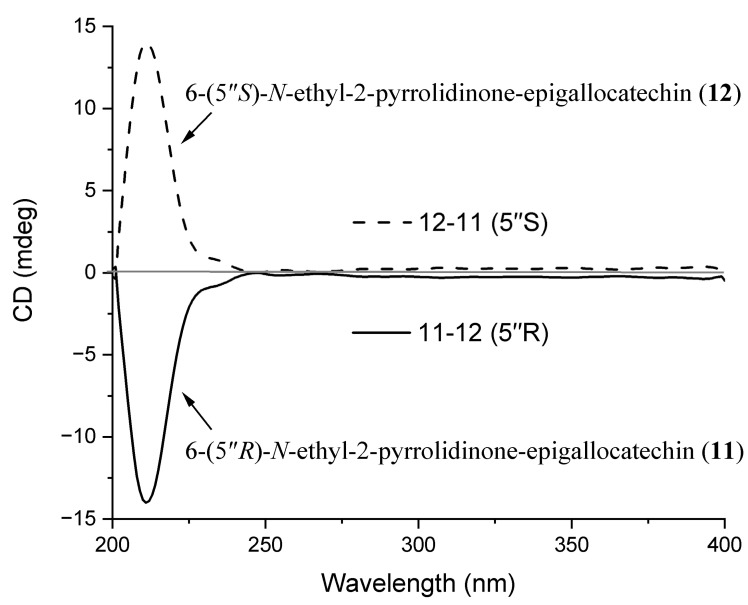
The arithmetically CD curves subtracted from each other for compounds **11** and **12**.

The difference between **12** and **11** is the configuration of C-5″, and previous studies have shown that the absolute configuration at C-5″ can be distinguished by subtracting one CD spectrum from its stereoisomer with the same configurations at C-2/C-3 [19,42]. To identify the absolute configuration at C-5″ of compounds **11** and **12**, a pair of stereoisomers at C-5″, the CD spectra of two compounds were compared after subtracting the CD spectrum from each other (Figure 4) [42]. For compound **11**, the arithmetically independent CD curves of C-5″ exhibited a strong negative cotton effect at 211 nm (Δε − 14.0) and was confirmed to be of the C-5″ *R* configuration. The configuration of **12** was deduced as *S* configuration due to a positive cotton effect at 211 nm (Δε + 14.0) in the CD spectrum (Figure 4). Therefore, the structures of compound **11** and **12** were determined to be 6-(5″*R*)-*N*-ethyl-2-pyrrolidinone-epigallocatechin and 6-(5″*S*)-*N*-ethyl-2-pyrrolidinone-epigallocatechin.

Compound **20** was determined to have a molecular formula of C_38_H_32_O_18_ based on HR-ESI-MS at *m/z* 775.1505 [M − H]^−^ (calcd for C_38_H_31_O_18_, 775.1510). The ^1^H and ^13^C NMR data of **20** were similar to theasinensin B except for the addition of a methoxy group [*δ*_H_ 3.81, (3H, s)], indicating that **20** was a methylated theasinensin B [45]. The HMBC correlations from the methoxy proton to C-3″, and from H-2″ (*δ*_H_ 6.99) to C-1″/C-3″/C-4″/C-6″/C-7″ (Figure 3) confirmed that the methoxy group was linked to C-3″. Therefore, the structure of compound **20** was assigned as epigallocatechin-(2′→2′)-epigallocatechin-3-(3″-*O*-methyl)-gallate.

Compound **30** was presented as yellow amorphous powder with a molecular formula of C_42_H_46_O_22_ by HR-ESI-MS ion at *m/z* 901.2394 [M − H]^−^ (calcd for C_42_H_45_O_22_, 901.2402). The ^1^H NMR and ^1^H−^1^H COSY spectrum of **30** suggested the presence of characteristic kaempferol aglycone [*δ*_H_ 6.19, (1H, d, *J* = 2.0 Hz) and 6.38 (1H, d, *J* = 2.0 Hz) for A ring; *δ*_H_ 6.91 (2H, d, *J* = 8.7 Hz) and 8.00 (2H, d, *J* = 8.7 Hz) for B ring], *Z*-*p*-coumaroyl group [*δ*_H_ 6.70 (2H, d, *J* = 8.5 Hz), 7.64 (2H, d, *J* = 8.6 Hz), 5.89 (1H, d, *J* = 12.7 Hz) and 6.88 (1H, d, *J* = 12.7 Hz)] [72], and three monocarbohydrate moieties [*δ*_H_ 4.41 (1H, d, *J* = 7.7 Hz), 4.55 (1H, br s) and 5.51 (1H, d, *J* = 8.0 Hz) for three anomeric protons] (Table 2). In addition, the corresponding anomeric carbons of the sugars were observed at *δ*_C_ 105.7, 102.1 and 100.7 in the ^13^C NMR spectrum, with the help of the HSQC experiment. Acid hydrolysis of **30** followed by UPLC-CAD analysis identified two D-glucose, and a L-rhamnose. The key HMBC correlations of H-1″ (*δ*_H_ 5.51, Glc) to C-3 (*δ*_C_ 134.6), H-1‴ (*δ*_H_ 4.55, Rha) to C-6″ (*δ*_C_ 68.2, Glc), and H-1′′′′ (*δ*_H_ 4.41, Glc) to C-3‴ (*δ*_C_ 83.2, Rha) determined the linkage of sugar chain [55,56]. The *p*-coumaroyl group was attached at C-2″ by the correlation between *δ*_H_ 5.00 (H-2″) to *δ*_C_ 167.6 (C=O of *Z*-*p*-coumaroyl group). Therefore, the chemical structure of **30** was determined to be kaempferol-3-*O*-[2″-*O*-(*Z)*-*p*-coumaroyl] [*β*-D-glucopyranosyl-(1→3)-*α*-L-rhamnopyranosyl-(1→6)]-*β*-D-glucopyranoside.

Compound **32**, a yellow amorphous powder, showed an anion peak at *m/z* 901.2384 [M − H]^−^ (C_42_H_45_O_22_, 901.2402) in HR-ESI-MS spectrum, exhibited similar ^1^H and ^13^C NMR spectra compared to **30**. The main difference was that the glucopyranoside attached at C-3 in **30** was replaced by a galactopyranoside moiety, which was confirmed by the acid hydrolysis and the anomeric proton [*δ*_H_ 5.41 (1H, d, *J* = 7.9 Hz)]. Thus, compound **32** was elucidated as kaempferol-3-*O*-[2″-*O*-(*Z*)-*p*-coumaroyl] [*β*-D-glucopyranosyl-(1→3)-*α*-L-rhamnopyranosyl-(1→6)]-*β*-D-galactopyranoside.

Compound **34**, a yellow amorphous powder, was determined as C_38_H_48_O_24_ by HR-ESI-MS at *m/z* 887.2448 [M − H]^−^ (calcd for C_38_H_47_O_24_, 887.2457). The ^1^H and ^13^C NMR data of **34** were similar to those of **30** except for the addition of a sugar unit at C-3″ and the absence of *p*-coumaroyl group, indicating that **34** was a kaempferol tetraglycoside. The presence of two *β*-D-glucopyranosyl, one *α*-L-arabinopyranosyl, and one *α*-L-rhamnopyranosyl units were confirmed by the acid hydrolysis and coupling constants. The glycosidic linkage of oligosaccharide chain was established via the key HMBC correlations from *δ*_H_ 5.17 (H-1″, Glc) to *δ*_C_ 135.5 (C-3), *δ*_H_ 4.56 (H-1‴, Rha) to *δ*_C_ 68.8 (C-6″, Glc), *δ*_H_ 4.40 (H-1′′′′, Glc) to *δ*_C_ 83.2 (C-3‴, Rha), and *δ*_H_ 4.57 (H-1′′′′′, Ara) to *δ*_C_ 86.9 (C-3″, Glc) (Figure 3). Thus, the structure of **34** was determined to be kaempferol-3-*O*-[*α*-L-arabinopyranosyl-(1→3)] [*β*-D-glucopyranosyl-(1→3)-*α*-L-rhamnopyranosyl-(1→6)]-*β*-D-glucopyranoside.

Compound **36** was purified as a yellow amorphous powder, and its molecular formula was deduced as C_47_H_54_O_26_ by HR-ESI-MS at *m/z* 1033.2805 [M − H]^−^ (calcd for C_47_H_53_O_26_, 1033.2825). The NMR data obtained for **36** resembled those of **30**, except for the additional arabinopyranosyl unit attached at C-3″, which was supported by the acid hydrolysis and HMBC correlation of H-1′′′′′ (*δ*_H_ 4.30) to C-3″ (*δ*_C_ 80.5). The *α*-configuration of the arabinopyranosyl group was determined by the coupling constant (*J* = 5.9 Hz) of the anomeric proton. Consequently, compound **36** was elucidated as kaempferol-3-*O*-[2″-*O*-(*Z*)-*p*-coumaroyl] [*α*-L-arabinopyranosyl-(1→3)] [*β*-D-glucopyranosyl-(1→3)-*α*-L-rhamnopyranosyl-(1→6)]-*β*-D-glucopyranoside.

Compound **43** was presented as yellow amorphous powder with a molecular formula of C_42_H_46_O_23_ by HR-ESI-MS ion at *m/z* 917.2331 [M − H]^−^ (calcd for C_42_H_45_O_23_, 917.2352). The ^1^H NMR spectrum indicated the typical quercetin unit signals at *δ*_H_ 7.46 (1H, d, *J* = 2.2 Hz, H-2′), *δ*_H_ 7.63 (1H, dd, *J* = 8.5, 2.3 Hz, H-6′), *δ*_H_ 6.79 (1H, m, H-5′), *δ*_H_ 6.29 (1H, br s, H-8) and *δ*_H_ 6.11 (1H, br s, H-6), and *E*-*p*-coumaroyl group with protons at *δ*_H_ 7.56 (1H, d, *J* = 15.9 Hz, H-Cou-7), *δ*_H_ 7.52 (2H, d, *J* = 8.6 Hz, H-Cou-2, H-Cou-6), *δ*_H_ 6.79 (2H, d, *J* = 8.4 Hz, H-Cou-3, H-Cou-5), and *δ*_H_ 6.38 (1H, d, *J* = 15.9 Hz, H-Cou-8) [72]. Furthermore, three anomeric proton signals of sugar moieties were observed at *δ*_H_ 5.59 (1H, d, *J* = 8.0 Hz, H-1″), *δ*_H_ 4.49 (1H, br s, H-1‴), and *δ*_H_ 4.32 (1H, d, *J* = 7.7 Hz, H-1′′′′) (Table 2). 2D NMR and acid hydrolysis experiments were performed to determine the sugar unit and the glycosidic linkage of **43**, which suggested the existence of a *β*-D-galactopyranosyl, an *α*-L-rhamnopyranosyl, and *β*-D-glucopyranosyl moieties. The attachments of sugar chains and *E*-*p*-coumaroyl group were determined by the HMBC correlations of *δ*_H_ 5.59 (H-1″, Gal) to *δ*_C_ 132.8 (C-3), *δ*_H_ 4.49 (H-1‴, Rha) to *δ*_C_ 64.9 (C-6″, Gal), *δ*_H_ 4.32 (H-1′′′′, Glc) to *δ*_C_ 81.7 (C-3‴, Rha) and *δ*_H_ 5.22 (H-2″, Gal) to δ_C_ 166.0 (C=O of *E*-*p*-coumaroyl group) [61]. Accordingly, compound **43** was identified as quercetin-3-*O*-[2″-*O*-(*E*)-*p*-coumaroyl] [*β*-D-glucopyranosyl-(1→3)-*α*-L-rhamnopyranosyl-(1→6)]-*β*-D-galcopyranoside.

Compound **44** was obtained as a yellow amorphous powder, and its molecular formula was deduced as C_42_H_46_O_23_ by HR-ESI-MS at *m/z* 917.2332 [M − H]^−^ (calcd forC_42_H_45_O_23_, 917.2352). The ^1^H and ^13^C NMR data of **44** exhibited high similarity to that of **43**, with the main distinction being a configuration of coumaroyl moiety shift from *E*- in **43** to *Z*- in **44** (Table 3), which was supported by the two olefinic protons at *δ*_H_ 6.82 (1H, d, *J* = 12.0 Hz) and *δ*_H_ 5.81 (1H, d, *J* = 12.9 Hz). Hence, the structure of compound **44** was established as quercetin-3-*O*-[2″-*O*-(*Z*)-*p*-coumaroyl] [*β*-D-glucopyranosyl-(1→3)-*α*-L-rhamnopyranosyl-(1→6)]-*β*-D-galcopyranoside.

Compound **46** was isolated as a yellow amorphous powder with a molecular formula of C_38_H_48_O_25_ by HR-ESI-MS ion at *m/z* 903.2400 [M − H]^−^ (calcd for C_38_H_47_O_25_, 903.2406). The ^1^H and ^13^C NMR spectroscopic data of **46** were similar to those of **44** except for the addition of an arabinopyranosyl unit at C-3″ and the absence of *Z*-*p*-coumaroyl group, indicating that **46** was a quercetin tetraglycoside, which was further confirmed by the acid hydrolysis. The attachment of an arabinopyranosyl group to C-3″ was established from the HMBC correlation between H-3″ (*δ*_H_ 3.60, Glc) with *δ*_C_ 105.6 (H-1′′′′′, Ara). Consequently, compound **46** was elucidated as quercetin-3-*O*-[*α*-L-arabinopyranosyl-(1→3)] [*β*-D-glucopyranosyl-(1→3)-*α*-L-rhamnopyranosyl-(1→6)]-*β*-D-glucopyranoside.

Compound **48**, a yellow amorphous powder, was determined as C_47_H_54_O_27_ by HR-ESI-MS at *m/z* 1049.2751 [M − H]^−^ (calcd for C_47_H_53_O_27_, 1049.2774). Compound **48** showed similar NMR data to that of camelliquercetiside A [62], except for slight differences of the two olefinic proton signals at *δ*_H_ 6.82 (1H, d, *J* = 13.3 Hz) and *δ*_H_ 5.83 (1H, d, *J* = 12.8 Hz), indicating the different configuration of the double bond in **48**. Moreover, the linkage of the *Z*-*p*-coumaroyl unit was assigned at C-2″, which was confirmed through the HMBC correlation from H-2″ (*δ*_H_ 5.04, m) to C-Cou 9 (the carbonyl carbon of the *Z*-coumaroyl moiety, *δ*_C_ 164.9). Therefore, the structure of compound **48** was characterized as quercetin-3-*O*-[2″-*O*-(*Z*)-*p*-coumaroyl] [*α*-L-arabinopyranosyl-(1→3)] [*β*-D-glucopyranosyl-(1→3)-*α*-L-rhamnopyranosyl-(1→6)]-*β*-D-glucoside.

### 2.3. Inhibitory Activities of Diverse Structures of Tea Polyphenols on 3CLpro

**Figure 5:**
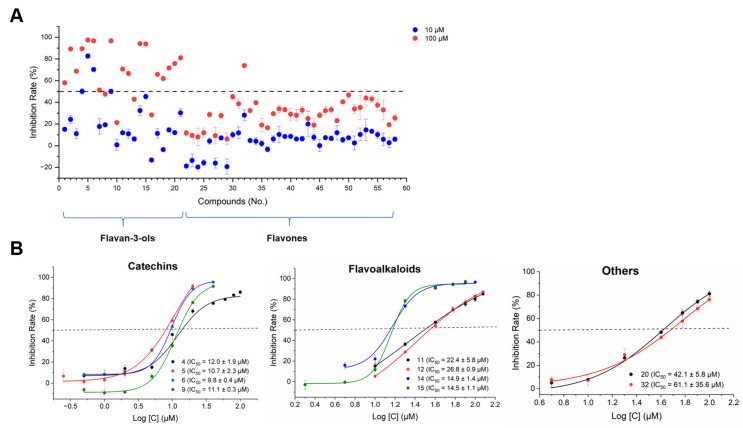
Inhibition rate (%) of 58 tea polyphenols against SARS-CoV-2 3CLpro (**A**), and representative inhibition curves for catechin derivatives (compounds **4**, **5**, **6**, and **9**), flavoalkaloid derivatives (compounds **11**, **12**, **14**, and **15**) and other types of polyphenols (compounds **20** and **32**) against SARS-CoV-2 3CLpro (**B**).

All the isolated tea polyphenols with diverse structures were preliminarily evaluated for their inhibitory activities against 3CLpro at concentrations of 10 μM and 100 μM using the FRET-based protease assay (Figure 5A). The results showed that among the isolates, compounds **4**−**6**, and **9**, exhibited significant inhibition rates of over 50% at a concentration of 10 μM. Additionally, another fourteen compounds (**1**−**3**, **7**, **11**, **12**, **14**, **15**, **17**−**21**, and **32**) showed inhibition rates exceeding 50% at a concentration of 100 μM, as illustrated in Figure 5A. Of these eighteen active phenolic compounds, except for compound **32** (a new acylated flavones glycoside), the other seventeen compounds were classified as flavan-3-ols, specifically including catechins (**1**−**7**, **9**), flavoalkaloids (**11**, **12**, **14**, and **15**), procyanidins (**17**−**19**), and theasinensins (**20**, **21**). Subsequently, ten representative bioactive compounds with various structures were further selected to assess their concentration-dependent manners and IC_50_ values. Among these, four catechin derivatives (**4**−**6**, and **9**) demonstrated significant inhibition of 3CLpro in a concentration-dependent manner, with IC_50_ values ranging from 9.8 μM to 12.0 μM. It is worth mentioning that EGCG (**5**), the most researched tea polyphenol, was indeed found to possess promising inhibitory activity with IC_50_ values of 10.7 μM which was consistent with the reported literature [73]. However, in the present study, compound **6**, (−)-epigallocatechin-3-(3′-*O*-methyl)-gallate, was first discovered to exhibit a more potent inhibitory effect on 3CLpro activity than EGCG. Additionally, four flavoalkaloids (**11**, **12**, **14**, and **15**) displayed moderate inhibitory activity against 3CLpro with IC_50_ values ranging from 14.5 μM to 26.8 μM. The remaining two compounds, **20** and **32**, showed an inhibition with IC_50_ values of 42.1 μM, and 61.1 μM, respectively. (Figure 5B).

### 2.4. Structure–Activity Relationship Analysis

The relationship between polyphenol structures and their anti-3CLpro activities was further investigated. First, it can be concluded that flavan-3-ols exhibit relatively superior inhibitory activity against 3CLpro compared to flavones. In tea, flavones primarily exist as the corresponding glycosides [1,33] and the steric hindrance of these compounds increases with the introduction of one or more glycosyl groups at the C-3/C-6/C-8 position, which negatively affects their activity [74,75]. Particularly, the flavones isolated in the present study primarily contained more than two glucoside groups. Second, among the various subgroups of flavan-3-ols, ester-type catechin derivatives, featuring a galloyl moiety at the C-3 position, exhibited a notably stronger inhibitory effect on 3CLpro than other categories. The comparisons of compounds **5** with **4**, **2** with **1**, and **17** with **16** revealed the enhanced effect of the galloyl group at C-3. These results are consistent with previous reports that the presence of a galloyl group at C-3 boosts the antiviral activity of flavan-3-ols [75]. Furthermore, most of the flavoalkaloids examined in this study were found to exhibit moderate inhibitory effects on 3CLpro, and those with the *N*-ethyl-2-pyrrolidinone group attaching at C-8 displayed superior inhibitory activity compared to those attaching at C-6 (comparing **14** and **15** to **11** and **12**), presumably because of their smaller steric hindrance [76]. However, when the *N*-ethyl-2-pyrrolidinone group was introduced into catechins, their inhibitory effects were further attenuated (comparing **11**, **12**, **14**, and **15** to **4**). It should be mentioned that although various structural polyphenols were obtained from RGWRT, another important polyphenols—theaflavins, which mainly exist in fermented tea—were not found in the current study, and further analysis is worthy to investigate.

### 2.5. Molecular Docking of Representative Flavan-3-Ols Against SARS-CoV-2 3CLpro

**Figure 6:**
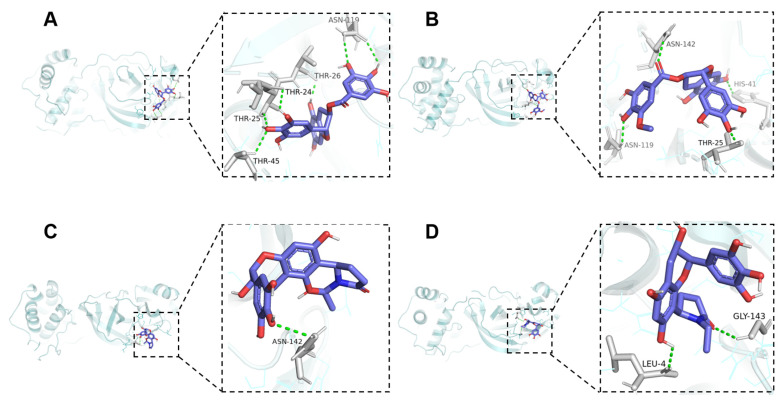
The docking mode and hydrogen bond interactions between the selected bioactive compounds and 3CLpro. (**A**): compound **5**, (**B**): compound **6**, (**C**): compound **11**, (**D**): compound **15**.

To explore the possible mechanism underlying the anti-3CLpro activity of catechin and flavoalkaloid derivatives, four selected phenolic compounds were subjected to molecular docking using AMDock v1.5.2 software. The results revealed that these bioactive compounds exhibited favorable affinities with 3CLpro (docking score < −7 kcal/mol) (Table S1), which aligned with the in vitro assays. As shown in Figure 6, the hydrogen bond interactions between compounds **5** and **6**, two of the most active catechins, with the protein were attributed to their interactions with the residues of amino acids ASN142, ASN119, THR25, and HIS41, contributing to the formation of a stable complex with 3CLpro. The result showed that these residues were the key amino acids for the interaction between catechin derivatives and 3CLpro [26,30]. Among the two active flavoalkaloids, compound **15** formed hydrogen bond interactions with residues LEU4 and GLY143, while **11** interacted with ASN142. The above findings indicated that the differing affinities of these two types of flavan-3-ols on 3CLpro stem from the variations in the number and positions of the binding interactions with the protein. The 3CLpro of SARS-CoV-2 is essential for viral transcription and replication. Apart from some main compounds, such as EGCG, TF, kaempferol, etc, other phenolic compounds have not been thoroughly investigated, or only subjected to virtual screening analysis, for their 3CLpro inhibitory activity due to difficulty in obtaining monomers [23,24]. Our findings firstly revealed the potential of other tea polyphenols as inhibitors for 3CLpro, particularly flavoalkaloid derivatives. However, the inhibitory activities of isolated polyphenols were assessed through the 3CLpro enzyme activity inhibition test in vitro, and it is worthwhile to conduct further additional experiments focusing on other antiviral experiments (in vivo validation and other targets).

## 3. Materials and Methods

### 3.1. Instrumentation

Optical rotation values were acquired on an Anton Paar MCP 200 Automatic Polarimeter (Anton Paar Co., Graz, Austria). NMR spectroscopic data were obtained using a Bruker Avance III 600 NMR spectrometer (Bruker Co., Bremen, Germany). HR-ESI-MS analyses were performed on a Waters Synapt G2-S Q-TOF mass spectrometer (Waters Co., Milford, MA, USA). Circular dichroism (CD) spectra were measured using a Chirascan spectropolarimeter (Applied Photophysics Ltd., Leatherhead, UK). Liquid chromatography analysis was carried out on a Vanquish Flex UPLC instrument coupled with a Charged Aerosol Detection (CAD) (Thermo Fisher Scientific, Waltham, MA, USA) using a Welch Ultimate XB C18 column (5 μm, 250 × 4.6 mm, Welch Materials, Inc. Shanghai, China), an Agela Venusil MP C18 column (5 μm, 250 × 4.6 mm, Agela Technologies, Tianjin, China), a ChromCore Phenyl column (5 μm, 250 × 4.6 mm, NanoChrom Technologies Co., Ltd., Suzhou, China), a Lux i-cellulose column (5 μm, 250 × 4.6 mm, Phenomenex Co., Torrance, CA, USA), Phenomenex Kinetex C18 column (2.6 μm, 150 × 4.6 mm, Phenomenex Co., Torrance, CA, USA), two Silgreen C18 columns (5 μm, 150 × 4.6 mm; 5 μm, 250 × 4.6 mm; Beijing Greenherbs Science & Technology Development Co., Ltd., Beijing, China). Semi-preparative HPLC was performed on a Hannon Newstyle NP7000 pump (Hanbon Co. Ltd., Huaian, China) equipped with a Shodex RID 101 detector (Showa Denko., Tokyo, Japan) using preparative chromatographic columns corresponding to the analytical columns mentioned above. Preparative HPLC was conducted using two Silgreen C18 columns (5 μm, 150 × 21.2 mm; 5 μm, 250 × 20 mm; Beijing Greenherbs Science & Technology Development Co., Ltd., Beijing, China) on a Hannon Newstyle NP7000 pump (Hanbon Co. Ltd., Huaian, China) equipped with a Shodex RID 102 detector (Showa Denko., Tokyo, Japan). Preparative MPLC was carried out using BUCHI Flash Pure C-850 apparatus (BUCHI Labortechnik AG, Flawil, Switzerland) with Flash Pure columns (FP ECOFLEX C18 220g or FP ECOFLEX C18 800g, BUCHI Labortechnik AG, Flawil, Switzerland). Column chromatography (CC) was carried out on macroporous resin SP825 (Mitsubishi Chemical Holdings, Tokyo, Japan), Silica gel (200–300 mesh, Qingdao Marine Chemical Co. Ltd., Qingdao, China) and ODS silica gel (50 μm, YMC, Kyoto, Japan). TLC analysis was performed on silica gel GF254 plates (Tianjin Silida Technology Co. Ltd., Tianjin, China).

### 3.2. Plant Materials, Chemicals and Reagents

Rougui Wuyi rock tea was purchased from Wuyishan Nanhu Ecological Tea Co., Ltd. (Nanping, China). Methanol-*d_4_* and dimethyl sulfoxide (DMSO)-*d_6_* were bought from Cambridge Isotope Laboratories, Inc. (Tewksbury, MA, USA). The sugar standards, including D-glucose, D-galactose, L-rhamnose and L-arabinose were purchased from Shanghai Macklin Biochemical Co., Ltd. (Shanghai, China). Trifluoroacetic acid (Thermo Scientific, Waltham, MA, USA), anhydrous pyridine (Sinopharm Chemical Reagent Co., Ltd., Shanghai, China), L-cysteine methyl ester hydrochloride (Shanghai Macklin Biochemical Co., Ltd., Shanghai, China) and O-tolyl isothiocyanate (Shanghai Macklin Biochemical Co., Ltd., Shanghai, China) were used in the acid hydrolysis experiment. Chromatographic methanol, formic acid and acetonitrile for HPLC and LC-MS were obtained from Thermo Fisher Scientific (Waltham, MA, USA). Analytical grade ethanol, petroleum ether, ethyl acetate, *n*-butanol, dichloromethane, methanol, and acetonitrile for extraction, separation and purification were purchased from Sinopharm chemical reagent Co., Ltd. (Shanghai, China). The 2019-nCoV Mpro/3CLpro inhibitor screening kit (P0312M) was a commercial kit which is bought from Beyotime Biotechnology (Shanghai, China).

### 3.3. Extraction, Isolation and Acid Hydrolysis of Isolated Compounds

Rougui Wuyi rock tea (3 kg) was comminuted and extracted by soaking with 70% aqueous EtOH three times (each for 24 h). The extracted solution was concentrated under reduced pressure, then dispersed with water, and subsequently extracted with petroleum ether, ethyl acetate and *n*-butanol to obtain four fractions, Fr. A (80.5 g), Fr. B (82.6 g), Fr. C (615.0 g), and Fr. D (39.1 g). By utilizing a combination of SP825 macroporous resin column, silica gel column and preparative HPLC, fifty-eight tea polyphenols were obtained from four fractions. The detailed procedure of isolation and acid hydrolysis experiment is shown in the Supporting Materials.

### 3.4. Physicochemical Properties and Spectroscopic Data of New Compounds

*6-(5″R)-N-ethyl-2-pyrrolidinone-epigallocatechin* (**11**): white amorphous powder; ${\left[\alpha \right]}_{D}^{25}$ − 40.99 (*c* 0.11, MeOH); ^1^H and ^13^C NMR data, see Table 1; HR-ESI-MS *m/z* 416.1331 [M − H]^−^ (calcd for C_21_H_22_NO_8_, 416.1345).

*6-(5″S)-N-ethyl-2-pyrrolidinone-epigallocatechin* (**12**): white amorphous powder; ${\left[\alpha \right]}_{D}^{25}$ + 20.69 (*c* 0.06, MeOH); ^1^H and ^13^C NMR data, see Table 1; HR-ESI-MS *m/z* 416.1336 [M − H]^−^ (calcd for C_21_H_22_NO_8_, 416.1345).

*Epigallocatechin-(2′→2′)-epigallocatechin-3-(3″-O-methyl)-gallate* (**20**): brown amorphous powder; ${\left[\alpha \right]}_{D}^{25}$ − 67.99 (*c* 0.10, MeOH); ^1^H and ^13^C NMR data, see Table 1; HR-ESI-MS *m/z* 775.1505 [M − H]^−^ (calcd for C_38_H_31_O_18_, 775.1510).

*Kaempferol-3-O-[2″-O-(Z)-p-coumaroyl] [β-D-glucopyranosyl-(1→3)-α-L-rhamnopyranosyl-(1→6)]-β-D-glucopyranoside* (**30**): yellow amorphous powder; ${\left[\alpha \right]}_{D}^{25}$ − 58.32 (*c* 0.07, MeOH); ^1^H and ^13^C NMR data, see Table 2 and Table 3; HR-ESI-MS *m/z* 901.2394 [M − H]^−^ (calcd for C_42_H_45_O_22_, 901.2402).

*Kaempferol-3-O-[2″-O-(Z)-p-coumaroyl] [β-D-glucopyranosyl-(1→3)-O-α-L-rhamnopyranosyl-(1→6)]-β-D-galactopyranoside* (**32**): yellow amorphous powder; ${\left[\alpha \right]}_{D}^{25}$ − 87.99 (*c* 0.16, MeOH); ^1^H and ^13^C NMR data, see Table 2 and Table 3; HR-ESI-MS *m/z* 901.2384 [M − H]^−^ (calcd for C_42_H_45_O_22_, 901.2402).

*Kaempferol-3-O-[α-L-arabinopyranosyl-(1→3)] [β-D-glucopyranosyl-(1→3)-α-L-rhamnopyranosyl-(1→6)]-β-D-glucopyranoside* (**34**): yellow amorphous powder; ${\left[\alpha \right]}_{D}^{25}$ − 32.99 (*c* 0.10, MeOH); ^1^H and ^13^C NMR data, see Table 2 and Table 3; HR-ESI-MS *m/z* 887.2448 [M − H]^−^ (calcd for C_38_H_47_O_24_, 887.2457).

*Kaempferol-3-O-[2″-O-(Z)-p-coumaroyl] [α-L-arabinopyranosyl-(1→3)] [β-D-glucopyranosyl-(1→3)-α-L-rhamnopyranosyl-(1→6)]-β-D-glucopyranoside* (**36**): yellow amorphous powder; ${\left[\alpha \right]}_{D}^{25}$ − 129.14 (*c* 0.07, MeOH); ^1^H and ^13^C NMR data, see Table 2 and Table 3; HR-ESI-MS *m/z* 1033.2805 [M − H]^−^ (calcd for C_47_H_53_O_26_, 1033.2825).

*Quercetin-3-O-[2″-O-(E)-p-coumaroyl] [β-D-glucopyranosyl-(1→3)-α-L-rhamnopyranosyl-(1→6)]-β-D-galcopyranoside* (**43**): yellow amorphous powder; ${\left[\alpha \right]}_{D}^{25}$ − 60.99 (*c* 0.15, MeOH); ^1^H and ^13^C NMR data, see Table 2 and Table 3; HR-ESI-MS *m/z* 917.2331 [M − H]^−^ (calcd for C_42_H_45_O_23_, 917.2352).

*Quercetin-3-O-[2″-O-(Z)-p-coumaroyl] [β-D-glucopyranosyl-(1→3)-α-L-rhamnopyranosyl-(1→6)]-β-D-galcopyranoside* (**44**): yellow amorphous powder; ${\left[\alpha \right]}_{D}^{25}$ − 12.88 (*c* 0.13, MeOH); ^1^H and ^13^C NMR data, see Table 2 and Table 3; HR-ESI-MS *m/z* 917.2332 [M − H]^−^ (calcd forC_42_H_45_O_23_, 917.2352).

*Quercetin-3-O-[α-L-arabinopyranosyl-(1→3)] [β-D-glucopyranosyl-(1→3)-α-L-rhamnopyranosyl-(1→6)]-β-D-glucopyranoside* (**46**): yellow amorphous powder; ${\left[\alpha \right]}_{D}^{25}$ + 8.00 (*c* 0.11, MeOH); ^1^H and ^13^C NMR data, see Table 2 and Table 3; HR-ESI-MS *m/z* 903.2400 [M − H]^−^ (calcd for C_38_H_47_O_25_, 903.2406).

*Quercetin-3-O-[2″-O-(Z)-p-coumaroyl] [α-L-arabinopyranosyl-(1→3)] [β-D-glucopyranosyl-(1→3)-α-L-rhamnopyranosyl-(1→6)]-β-D-glucoside* (**48**): yellow amorphous powder; ${\left[\alpha \right]}_{D}^{25}$ − 49.99 (*c* 0.09, MeOH); ^1^H and ^13^C NMR data, see Table 2 and Table 3; HR-ESI-MS *m/z* 1049.2751 [M − H]^−^ (calcd for C_47_H_53_O_27_, 1049.2774).

### 3.5. LC-MS/MS Analysis of the Tea Extract and Isolated Compounds

UPLC-Q-TOF-MS analysis was performed on a Waters Acquity UPLC system equipped with a Synapt G2-S Q-TOF mass spectrometer (Waters Co., Milford, MA, USA). A Waters Acquity HSS T3 C18 column (1.8 μm, 100 × 2.1 mm) was set at a column temperature of 45 °C. The UPLC and MS conditions were consistent with those reported previously [33]. The instrument was controlled using MassLynx 4.1 software (Waters Corp, Milford, MA, USA).

### 3.6. 3CLpro Enzyme Activity Inhibition Test

To assess the potential inhibitory activity of the isolated compounds against SARS-CoV-2 3CLpro, a commercial kit based on fluorescence resonance energy transfer (FRET) protease assay was used. The kit contains assay buffer (125 mL, blank control), 2019-nCoV-2 3CLpro (500 μL), substrate (1 mL), ebselen (10 mM, 100 μL, positive control), and an instruction manual. Firstly, all the compounds were dissolved in DMSO to obtain two tested solutions with concentrations of 10 μM and 100 μM. According to the operating manual, the assay buffer and 3CLpro were mixed at a ratio of 92:1 to obtain the assay reagent. The 100% enzyme activity control was prepared by uniformly mixing 93 μL assay reagent, 5 μL DMSO and 2 μL substrate. Relative Fluorescence Unit (RFU) was measured using a Synergy LX multi-mode reader (Agilent BioTek, Santa Clara, CA, USA) with excitation at 360 nm and emission at 485 nm. The anti-3CLpro activity was detected three times for each compound at 10 μM and 100 μM concentrations. The inhibition percentage was calculated by the following formula: inhibition rate (%) = (RFU_100% enzyme activity control_ − RFU_sample_)/(RFU_100% enzyme activity control_ − RFU _blank control_) × 100%. Finally, these compounds with inhibition rates of over 50% were screened out to assess their concentration-dependent manners and 50% inhibition concentration (IC_50_) values after serial dilution of the selected compounds. The fluorescence intensity results were analyzed using Origin 2018 software, and IC_50_ values were calculated by drawing a curve of inhibition percentage against the concentration of the compound.

### 3.7. Molecular Docking

Molecular docking was further carried out to detect the relative binding energies and possible binding sites between the bioactive polyphenols and 3CLpro using AMDock version 1.5.2. AMDock integrates capabilities from Autodock Vina, Autodock4, Autodock4Zn and PyMol v.2.1.0 [77]. The crystal structure of 3CLpro (PDB ID: 6LU7) was downloaded from the RCSB PDB protein structure database (http://www.rcsb.org, accessed on 20 February 2025) [78]. The three-dimensional (3D) structures of the bioactive tea polyphenols were constructed by the Chem 3D 18.0 software (PerkinElmer Informatics, Inc., Shelton, CT, USA). After the AutoDock Vina engine was selected for molecular docking simulation, the affinity (kcal/mol) and Estimated Ki values (μM) of the different binding poses could be obtained. The pose with the lowest docking score was chosen as the initial conformation of docking complexes. Finally, graphical visualizations of the protein-ligand complexes were made using PyMol v.2.1.0.

## 4. Conclusions

In conclusion, fifty-eight tea polyphenols, including eleven undescribed compounds, were isolated and elucidated from RGWRT. These compounds could be structurally classified into eight subtypes, which basically cover the representative structure characteristics of tea polyphenols. Flavan-3-ols exhibited relatively superior inhibitory activity against 3CLpro compared to flavones, particularly catechins and flavoalkaloids. Furthermore, their inhibitory effects could be enhanced with the introduction of the galloyl group. The results from molecular docking demonstrated that the differing inhibitory effects of catechins and flavoalkaloids were attributed to the variations in the number and positions of the hydrogen bond interactions with 3CLpro. In summary, these findings not only enrich the chemical structure diversity of tea, but may also be considered as potential anti-3CLpro natural products.

## Figures and Tables

**Table 1:** ^1^H (600MHz) and ^13^C (150MHz) NMR data of compounds **11**, **12** and **20** in methanol-*d_4_* (*δ* in ppm, *J* in Hz).

No.	11		12		20			
	*δ*H (J in Hz)	*δ*C	*δ*H (J in Hz)	*δ*C	*δ*H (J in Hz)	*δ*C	*δ*H (J in Hz)	*δ*C
					Upper Unit		Lower Unit	
2	4.76, s	79.7	4.76, br s	79.7	4.52, s	77.5	4.77, s	76.5
3	4.20, s	67.3	4.20, s	67.2	3.93, d (3.0)	65.5	5.29, d (3.4)	69.4
4	2.86, m, 2.76, m	29.7	2.86, m, 2.70, m	29.5	2.63, d (16.9), 2.34, dd (16.7, 4.7)	29.2	2.81, d (17.2), 2.52, dd (17.4, 4.6)	26.8
5		156.5		156.5		157.5		157.8
6		107.8		107.8	5.90, d (2.0)	96.4	5.91, d (2.0)	96.5
7		157.2		157.4		157.5		157.9
8	6.01, s	97.3	6.01, s	97.2	5.85, d (2.0)	95.8	5.88, d (2.0)	96.0
9		156.6		156.6		157.4		157.7
10		100.5		100.4		99.7		99.0
1′		131.3		131.3		129.9		129.7
2′	6.51, s	106.9	6.51, s	107.0		113.0		112.4
3′		146.7		146.7		146.8		146.8
4′		133.7		133.7		134.2		134.0
5′		146.7		146.7		146.7		146.8
6′	6.51, s	106.9	6.51, s	107.0	6.91, s	108.7	6.73, s	107.9
	*N*-ethyl-2-pyrrolidinone						3-*O*-Galloyl	
1″								121.5
2″		177.6		177.5			6.99, d (1.9)	106.4
3″	2.66, o, 2.42, m	32.6	2.67, o, 2.43, m	32.6				149.0
4″	2.17, m, 2.34 m	24.5	2.17, m, 2.34, m	24.5				140.6
5″	5.45, s	54.1	5.45, s	54.6				146.0
6″	2.66, o, 3.50, m	36.3	2.67, o, 3.50, m	36.3			7.03, d (1.9)	111.9
7″	1.01, t (7.2)	12.6	1.02, t (7.2)	12.6				167.8
OMe							3.81, s	56.6

s: singlet; t: triplet; m: multiplet; o: overlapped.

**Table 2:** ^1^H (600MHz) NMR data of compounds **30**, **32**, **34**, **36**, **43**, **44**, **46**, and **48**.

No.	30 ^a^	32 ^a^	34 ^a^	36 ^b^	43 ^b^	44 ^b^	46 ^a^	48 ^b^
	*δ*_H_ (J in Hz)	*δ*_H_ (J in Hz)	*δ*_H_ (J in Hz)	*δ*_H_ (J in Hz)	*δ*_H_ (J in Hz)	*δ*_H_ (J in Hz)	*δ*_H_ (J in Hz)	*δ*_H_ (J in Hz)
	Kaempferol	Kaempferol	Kaempferol	Kaempferol	Quercetin	Quercetin	Quercetin	Quercetin
6	6.19, d (2.0)	6.20, d (2.0)	6.21, d (1.9)	6.15, d (2.0)	6.11, br s	6.11, br d (8.4)	6.17, d (4.4)	6.14, dd (9.0, 2.0)
8	6.38, d (2.0)	6.38, d (2.0)	6.41, d (1.7)	6.35, d (2.1)	6.29, br s	6.29, br d (13.1)	6.36, br s	6.33, m
2′	8.00, d (8.7)	8.01, d (8.9)	8.06, d (8.9)	7.96, d (8.7)	7.46, d (2.2)	7.47, d (7.3)	7.68, d (2.2)	7.54, d (2.3)
3′	6.91, d (8.7)	6.90, d (2.1)	6.90, d (8.9)	6.88, d (8.7)				
5′	6.91, d (8.7)	6.90, d (2.1)	6.90, d (8.9)	6.88, d (8.7)	6.79, m	6.80, m	6.87, d (8.5)	6.83, m
6′	8.00, d (8.7)	8.01, d (8.9)	8.06, d (8.9)	7.96, d (8.7)	7.63, dd (8.5, 2.3)	7.63, o	7.62, dd (8.4, 2.2)	7.51, dd (5.7, 2.3)
3-*O*-	Glc	Gal	Glc	Glc	Gal	Gal	Glc	Glc
1″	5.51, d (8.0)	5.41, d (7.9)	5.17, d (7.8)	5.54, d (7.9)	5.59, d (8.0)	5.56, d (7.9)	5.13, d (7.8)	5.55, d (7.9)
2″	5.00, dd (9.6, 8.0)	5.34, m	3.65, o	4,97, m	5.22, dd (9.6, 8.0)	5.20, q (9.7)	3.27, o	5.04, m
3″	3.40, o	3.72, o	3.60, o	3.80, t (9.2)	3.73, o	3.71, o	3.60, o	3.81, q (9.0)
4″	3.35, m	3.37, m	3.39, o	3.23, m	3.72, o	3.70, o	3.38, o	3.23, o
5″	3.40, o	3.71, m	3.35, m	3.50, m	3.71, o	3.69, o	3.39, o	3.51, o
6″	3.83, d (10.5) 3.45, o	3.75, m 3.53, m	3.82, m 3.45, m	3.69, o 3.35, o	3.65, dd (10.0, 6.7), 3.30, o	3.64, t (8.2) 3.28, o	3.80, d (11.2) 3.47, o	3.71, o 3.31, o
6″-*O*-	Rha	Rha	Rha	Rha	Rha	Rha	Rha	Rha
1‴	4.55, br s	4.59, d (1.7)	4.56, d (1.7)	4.39, br s	4.49, br s	4.48, d (6.4)	4,57, br s	4.41, br d (12.1)
2‴	3.94, dd (3.3,1.7)	3.93, dd (3.3, 1.8)	3.94, dd (3.3, 1.8)	3.70, o	3.71, o	3.71, o	3.95, m	3.69, m
3‴	3.56, o	3.60, o	3.57, o	3.31, m	3.43, o	3.41, o	3.62, o	3.35, o
4‴	3.43, o	3.48, m	3.45, m	3.24, m	3.33, o	3.32, o	3.46, d (9.3)	3.25, o
5‴	3.50, m	3.59, o	3.51, dd (9.5, 6.1)	3.30, m	3.47, d (3.3)	3.45, o	3.51, m	3.33, o
6‴	1.10, d (6.0)	1.19, d (6.2)	1.11, d (6.1)	0.94, d (5.9)	1.08, d (6.1)	1.07, d (6.2)	1.12, d (6.2)	0.97, dd (9.3,6.0)
3‴-*O*-	Glc	Glc	Glc	Glc	Glc	Glc	Glc	Glc
1′′′′	4.41, d (7.7)	4.40, d (7.7)	4.40, d (7.7)	4.27, d (7.7)	4.32, d (7.7)	4.31, d (7.7)	4,43, d (7.8)	4.29, d (7.7)
2′′′′	3.28, br d (8.2)	3.25, m	3.26, m	3.02, t (8.4)	3.02, t (8.4)	3.01, t (8.4)	3.25, o	3.01, t (8.4)
3′′′′	3.40, m	3.37, o	3.39, o	3.18, d (8.7)	3.15, t (8.5)	3.15, t (8.4)	3.39, o	3.17, m
4′′′′	3.39, m	3.37, o	3.39, o	3.11, o	3.08, o	3.07, o	3.38, o	3.10, o
5′′′′	3.24, m	3.20, m	3.22, m	3.09, o	3.07, o	3.07, o	3.26, m	3.08, o
6′′′′	3.74, dd (12.1, 2.5), 3.71, dd (12.0, 4.2)	3.72, o, 3.70, o	3.72, o 3.71, o	3.56, br d (11.6) 3.43, dd (11.8, 4.5)	3.59, br d (11.6) 3.42, o	3.58, br d (11.5) 3.42, o	3.74, d (2.4) 3.72, d (4.3)	3.55, m 3.42, o
3″-*O*-			Ara	Ara			Ara	Ara
1′′′′′			4.57, d (7.2)	4.30, d (5.9)			4.34, d (7.8)	4.34, d (5.8)
2′′′′′			3.64, o	3.30, o			3.63, m	3.31, o
3′′′′′			3.65, o	3.31, o			3.58, d (3.4)	3.35, o
4′′′′′			3.82, o	3.63, br s			3.82, m	3.63, m
5′′′′′			3.92, o 3.60, o	3.73, dd (12.1, 3.6), 3.41, o			3.92, dd, (12.6, 2.8), 3.61, o	3.74, o, 3.40, o
2″-Cou	*Z*-*p*-*cou*	*Z*-*p*-*cou*		*Z*-*p*-*cou*	*E*-*p*-*cou*	*Z*-*p*-*cou*		*Z*-*p*-*cou*
2	7.64, d (8.6)	7.64, d (8.7)		7.60, d (8.5)	7.52, d (8.6)	7.64, o		7.58, m
3	6.70, d (8.5)	6.70, d (8.7)		6.63, d (8.5)	6.79, d (8.4)	6.67, d (8.4)		6.79, d (8.4)
5	6.70, d (8.5)	6.70, d (8.7)		6.63, d (8.5)	6.79, d (8.4)	6.67, d (8.4)		6.79, d (8.4)
6	7.64, d (8.6)	7.64, d (8.7)		7.60, d (8.5)	7.52, d (8.6)	7.64, o		7.58, m
7	6.88, d (12.7)	6.88, d (11.6)		6.82, d (12.9)	7.56, d (15.9)	6.82, d (12.0)		6.82, d (13.3)
8	5.89, d (12.7)	5.89, d (12.8)		5.84, d (12.8)	6.38, d (15.9)	5.81, d (12.9)		5.83, d (12.8)

^a^ Detected in methanol-*d_4_*; ^b^ Detected in DMSO-*d_6_*; s: singlet; d: dublet; t: triplet; m: multiplet; br: broad; o: overlapped.

**Table 3:** ^13^C (150MHz) NMR data of compounds **30**, **32**, **34**, **36**, **43**, **44**, **46**, and **48**.

No.	30 ^a^	32 ^a^	34 ^a^	36 ^b^	43 ^b^	44 ^b^	46 ^a^	48 ^b^
2	158.9	158.6	159.2	156.8	156.1	156.1	158.9	156.8
3	134.6	134.9	135.5	132.7	132.8	132.9	135.5	132.8
4	179.1	179.3	179.2	176.9	176.8	176.9	179.0	176.9
5	163.1	163.1	163.0	161.1	161.2	161.2	162.8	161.2
6	100.0	100.0	100.4	98.9	99.1	99.1	101.0	98.8
7	166.1	166.0	167.1	165.0	165.6	165.5	169.0	164.9
8	95.0	94.9	95.3	94.0	93.8	93.8	95.7	93.8
9	158.5	158.5	158.7	156.6	156.4	156.4	158.8	156.5
10	105.8	105.7	105.6	103.7	103.4	103.4	105.1	103.8
1′	122.9	122.7	122.8	120.7	120.7	120.8	123.1	120.8
2′	132.4	132.3	132.5	130.9	115.7	115.7	117.7	116.0
3′	116.3	116.3	116.2	115.2	145.0	145.0	145.9	144.8
4′	161.3	161.5	161.5	160.0	148.9	148.9	149.9	148.7
5′	116.3	116.3	116.2	115.2	115.2	115.2	116.1	115.2
6′	132.4	132.3	132.5	130.9	122.1	122.2	123.4	121.7
3-*O*-	Glc	Gal	Glc	Glc	Gal	Gal	Glc	Glc
1″	100.7	101.4	104.3	98.9	99.2	99.2	104.5	99.0
2″	75.4	73.8	74.2	72.5	72.4	72.3	75.2	72.5
3″	76.0	73.1	86.9	80.5	71.0	70.8	87.0	80.6
4″	71.7	70.9	76.8	68.9	68.1	68.1	69.8	68.9
5″	77.2	75.4	70.0	75.1	73.2	73.2	76.8	75.3
6″	68.2	67.4	68.8	67.4	64.9	65.0	68.7	67.8
6″-*O*-	Rha	Rha	Rha	Rha	Rha	Rha	Rha	Rha
1‴	102.1	101.8	102.4	101.1	100.1	100.1	102.4	101.1
2‴	71.3	71.3	71.3	69.2	69.4	69.4	71.3	69.2
3‴	83.2	83.0	83.2	81.8	81.7	81.7	83.1	81.6
4‴	72.5	72.6	72.6	70.2	70.9	70.9	72.6	70.2
5‴	69.4	69.5	69.5	67.8	67.8	67.9	69.4	67.8
6‴	17.9	18.1	18.0	17.6	17.9	17.9	18.0	17.6
3‴-*O*-	Glc	Glc	Glc	Glc	Glc	Glc	Glc	Glc
1′′′′	105.7	105.7	105.7	104.4	104.5	104.4	105.6	104.3
2′′′′	75.5	75.4	75.5	74.1	74.1	74.1	75.5	74.1
3′′′′	77.6	77.6	77.5	76.0	76.1	76.1	77.5	76.0
4′′′′	70.9	70.5	70.8	69.6	69.7	69.7	70.9	69.6
5′′′′	77.6	77.6	77.6	76.4	76.6	76.6	77.6	76.4
6′′′′	62.1	62.1	62.1	60.7	60.8	60.9	62.1	60.7
3″-*O*-			Ara	Ara			Ara	Ara
1′′′′′			105.4	103.1			105.6	103.1
2′′′′′			73.0	70.6			73.0	70.7
3′′′′′			75.2	72.4			74.2	72.4
4′′′′′			69.7	67.2			69.7	67.2
5′′′′′			67.3	65.3			67.3	65.2
2″-*Cou*	*Z*-*p*-*cou*	*Z*-*p*-*cou*		*Z*-*p*-*cou*	*E*-*p*-*cou*	*Z*-*p*-*cou*		*Z*-*p*-*cou*
1	127.7	127.7		125.5	125.2	125.5		125.5
2	133.7	133.7		132.4	130.2	132.6		132.4
3	115.8	115.8		114.7	115.8	114.9		116.2
4	160.0	159.9		158.6	159.8	158.7		158.6
5	115.8	115.8		114.7	115.8	114.9		116.2
6	133.7	133.7		132.4	130.2	132.6		132.4
7	144.8	144.8		142.6	144.7	142.6		142.6
8	116.9	117.0		116.0	114.6	115.9		114.7
9	167.6	167.8		165.0	166.0	166.1		164.9

^a^ Detected in methanol-*d*_4_; ^b^ Detected in DMSO-*d*_6_.

## Data Availability

The experimental data presented in the current study are included in the article and Supplementary Materials.

## Supplementary Materials

The following supporting information can be downloaded at: https://www.mdpi.com/article/10.3390/molecules30051024/s1, Experimental methods: refers to the detailed procedure of isolation and acid hydrolysis experiment; Table S1. Docking results of four compounds with SARS-CoV-2 3CLpro receptor; Table S2. The identification results of isolated compounds from RGWRT by LC-MS; Figures S1–S81: HR-ESI-MS and NMR spectra of new compounds **11**, **12**, **20**, **30**, **32**, **34**, **36**, **43**, **44**, **46**, and **48**; Figure S82: Circular dichroism (CD) spectra of **11** and **12**.
