# Supplementary material for: Diverse Structures of Tea Polyphenols from Rougui Wuyi Rock Tea and Their Potential as Inhibitor of 3C-like Protease
Source: Molecules. 2025 Feb 23;30(5):1024. doi: 10.3390/molecules30051024 (PMC11901911; doi:10.3390/molecules30051024)
Supplement: Supplementary file 1 [file molecules-30-01024-s001.zip › molecules-3461758-supplementary.pdf]

# List of the contents


**1. Experimental methods**

**2. Experimental data**

**Table S1.** Docking results of four compounds with SARS-CoV-2 3CLpro receptor.

**Table S2.** The identification results of isolated compounds from RGWRT by LC-MS.

**Figure S1.** HR-ESI-MS data of compound **11** in the negative mode.

**Figure S2.** $^{1}$H NMR data of compound **11** in methanol-$d_4$.

**Figure S3.** $^{13}$C NMR data of compound **11** in methanol-$d_4$.

**Figure S4.** DEPT data of compound **11** in methanol-$d_4$.

**Figure S5.** COSY data of compound **11** in methanol-$d_4$.

**Figure S6.** HSQC data of compound **11** in methanol-$d_4$.

**Figure S7.** HMBC data of compound **11** in methanol-$d_4$.

**Figure S8.** ROESY data of compound **11** in DMSO-$d_4$.

**Figure S9.** NOESY data of compound **11** in methanol-$d_4$.

**Figure S10.** HR-ESI-MS data of compound **12** in the negative mode.

**Figure S11.** $^{1}$H NMR data of compound **12** in methanol-$d_4$.

**Figure S12.** $^{13}$C NMR data of compound **12** in methanol-$d_4$.

**Figure S13.** DEPT data of compound **12** in methanol-$d_4$.

**Figure S14.** COSY data of compound **12** in methanol-$d_4$.

**Figure S15.** HSQC data of compound **12** in methanol-$d_4$.

**Figure S16.** HMBC data of compound **12** in methanol-$d_4$.

**Figure S17.** ROESY data of compound **12** in DMSO-$d_6$.

**Figure S18.** NOESY data of compound **12** in methanol-*d*4.

**Figure S19.** HR-ESI-MS data of compound **20** in the negative mode.

**Figure S20.** $^{1}$H NMR data of compound **20** in methanol-*d*4.

**Figure S21.** $^{13}$C NMR data of compound **20** in methanol-*d*4.

**Figure S22.** COSY data of compound **20** in methanol-*d*4.

**Figure S23.** HSQC data of compound **20** in methanol-*d*4.

**Figure S24.** HMBC data of compound **20** in methanol-*d*4.

**Figure S25.** NOESY data of compound **20** in methanol-*d*4.

**Figure S26.** HR-ESI-MS data of compound **30** in the negative mode.

**Figure S27.** $^{1}$H NMR data of compound **30** in methanol-*d*4.

**Figure S28.** $^{13}$C NMR data of compound **30** in methanol-*d*4.

**Figure S29.** COSY data of compound **30** in methanol-*d*4.

**Figure S30.** HSQC data of compound **30** in methanol-*d*4.

**Figure S31.** HMBC data of compound **30** in methanol-*d*4.

**Figure S32.** NOESY data of compound **30** in methanol-*d*4.

**Figure S33.** HR-ESI-MS data of compound **32** in the negative mode.

**Figure S34.** $^{1}$H NMR data of compound **32** in methanol-*d*4.

**Figure S35.** $^{13}$C NMR data of compound **32** in methanol-*d*4.

**Figure S36.** COSY data of compound **32** in methanol-*d*4.

**Figure S37.** HSQC data of compound **32** in methanol-*d*4.

**Figure S38.** HMBC data of compound **32** in methanol-*d*4.

**Figure S39.** NOESY data of compound **32** in methanol-*d*4.

**Figure S40.** HR-ESI-MS data of compound **34** in the negative mode.

**Figure S41.** $^{1}$H NMR data of compound **34** in methanol-$d_4$.

**Figure S42.** $^{13}$C NMR data of compound **34** in methanol-$d_4$.

**Figure S43.** COSY data of compound **34** in methanol-$d_4$.

**Figure S44.** HSQC data of compound **34** in methanol-$d_4$.

**Figure S45.** HMBC data of compound **34** in methanol-$d_4$.

**Figure S46.** NOESY data of compound **34** in methanol-$d_4$.

**Figure S47.** HR-ESI-MS data of compound **36** in the negative mode.

**Figure S48.** $^{1}$H NMR data of compound **36** in DMSO-$d_6$.

**Figure S49.** $^{13}$C NMR data of compound **36** in DMSO-$d_6$.

**Figure S50.** COSY data of compound **36** in DMSO-$d_6$.

**Figure S51.** HSQC data of compound **36** in DMSO-$d_6$.

**Figure S52.** HMBC data of compound **36** in DMSO-$d_6$.

**Figure S53.** NOESY data of compound **36** in DMSO-$d_6$.

**Figure S54.** HR-ESI-MS data of compound **43** in the negative mode.

**Figure S55.** $^{1}$H NMR data of compound **43** in DMSO-$d_6$.

**Figure S56.** $^{13}$C NMR data of compound **43** in DMSO-$d_6$.

**Figure S57.** COSY data of compound **43** in DMSO-$d_6$.

**Figure S58.** HSQC data of compound **43** in DMSO-$d_6$.

**Figure S59** HMBC data of compound **43** in DMSO-$d_6$.

**Figure S60.** NOESY data of compound **43** in DMSO-$d_6$.

**Figure S61.** HR-ESI-MS data of compound **44** in the negative mode.

**Figure S62.** $^{1}H$ NMR data of compound **44** in DMSO-$d_6$.

**Figure S63.** $^{13}C$ NMR data of compound **44** in DMSO-$d_6$.

**Figure S64.** COSY data of compound **44** in DMSO-$d_6$.

**Figure S65.** HSQC data of compound **44** in DMSO-$d_6$.

**Figure S66.** HMBC data of compound **44** in DMSO-$d_6$.

**Figure S67.** NOESY data of compound **44** in DMSO-$d_6$.

**Figure S68.** HR-ESI-MS data of compound **46** in the negative mode.

**Figure S69.** $^{1}H$ NMR data of compound **46** in methanol-$d_4$.

**Figure S70.** $^{13}C$ NMR data of compound **46** in methanol-$d_4$.

**Figure S71.** COSY data of compound **46** in methanol-$d_4$.

**Figure S72.** HSQC data of compound **46** in methanol-$d_4$.

**Figure S73.** HMBC data of compound **46** in methanol-$d_4$.

**Figure S74.** NOESY data of compound **46** in methanol-$d_4$.

**Figure S75.** HR-ESI-MS data of compound **48** in the negative mode.

**Figure S76.** $^{1}H$ NMR data of compound **48** in DMSO-$d_6$.

**Figure S77.** $^{13}C$ NMR data of compound **48** in DMSO-$d_6$.

**Figure S78.** COSY data of compound **48** in DMSO-$d_6$.

**Figure S79.** HSQC data of compound **48** in DMSO-$d_6$.

**Figure S80.** HMBC data of compound **48** in DMSO-$d_6$.

**Figure S81.** NOESY data of compound **48** in DMSO-$d_6$.

**Figure S82.** Circular dichroism (CD) spectra of **11** and **12.**

## 1. Experimental methods

The detailed procedure of isolation and acid hydrolysis experiment was as follows:

After dissolving in deionized water, the supernatant of Fr. B (82.6 g) was separated on a macroporous resin SP825 column eluting with EtOH−$H_2O$ (0:100, 10:90, 30:70, 50:50, 70:30 and 95:5, v/v) to yield six factions (B1–B6). Fr. B4 was performed on preparative MPLC coupled with a Flash Pure column (FP ECOFLEX C18 220 g, MeOH−$H_2O$, 20:80, 15:85, v/v, 70 mL/min) to produce fourteen fractions (B4-a−B4-n). Fr. B4-d was purified by Welch Ultimate XB-C18 column with MeCN−$H_2O$ (20:80, v/v, 4 mL/min), followed by Lux i-cellulose column with MeCN−$H_2O$ (18:82, v/v, 4 mL/min), to afford compounds **7** (17.4 mg, $t_R$ 17.8 min) and **3** (29.6 mg, $t_R$ 19.3 min). Compounds **1** (5.7 mg, $t_R$ 5.5 min) and **49** (9 mg, $t_R$ 4.8 min) were separated from Fr. B4-c by repeated Welch Ultimate XB-C18 column with MeCN−$H_2O$ (15:85, v/v, 4 mL/min). On the other hand, the precipitation of Fr. B (82.6 g) was subjected to silica gel CC eluting with $CH_2Cl_2$−MeOH (100:0, 200:1, 150:1, 50:1, 30:1, 15:1, 10:1, 5:1, 3:1 and 0:100, v/v) to give twenty fractions (B7–B26). After merging Fr. B17 into Fr. B16, Fr. B16 (3.9 g) was fractioned by preparative MPLC equipped with a Flash Pure column (FP ECOFLEX C18 220 g, MeCN−$H_2O$, 10:90, 85:15, v/v, 100 mL/min) to obtain twenty-six fractions (Fr. B16 -a − Fr. B16 -z). Compound **22** (17.3 mg, $t_R$ 13.5 min) was separated from Fr. B16 -j by semi-preparative HPLC with MeOH−0.1% FA+$H_2O$ (50:50, v/v, 4 mL/min). Fr. B22 (16.9 g) was fractioned via preparative MPLC coupled with a Flash Pure column

(FP ECOFLEX C18 220 g, MeCN−$H_2O$, 10:90, 30:70, 50:50, 70:30, 98:2, v/v, 100 mL/min) to obtain thirteen fractions (B22-a−B22-m). Fr. B22-a was separated into seven fractions (B22-a1−B22-a7) and to afford compound **2** (49.7 mg, $t_R$ 11.5 min) by preparative HPLC using a mobile phase of MeCN−$H_2O$ (20:80, v/v, 4 mL/min). After merging Fr. B22-a4 into Fr. B22-a3, Fr. B22-a3 was further purified through semi-preparative HPLC using a mobile phase of MeCN−$H_2O$ (8:92, v/v, 4 mL/min) to give compound **5** (277.8 mg, $t_R$ 6.3 min). Fr. B22-a5 was purified via semi-preparative HPLC using a mobile phase of MeCN−$H_2O$ (15:85, v/v, 4 mL/min) to obtain compound **6** (23.2 mg, $t_R$ 10.4 min).

Fr. C (220 g) was separated on a macroporous resin SP825 column eluting with EtOH−$H_2O$ (0:100, 10:90, 30:70, 50:50, 70:30 and 95:5, v/v) to yield six factions (C1–C6). Fr. C3 (99.5 g) was chromatographed over silica gel CC eluting with $CH_2Cl_2$−MeOH−$H_2O$ saturation (lower layer) (20:1, 10:1, 5:1, 4:1, 3:1, 3:1.3, 2:1 and 0:100, v/v) to give twelve fractions (C3-a–C3-l). Fr. C3-e (2 g) was fractioned by preparative MPLC equipped with a Flash Pure column (FP ECOFLEX C18 220 g, MeOH−$H_2O$, 20:80, 60:40, 100:0, v/v, 50 mL/min) to obtain thirteen fractions (C3-e1–C3-e13). Fr. C3-e3 was subjected to the ChromCore Phenyl column using MeCN−$H_2O$ (15:85, v/v, 2.8 mL/min) to yield compound **8** (27.4 mg, $t_R$ 14.8 min). Fr. C3-e6 was further purified through the ChromCore Phenyl column with MeCN−$H_2O$ (25:75, v/v, 2.8 mL/min), followed by ChromCore Phenyl with MeCN−$H_2O$ (15:85, v/v, 2.8 mL/min), to afford compound **13** (6.9 mg, $t_R$ 43.2 min). Fr. C3-f (6 g) was fractioned by preparative MPLC equipped with a Flash Pure column (FP ECOFLEX

C18 800 g, MeOH−$H_2O$, 20:80, 60:40, 100:0, v/v, 50 mL/min) to obtain thirteen fractions (C3-f1–C3-f13). Compound **4** (71.3 mg, $t_R$ 21 min) was separated from Fr. C3-f5 by repeated ChromCore Phenyl column with MeCN−$H_2O$ (10:90, v/v, 2.8 mL/min). Fr. C3-f9 was further purified by Welch Ultimate XB-C18 column with MeCN−$H_2O$ (25:75, v/v, 2.8 mL/min), followed by Silgreen C18 column with MeCN−$H_2O$ (18:82, v/v, 2.8 mL/min), to afford compound **38** (39.9 mg, $t_R$ 33.5 min) and **37** (21.6 mg, $t_R$ 37.5 min). Fr. C3-f7 (1.8 g) was further separated on Flash Pure column (FP ECOFLEX C18 220 g, MeOH−$H_2O$, 20:80, 35:65, 70:30, 100:0, v/v, 50 mL/min), followed by Silgreen C18 column with MeCN−$H_2O$ (18:82, v/v, 2.8 mL/min), and finally elute with MeCN−$H_2O$ (10:90, v/v, 2.8 mL/min) on Silgreen C18 to obtain compounds **14** (10.3 mg, $t_R$ 36 min), **11*** (5.4 mg, $t_R$ 46.5 min), **12*** (6.1 mg, $t_R$ 48.5 min) and **15** (28.5 mg, $t_R$ 52.5 min). Compound **24** (20.3 mg, $t_R$ 19.2 min) and **23** (13 mg, $t_R$ 21.5 min) were separated from Fr. C3-f10 via Silgreen C18 column with MeCN−$H_2O$ (18:82, v/v, 2.8 mL/min). Fr. C3-i (5.8 g) was fractioned by preparative MPLC equipped with a Flash Pure column (FP ECOFLEX C18 800 g, MeOH−$H_2O$, 20:80, 30:70, 40:60, 50:50, 60:40, 100:0, v/v, 60 mL/min) to obtain seventeen fractions (C3-i1–C3-i17). Fr. C3-i12 was fractioned by Silgreen C18 column with MeCN−$H_2O$ (18:82, v/v, 12.7 mL/min) to give compounds **28** (290.9 mg, $t_R$ 9.5 min) and **27** (522.8 mg, $t_R$ 11.3 min). Fr. C3-i11 was subjected to Silgreen C18 column eluting with MeCN−$H_2O$ (15:75, v/v, 4.7 mL/min) to obtain compounds **40** (8.1 mg, $t_R$ 22.1 min) and **54** (9.3 mg, $t_R$ 27.5 min). Compound **53** (16.9 mg, $t_R$ 14.8 min) was separated from Fr. C3-i11 by Silgreen C18 column eluting with MeCN−$H_2O$

(15:75, v/v, 4.7 mL/min), then ChromCore Phenyl column with MeOH−$H_2O$ (30:70, v/v, 2.8 mL/min). Fr. C3-i10 was then purified by the Silgreen C18 column with MeCN−$H_2O$ (15:85, v/v, 4.7 mL/min), followed by ChromCore Phenyl with MeCN−$H_2O$ (10:90, v/v, 2.8 mL/min) and Lux i-cellulose column with MeCN−$H_2O$ (10:90, v/v, 4 mL/min), to give compounds **56** (27.6 mg, $t_R$ 38.5 min), **57** (22.2 mg, $t_R$ 42.8 min), and **59** (4.7 mg, $t_R$ 10.3 min). Fr. C3-i8 was fractioned by the Silgreen C18 column with MeCN−$H_2O$ (13:87, v/v, 12.7 mL/min) to obtain compound **9** (50.1 mg, $t_R$ 14.2 min). Compound **16** (39.9 mg, $t_R$ 14.1 min) was separated from Fr. C3-i8 by the Silgreen C18 column with MeCN−$H_2O$ (11:89, v/v, 4.7 mL/min).

Fr. C3-j (5.4 g) was fractioned by preparative MPLC equipped with a Flash Pure column (FP ECOFLEX C18 800 g, MeOH−$H_2O$, 20:80, 25:75, 30:70, 40:60, 50:50, 60:40,70:30, 100:0, v/v, 65 mL/min) to obtain seventeen fractions (C3-j1–C3-j17). Compound **34*** (5.8 mg, $t_R$ 35 min) was isolated from Fr. C3-j14 (merging Fr. C3-j15 into Fr. C3-j14) using the Silgreen C18 column with MeCN−$H_2O$ (15:85, v/v, 4.7 mL/min). Fr. C3-j13 was refined via ChromCore Phenyl with MeCN−$H_2O$ (12:88, v/v, 2.8 mL/min) to yield compounds **41** (65 mg, $t_R$ 25 min), and **46*** (8.1 mg, $t_R$ 41.3 min). Fr. C3-j12 was similarly processed by ChromCore Phenyl with MeCN−$H_2O$ (12:88, v/v, 2.8 mL/min) to yield compound **51** (29.6 mg, $t_R$ 22.8 min). Fr. C3-j7 was purified using Silgreen C18 column with MeCN−$H_2O$ (11:89, v/v, 4.7 mL/min), isolating compounds **20*** (1.4 mg, $t_R$ 11.5 min), **18** (17 mg, $t_R$ 23.5 min), and **17** (8.5 mg, $t_R$ 27.2 min). Fr. C3-j6 was fractioned by Silgreen C18 column with MeCN−$H_2O$ (11:89, v/v, 4.7 mL/min) to give compounds **21** (57.8 mg, $t_R$ 11.4 min), and **19** (8.3 mg, $t_R$

12.7 min). Fr. C3-d (2.3 g) was fractioned by preparative MPLC equipped with a Flash Pure column (FP ECOFLEX C18 220 g, MeOH−$H_2O$, 30:70, 35:65, 40:60, 45:55, 50:50, 55:45, 60:40, 70:30, 100:0, v/v, 60 mL/min) to obtain fifteen fractions (C3-d1–C3-d15). After merging Fr. C3-d11 into Fr. C3-d10, Fr. C3-d10 was processed through Silgreen C18 column with MeCN−$H_2O$ (24:76, v/v, 4.7 mL/min) to separate compounds **45** (2.1 mg, $t_R$ 17.5 min), **31** (2.5 mg, $t_R$ 21.2 min), **29** (7.2 mg, $t_R$ 22.8 min), **33** (1.9 mg, $t_R$ 26 min), **32*** (1.8 mg, $t_R$ 28 min), and **30*** (3.6 mg, $t_R$ 34.8 min). Compounds **25** (2.4 mg, $t_R$ 28.7 min) and **55** (92.7 mg, $t_R$ 30.3 min) were separated from Fr. C3-d6 (merging Fr. C3-d7 into Fr. C3-d6) by the Silgreen C18 column with MeCN−$H_2O$ (18:82, v/v, 4.7 mL/min). Fr. C3-d5 was fractioned by the Silgreen C18 column with MeCN−$H_2O$ (15:85, v/v, 4.7 mL/min) to give compound **26** (8 mg, $t_R$ 17 min). Fr. C3-d4 was further purified by ChromCore Phenyl column with MeCN−$H_2O$ (15:85, v/v, 2.8 mL/min) to afford compound **10** (2.8 mg, $t_R$ 17.4 min), followed by Silgreen C18 column with MeCN−$H_2O$ (12:88, v/v, 4.7 mL/min) to give compound **39** (19.6 mg, $t_R$ 60 min), and on the same Silgreen C18 with MeCN−$H_2O$ (11:89, v/v, 4.7 mL/min) to obtain compounds **58** (1.2 mg, $t_R$ 70 min) and **52** (6.9 mg, $t_R$ 82.5 min). Fr. C3-e (4.5 g) was fractioned by preparative MPLC equipped with a Flash Pure column (FP ECOFLEX C18 800 g, MeOH−$H_2O$, 30:70, 35:65, 40:60, 45:55, 50:50, 55:45, 60:40, 70:30, 100:0, v/v, 70 mL/min) to obtain twenty-two fractions (C3-e1–C3-e22). Compounds **35** (116.9 mg, $t_R$ 27.1 min) and **36*** (32.4 mg, $t_R$ 33.5 min) were isolated from Fr. C3-e15 by the Silgreen C18 column with MeCN−$H_2O$ (23:77, v/v, 4.7 mL/min). Fr. C3-e14 was similarly chromatographed by the Silgreen

C18 column with MeCN−$H_2O$ (23:77, v/v, 4.7 mL/min) to yield compounds **47** (113.6 mg, $t_R$ 19 min) and **42** (47.9 mg, $t_R$ 20 min), followed by ChromCore Phenyl column with MeCN−$H_2O$ (21:79, v/v, 2.8 mL/min) to give compounds **43** (5.8 mg, $t_R$ 23.5 min), **44** (1.8 mg, $t_R$ 32.5 min),**48** (5.1 mg, $t_R$ 34.6 min). Compound **50** (1.8 mg, $t_R$ 23.5 min) was separated from Fr. C3-e8 by the Silgreen C18 column with MeCN−$H_2O$ (13:87, v/v, 4.7 mL/min).

Compound **30** (1 mg) was dissolved in 6 M $CF_3COOH$ (1 mL) and heated at 90 ˚C for 2 h and cooled to room temperature. Then, the reaction solution was extracted three times with an appropriate amount of trichloromethane, and the top water layer was dried with nitrogen. The dried residues of compound **30** and standard sugars (D-glucose, D-galactose, L-rhamnose and L-arabinose) were dissolved in anhydrous pyridine (200 μL), and mixed with L-cysteine methyl ester hydrochloride (1 mg) and warmed at 60 ˚C water bath for 1 h, respectively. Subsequently, O-tolyl isothiocyanate (10 μL) was added into the reaction solution and heated in 60 ˚C water bath for another 1 h. After the reaction, the final products were dried with nitrogen. Then, those dissolved in HPLC grade methanol (1 mL) and passed through 0.22 microporous filter membrane. Next, the final products analyzed by UHPLC-CAD using a Waters HSS T3 column (1.8 μM, 100 mm × 2.1 mm). The mobile phases system consists of water containing 0.1 % formic acid (A) and acetonitrile (B) at a flow rate of 0.6 mL/min. The elution program was eluted from 20 % to 30 %B for 8 min, 0.5 min up to 95 % B, 95 % B for 3 min, 0.5 min down to 20 % B, and then 20% B for 3.5 min. The derivative of D-glucose, D-galactose, L-rhamnose and L-arabinose

was detected at retention time 4.7 min, 4.3 min, 6.7 min, and 5.1 min, respectively. For compound **30**, D-glucose and L-rhamnose were observed by comparing retention time with the standard sugars. Similarly, the configuration of monosaccharides in compounds **32**, **34**, **36**, **43**, **44**, **46**, **48** were determined by referring to compound **30**.

## 2. Experimental data

**Table S1.** Docking results of four compounds with SARS-CoV-2 3CLpro receptor.

| No. | Affinity (kcal/mol) | Estimated Ki (μM) | No of H-bonds | Amino acid residues forming H-bond |
|---|---|---|---|---|
| **5** | -7.5 | 3.18 | 5 | THR24, THR25, THR26, THR45, ASN119 |
| **6** | -7.0 | 7.4 | 4 | ASN142, ASN119, THR25, HIS41 |
| **11** | -6.8 | 10.37 | 1 | ASN142 |
| **15** | -6.1 | 33.78 | 2 | LEU4, GLY143 |

**Table S2.** The identification results of isolated compounds from RGWRT by LC-MS.

| Compound | RT (min) | Mass (*m/z*) | Mass Error (PPM) | Adduct | Formula | MS/MS |
|---|---|---|---|---|---|---|
| **4** | 5.23 | 305.0700 | -2.3 | $[M-H]^-$ | $C_{15}H_{14}O_7$ | 179.0341, 165.0184, 125.0237 |
| **8** | 5.61 | 289.0712 | 0.0 | $[M-H]^-$ | $C_{15}H_{14}O_6$ | 179.0347, 165.0191, 125.0236 |
| **21** | 6.56 | 913.1499 | 3.9 | $[M-H]^-$ | $C_{44}H_{34}O_{22}$ | 591.1140, 407.0758, 289.0702 |
| **16** | 6.92 | 577.1368 | 3.8 | $[M-H]^-$ | $C_{30}H_{26}O_{12}$ | 407.0757, 305.0668, 289.0706, 125.0238 |
| **1** | 7.59 | 289.0706 | -2.1 | $[M-H]^-$ | $C_{15}H_{14}O_6$ | 179.0339, 165.0182, 125.0238 |
| **5** | 7.73 | 457.0823 | 11.38 | $[M-H]^-$ | $C_{22}H_{18}O_{11}$ | 331.0450, 305.0656, 169.0136, 125.0238 |
| **14** | 8.45 | 416.1357 | 2.9 | $[M-H]^-$ | $C_{21}H_{23}NO_8$ | 305.0660, 221.0450, 169.0132, 125.0236 |
| **9** | 8.67 | 457.0796 | 5.5 | $[M-H]^-$ | $C_{22}H_{18}O_{11}$ | 305.0652, 169.0136, 125.0237 |
| **11** | 9.07 | 416.1354 | 2.2 | $[M-H]^-$ | $C_{21}H_{23}NO_8$ | 305.0656, 289.0701, 169.0128, 125.0235 |
| **12** | 9.17 | 416.1352 | 1.7 | $[M-H]^-$ | $C_{21}H_{23}NO_8$ | 305.0639, 191.0548, 169.0133, 125.0237 |
| **15** | 9.46 | 416.1357 | 2.9 | $[M-H]^-$ | $C_{21}H_{23}NO_8$ | 305.0638, 169.0139, 125.0235 |
| **50** | 9.98 | 625.1429 | 3.8 | $[M-H]^-$ | $C_{27}H_{30}O_{17}$ | 316.0210, 317.0268, 301.0348 |
| **49** | 10.11 | 479.0847 | 4.4 | $[M-H]^-$ | $C_{21}H_{20}O_{13}$ | 316.0211, 317.0262, 271.0234 |
| **6** | 10.43 | 471.0959 | 6.8 | $[M-H]^-$ | $C_{23}H_{20}O_{11}$ | 305.0655, 183.0291, 139.0394,125.0238 |
| **57** | 10.70 | 563.1406 | 6.2 | $[M-H]^-$ | $C_{26}H_{28}O_{14}$ | 431.0966, 269.0423 |
| **41** | 11.19 | 771.1985 | 3.5 | $[M-H]^-$ | $C_{33}H_{40}O_{21}$ | 609.1464, 301.0336 |
| **2** | 11.59 | 441.0871 | 11.1 | $[M-H]^-$ | $C_{22}H_{18}O_{10}$ | 289.0706, 169.0136, 125.0237 |
| **40** | 12.17 | 771.2015 | 4.0 | $[M-H]^-$ | $C_{33}H_{40}O_{21}$ | 609.1411, 301.0332 |
| **38** | 12.82 | 463.0895 | 3.9 | $[M-H]^-$ | $C_{21}H_{20}O_{12}$ | 301.0328 |
| **39** | 13.25 | 609.1475 | 3.1 | $[M-H]^-$ | $C_{27}H_{30}O_{16}$ | 577.1550, 301.0332, 125.0237 |
| **28** | 13.47 | 755.2073 | 5.0 | $[M-H]^-$ | $C_{33}H_{40}O_{20}$ | 593.1489, 285.0391 |
| **37** | 13.59 | 463.0893 | 3.5 | $[M-H]^-$ | $C_{21}H_{20}O_{12}$ | 301.0330 |
| **53** | 13.96 | 577.1590 | 5.7 | $[M-H]^-$ | $C_{27}H_{30}O_{14}$ | 431.0960, 413.0865, 269.0461 |
| **26** | 14.99 | 593.1506 | 3.0 | $[M-H]^-$ | $C_{27}H_{30}O_{15}$ | 285.0391 |
| **27** | 15.30 | 755.2112 | 10.2 | $[M-H]^-$ | $C_{33}H_{40}O_{20}$ | 593.1513, 285.0394 |
| **24** | 15.40 | 447.0945 | 4.0 | $[M-H]^-$ | $C_{21}H_{20}O_{11}$ | 285.0383 |

| | | | | | | |
|---|---|---|---|---|---|---|
| **7** | 15.60 | 425.0887 | 3.3 | [M-H]$^-$ | $C_{22}H_{18}O_9$ | 273.0759, 255.0655, 169.0135, 125.0238 |
| **3** | 15.84 | 455.0991 | 2.9 | [M-H]$^-$ | $C_{23}H_{20}O_{10}$ | 289.0706, 183.0291, 125.0235 |
| **25** | 15.93 | 593.1506 | 5.1 | [M-H]$^-$ | $C_{27}H_{30}O_{15}$ | 285.0392 |
| **23** | 16.08 | 447.0941 | 3.1 | [M-H]$^-$ | $C_{21}H_{20}O_{11}$ | 285.0377 |
| **47/48** | 17.94 | 1049.2837 | 6.0 | [M-H]$^-$ | $C_{47}H_{54}O_{27}$ | 917.2332, 771.2001, 609.1295, 301.0336 |
| **42** | 18.04 | 917.2385 | 3.6 | [M-H]$^-$ | $C_{42}H_{46}O_{2}3$ | 771.2015, 609.1332, 301.0345 |
| **35/36** | 18.30 | 1033.2924 | 9.6 | [M-H]$^-$ | $C_{47}H_{54}O_{26}$ | 887.2410, 755.1830, 285.0393 |
| **29** | 18.45 | 901.2410 | 2.1 | [M-H]$^-$ | $C_{42}H_{46}O_{22}$ | 755.2123, 463.0926, 285.0386 |

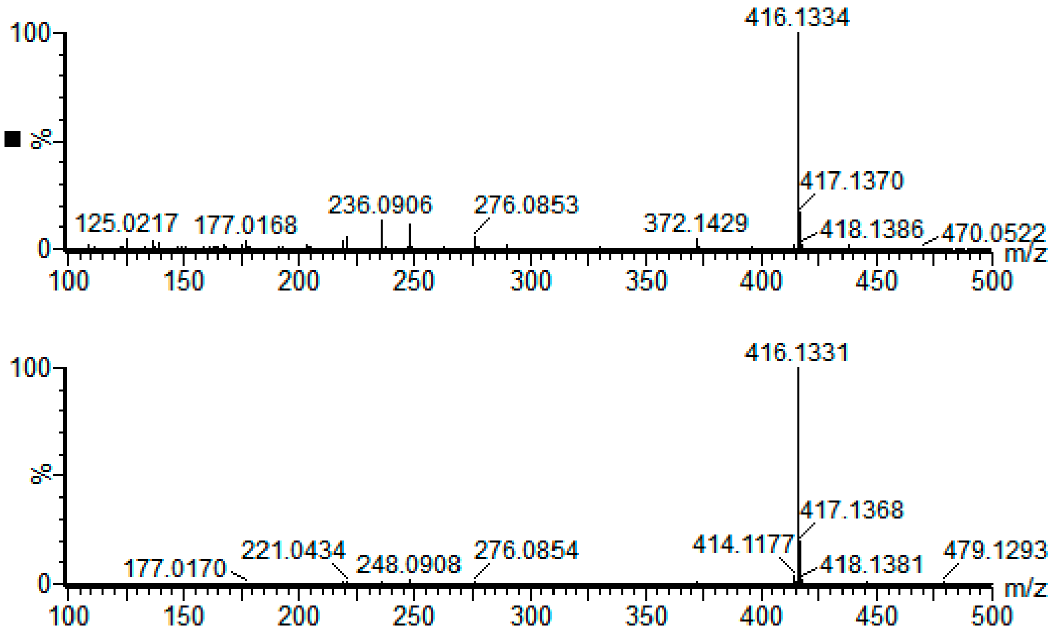


**Figure S1.** HR-ESI-MS data of compound **11** in the negative mode.

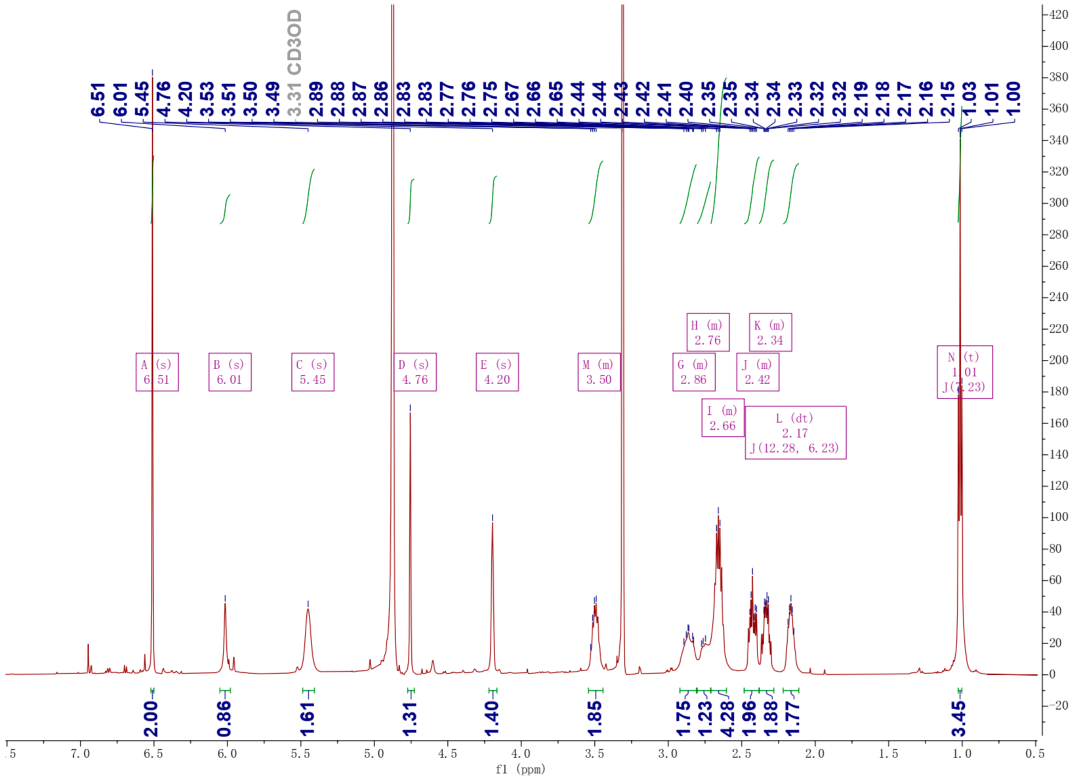


**Figure S2.** $^{1}$H NMR data of compound **11** in methanol-$d_4$.

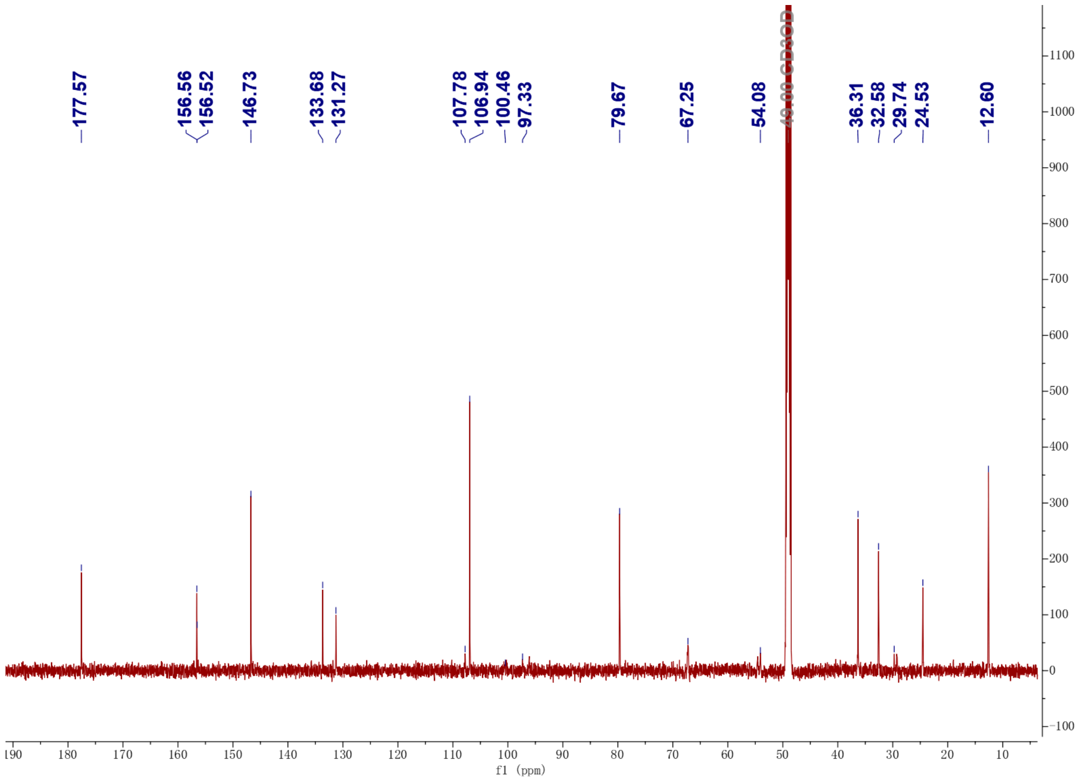


**Figure S3.** $^{13}C$ NMR data of compound **11** in methanol-$d_4$.

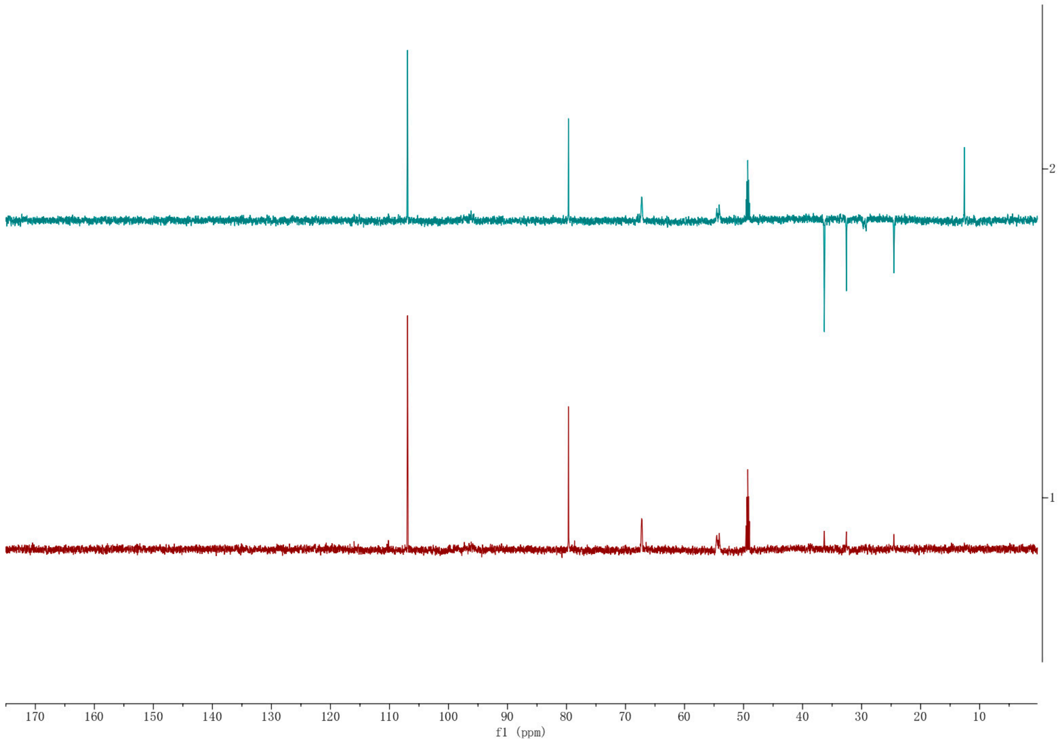


**Figure S4.** DEPT data of compound **11** in methanol-$d_4$.

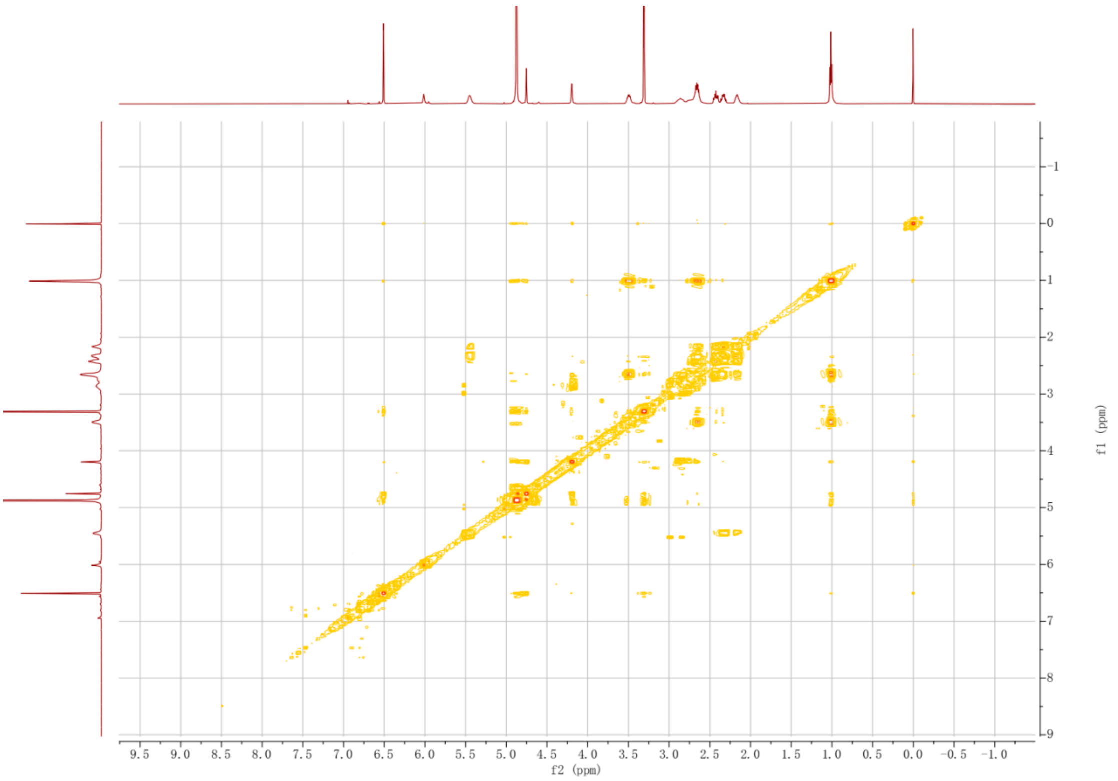


**Figure S5.** COSY data of compound **11** in methanol-*d*4.

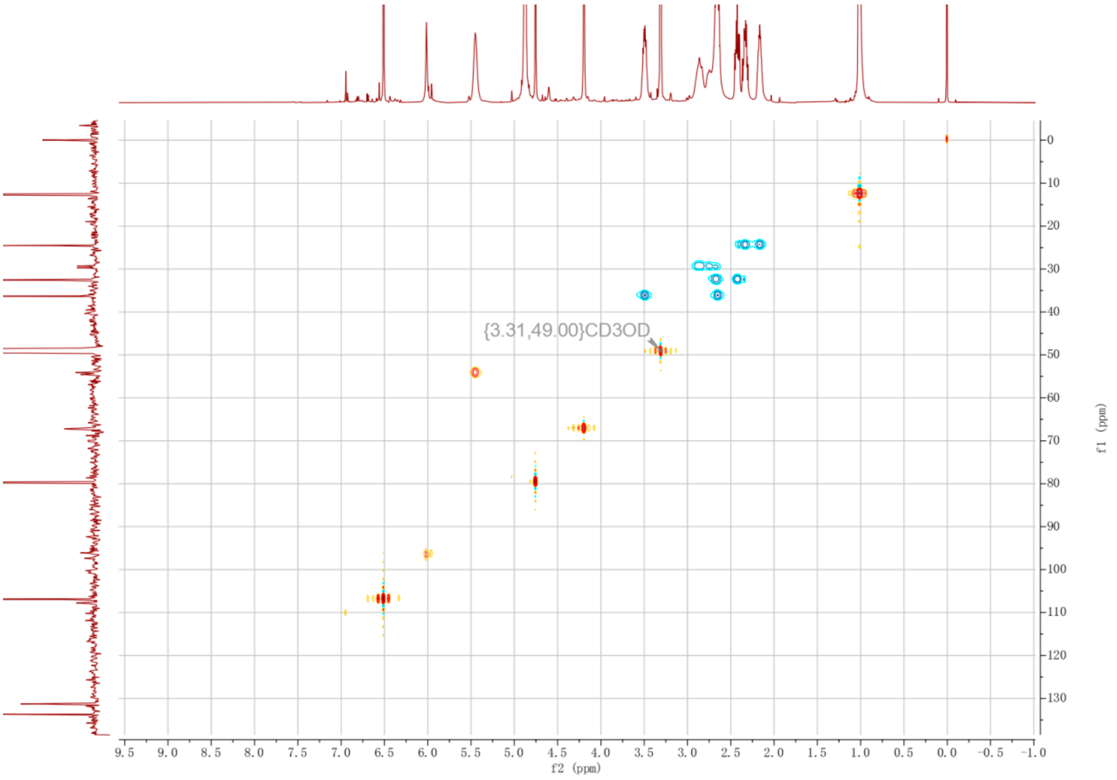


**Figure S6.** HSQC data of compound **11** in methanol-*d*4.

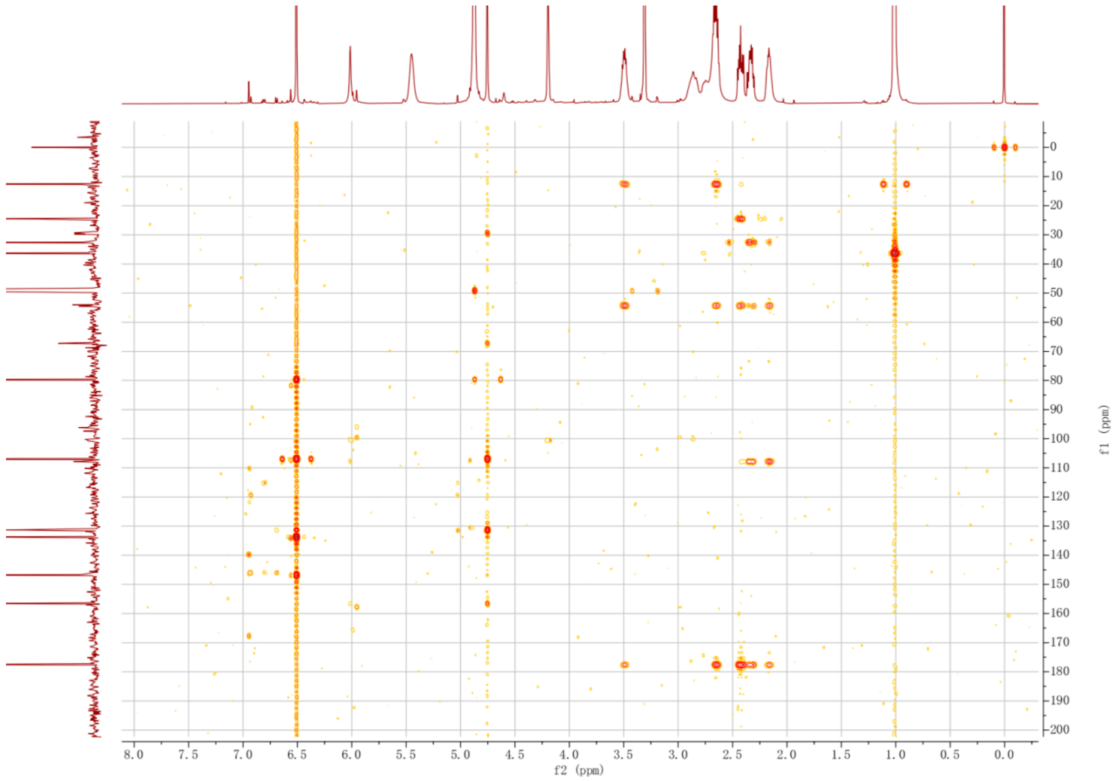


**Figure S7.** HMBC data of compound **11** in methanol-*d*4.

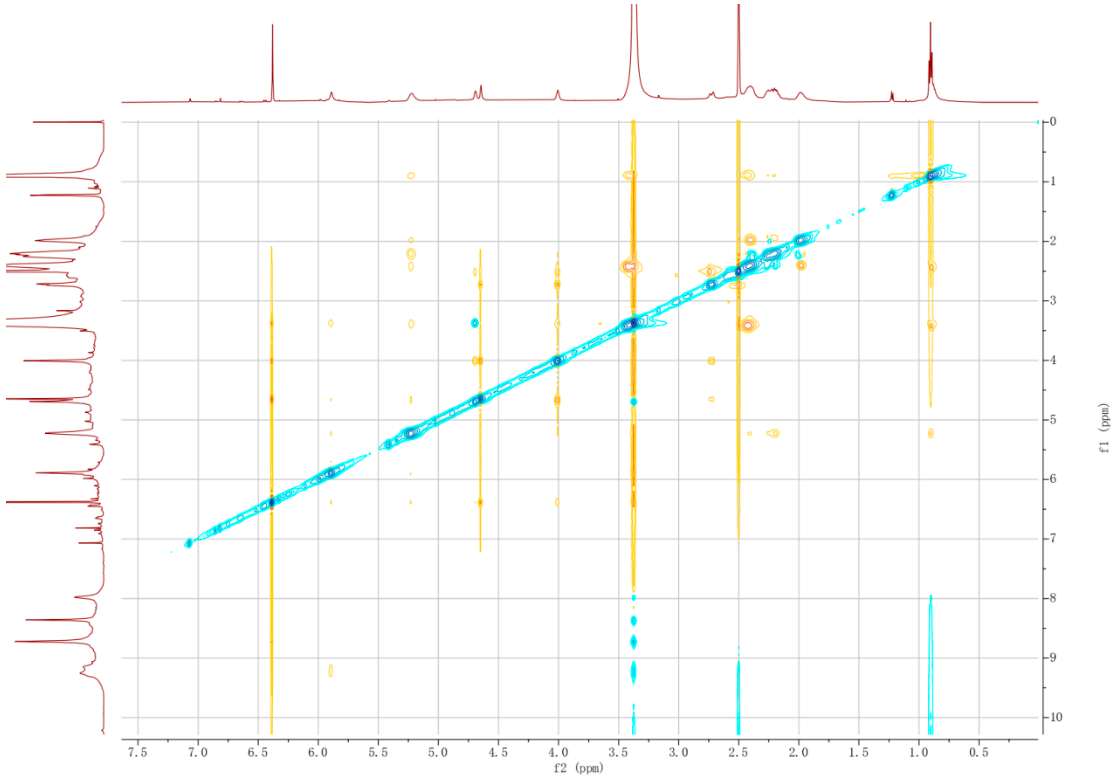


**Figure S8.** ROESY data of compound **11** in DMSO-*d*6.

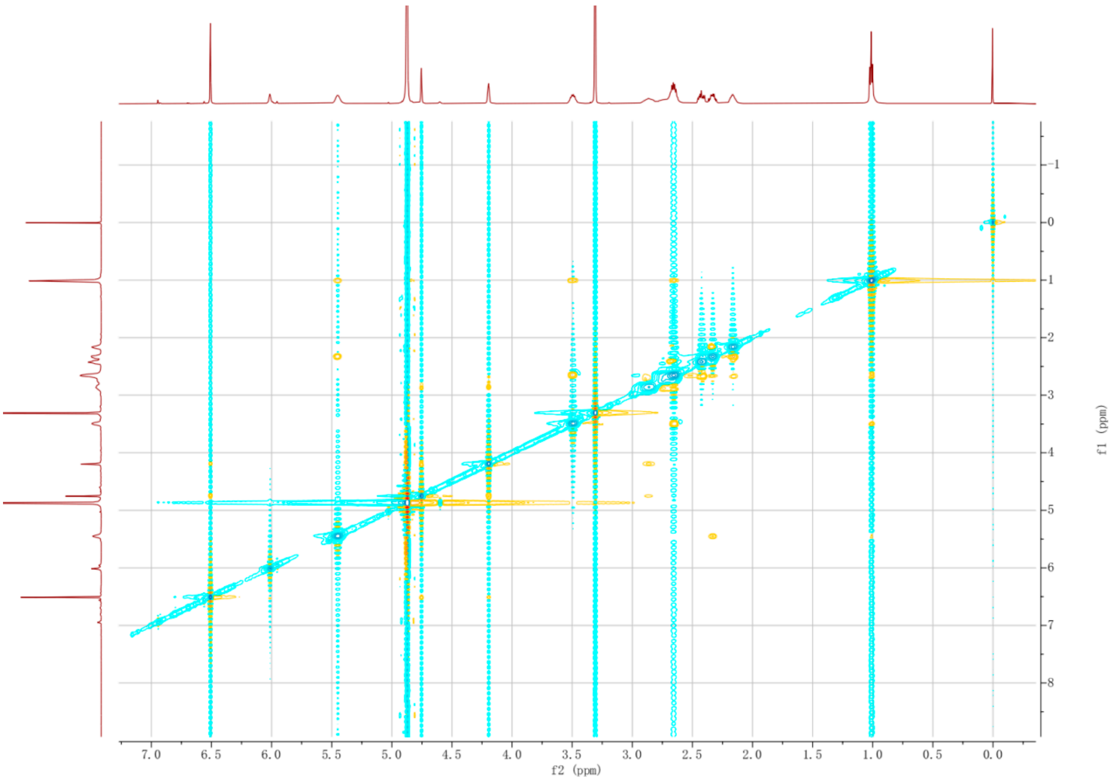


**Figure S9.** NOESY data of compound **11** in methanol-$d_4$.

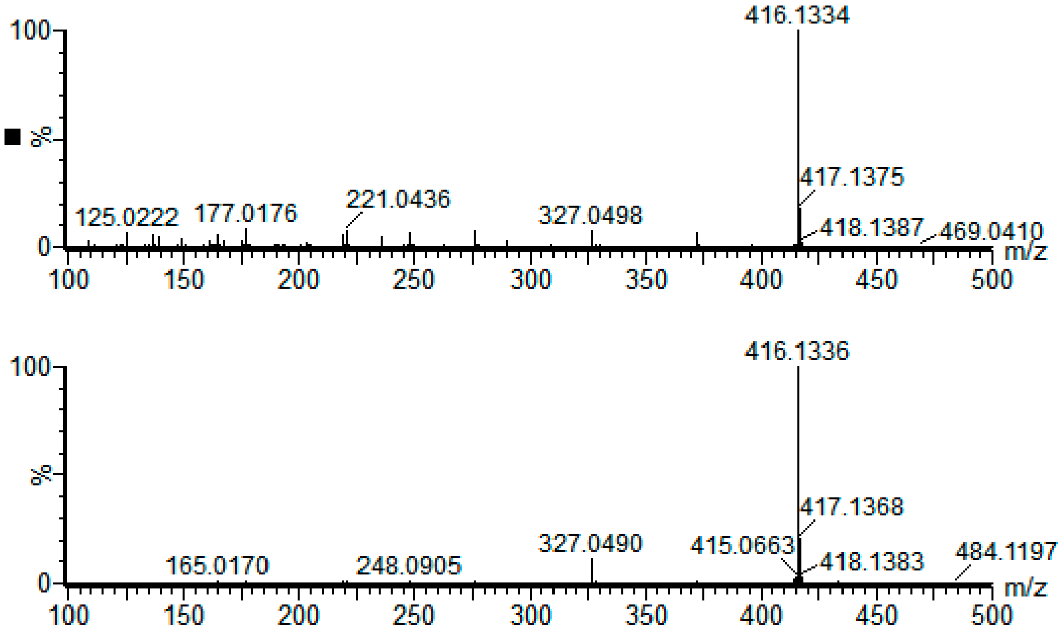


**Figure S10.** HR-ESI-MS data of compound **12** in the negative mode.

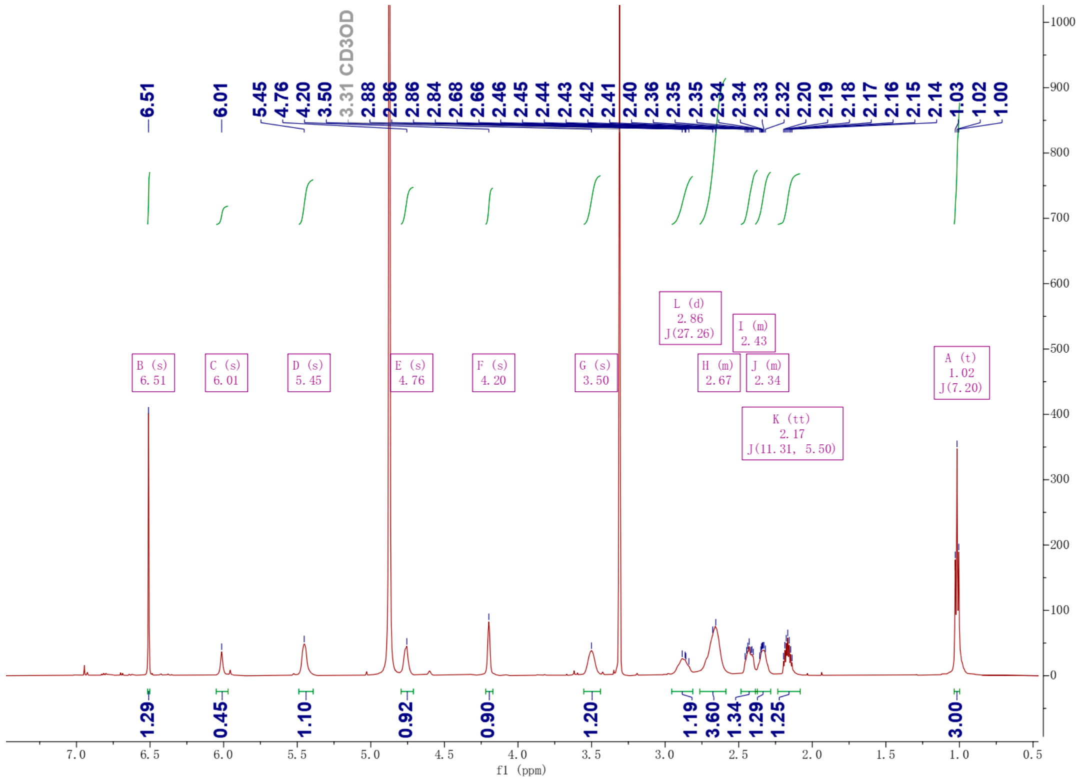

**Figure S11.** $^{1}$H NMR data of compound **12** in methanol-*d*4.

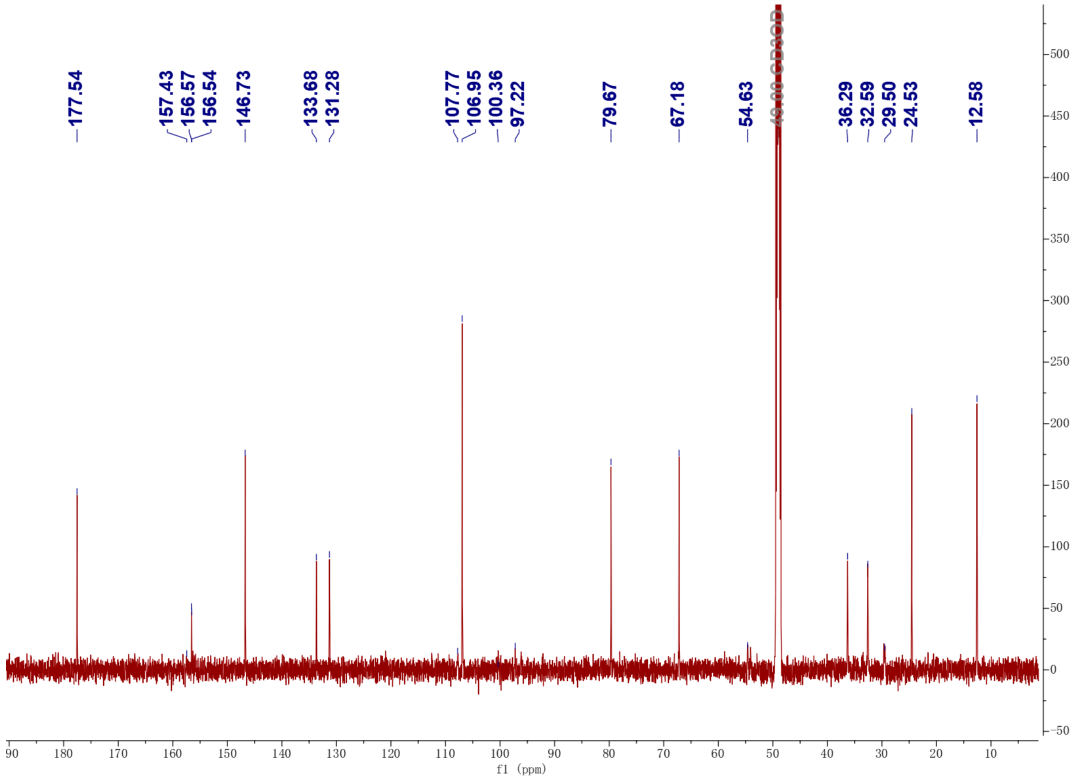

**Figure S12.** $^{13}$C NMR data of compound **12** in methanol-*d*4.

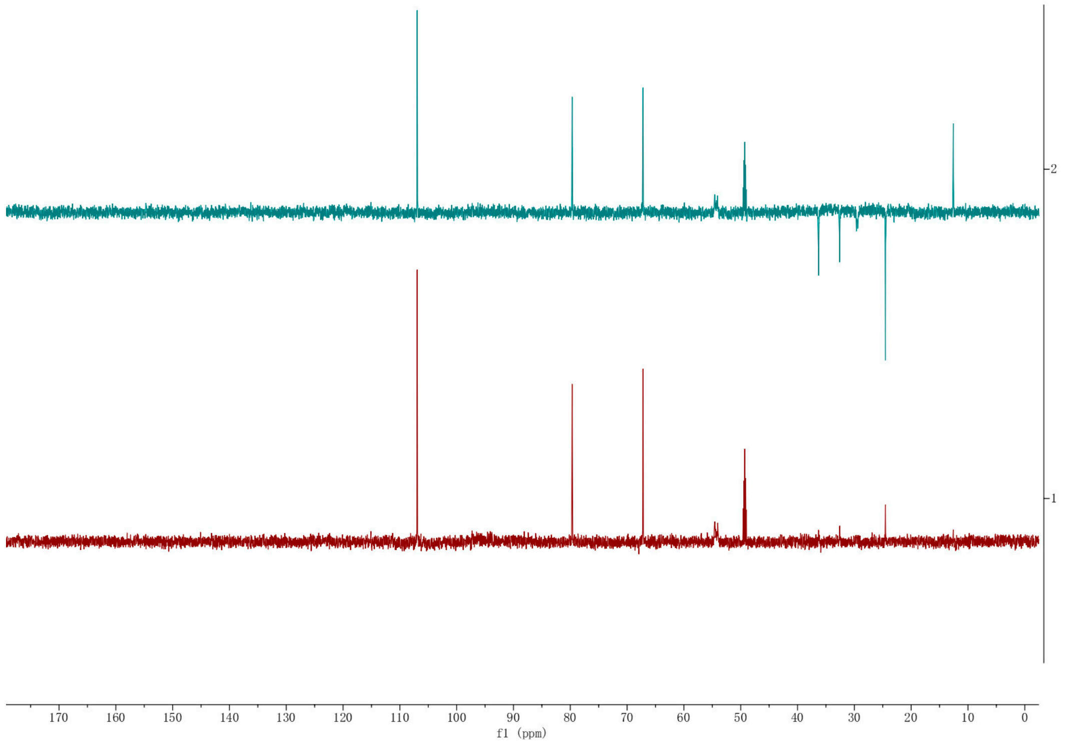


**Figure S13.** DEPT data of compound **12** in methanol-*d*4.

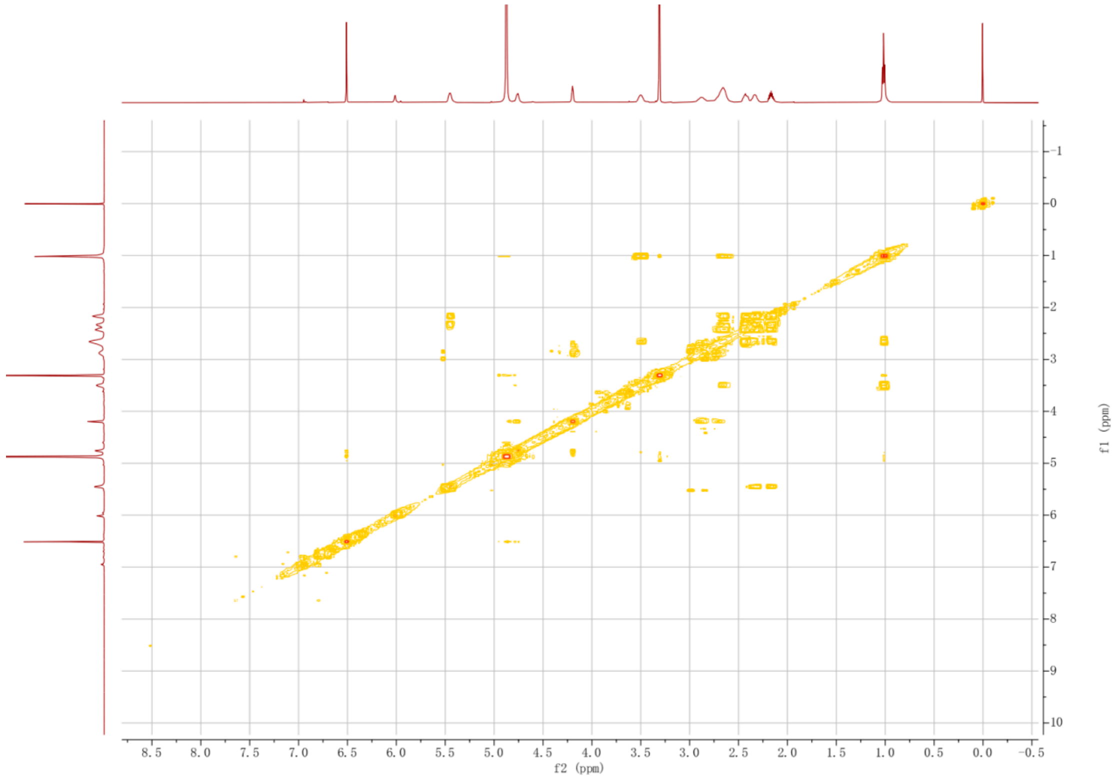


**Figure S14.** COSY data of compound **12** in methanol-*d*4.

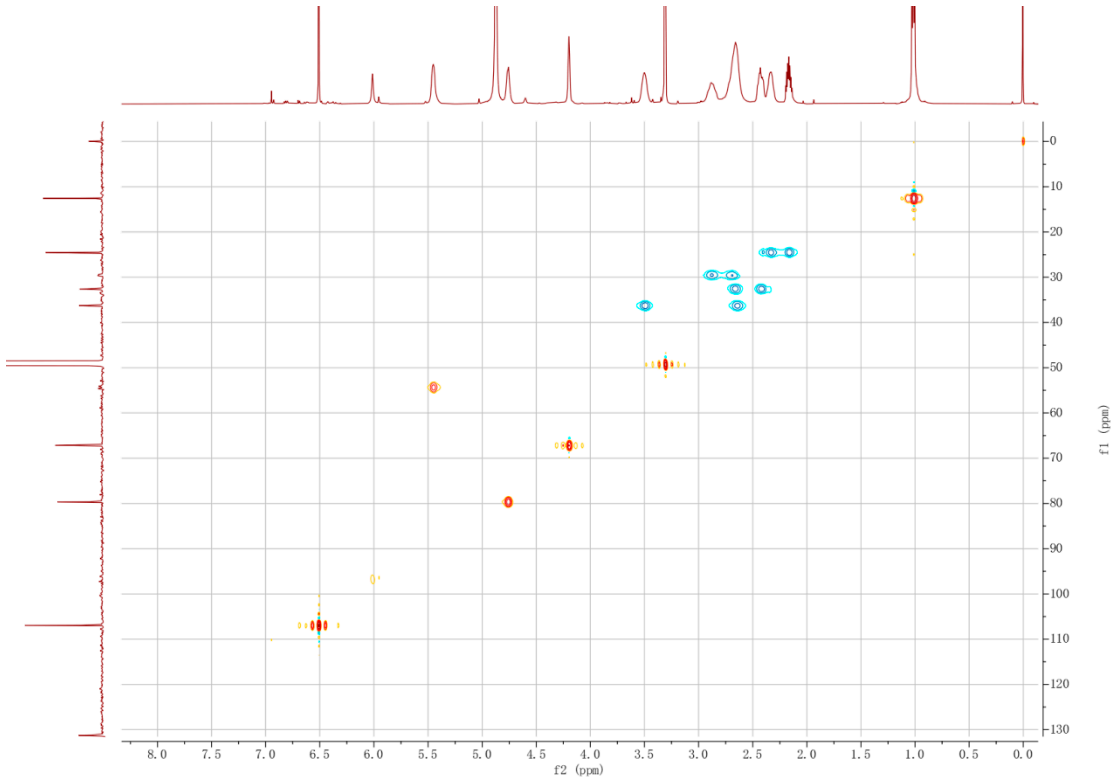


**Figure S15.** HSQC data of compound **12** in methanol-*d*4.

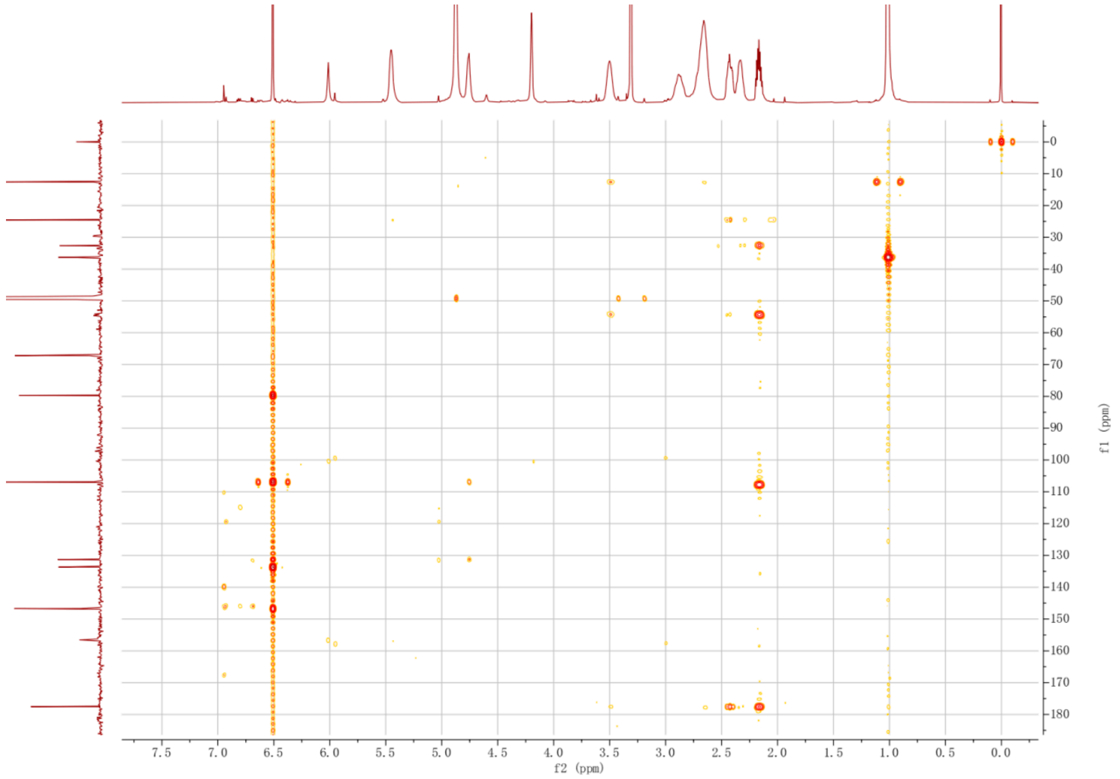


**Figure S16.** HMBC data of compound **12** in methanol-*d*4.

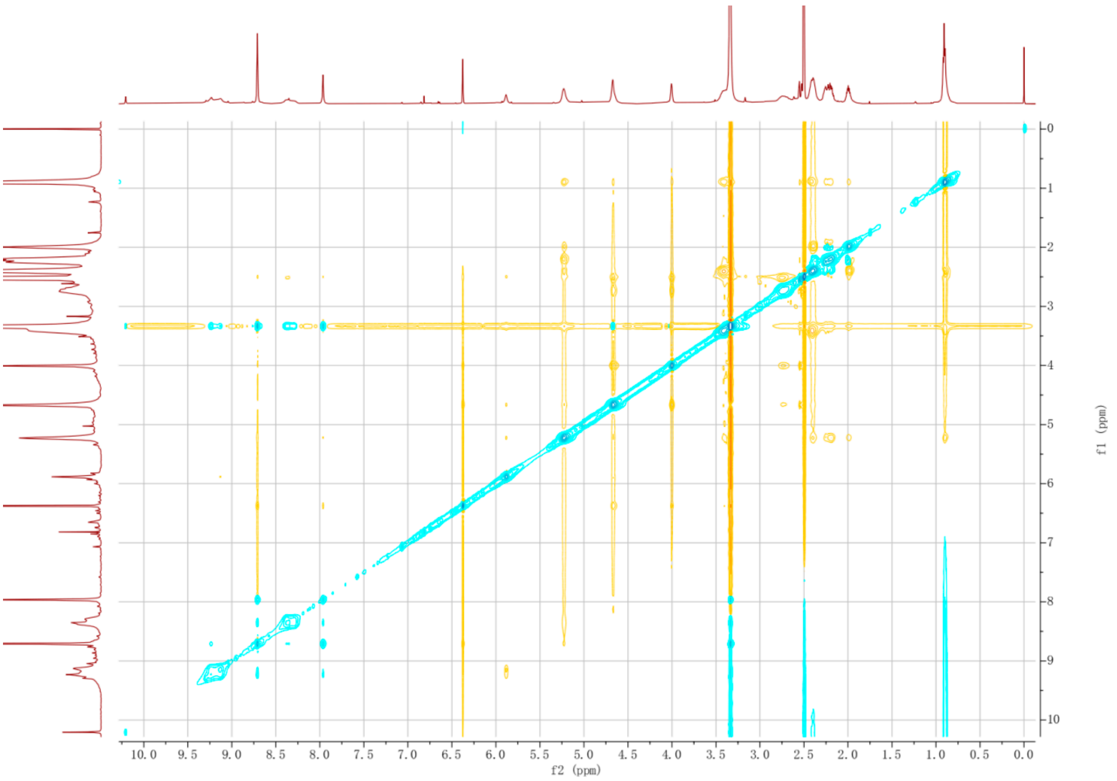


**Figure S17.** ROESY data of compound **12** in DMSO-$d_6$.

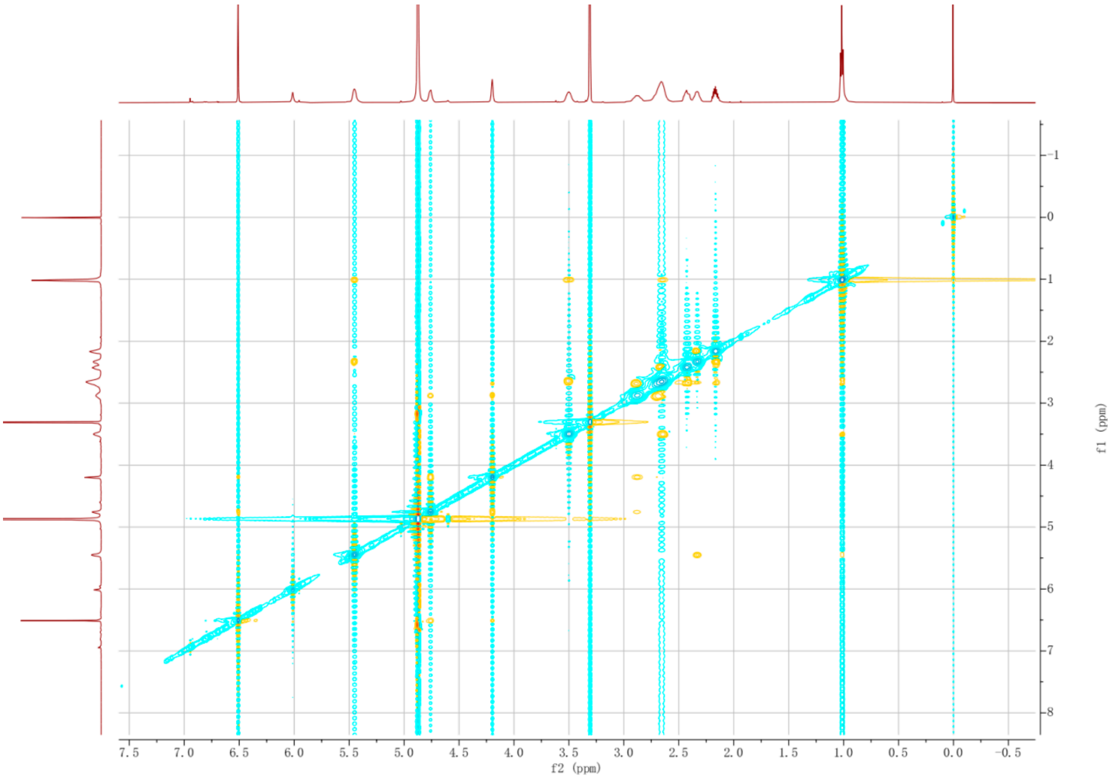


**Figure S18.** NOESY data of compound **12** in methanol-$d_4$.

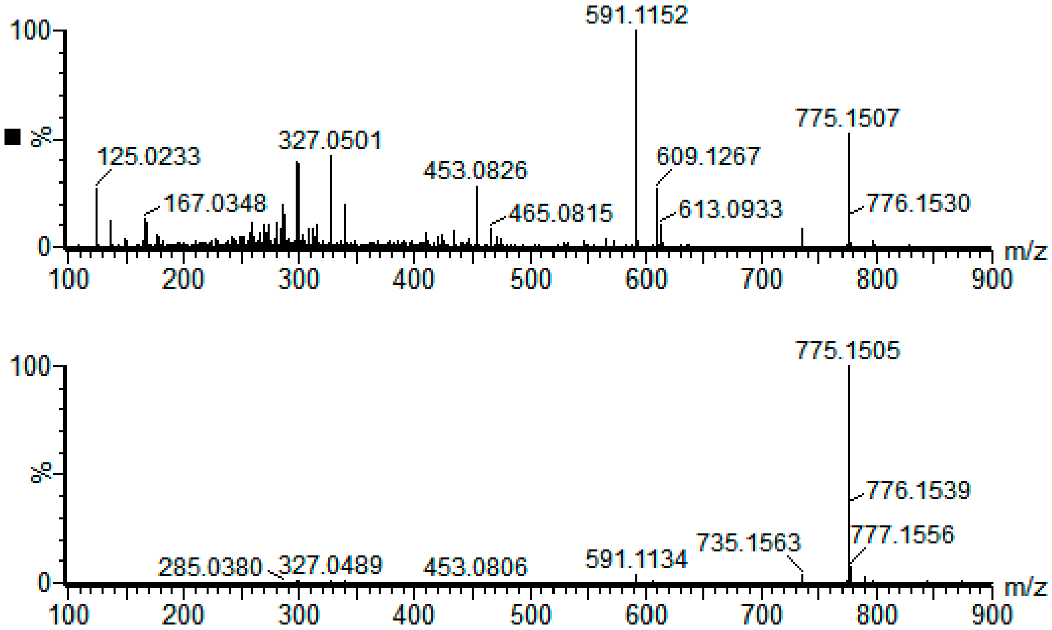


**Figure S19.** HR-ESI-MS data of compound **20** in the negative mode.

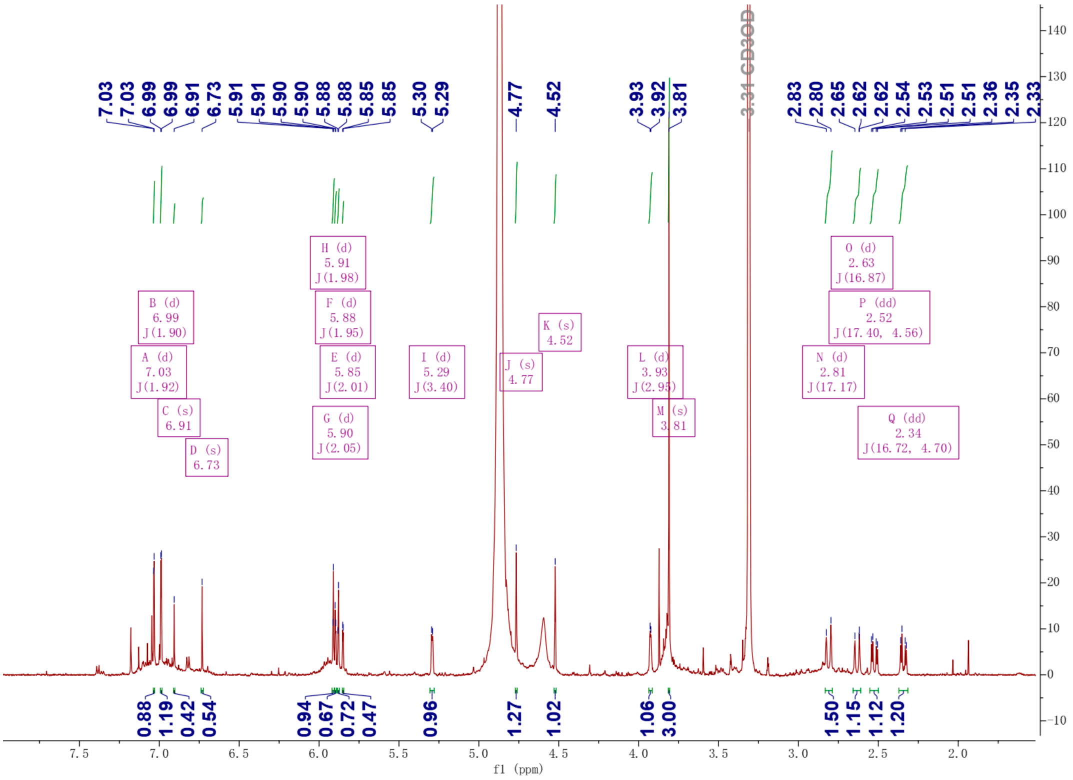


**Figure S20.** $^1$H NMR data of compound **20** in methanol-$d_4$.

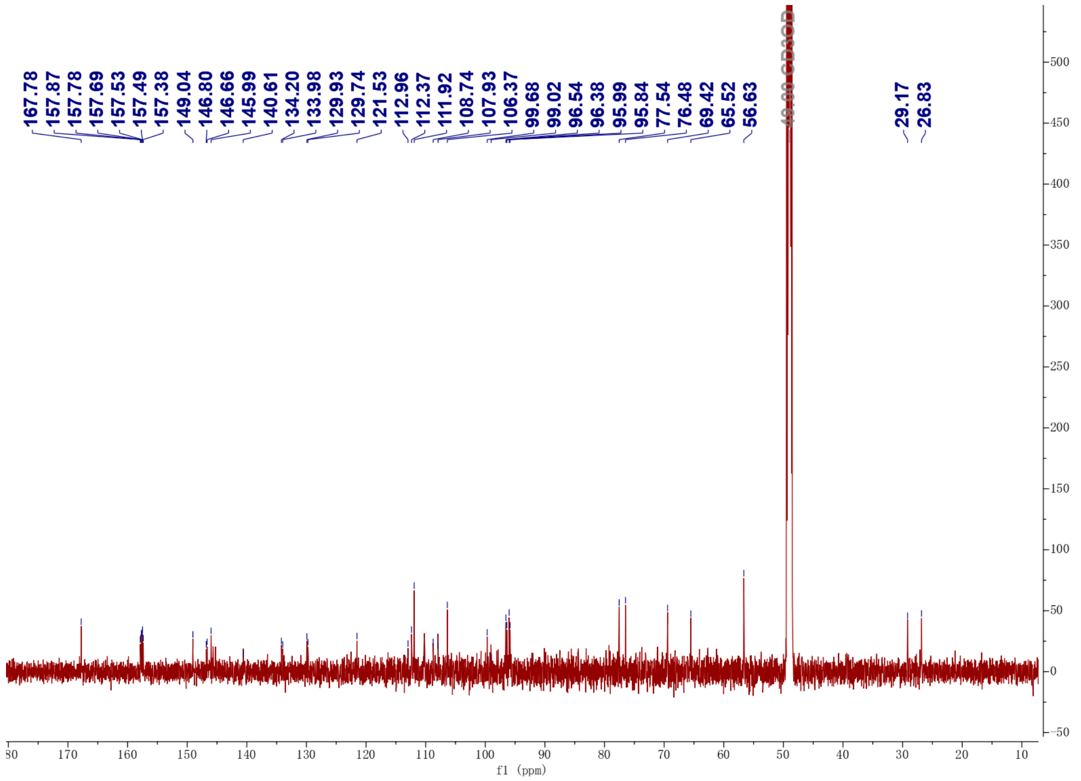


**Figure S21** $^{13}$C NMR data of compound **20** in methanol-$d_4$.

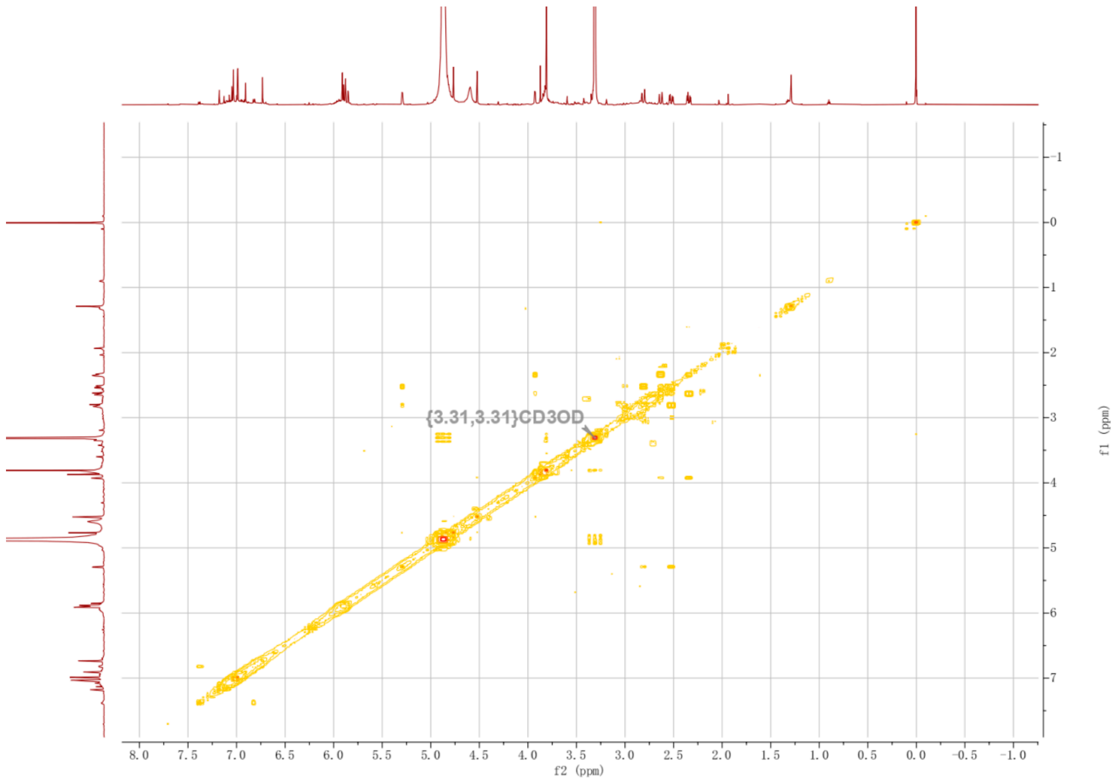


**Figure S22.** COSY data of compound **20** in methanol-$d_4$.

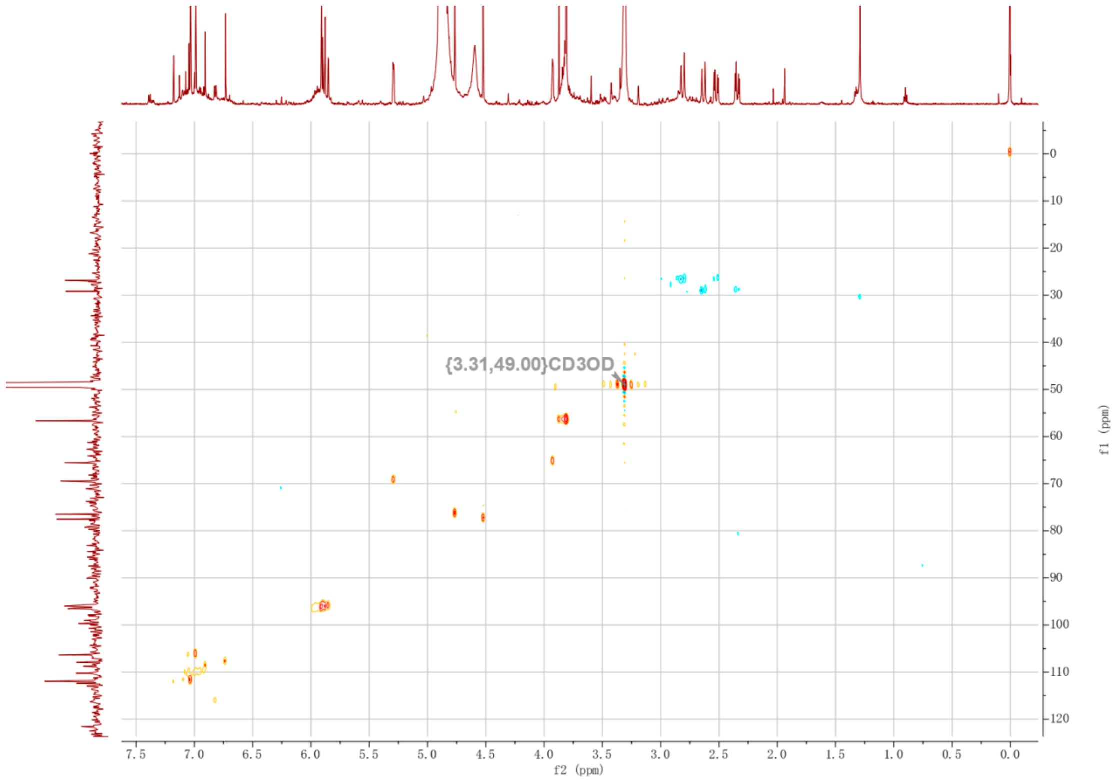


**Figure S23.** HSQC data of compound **20** in methanol-*d*4.

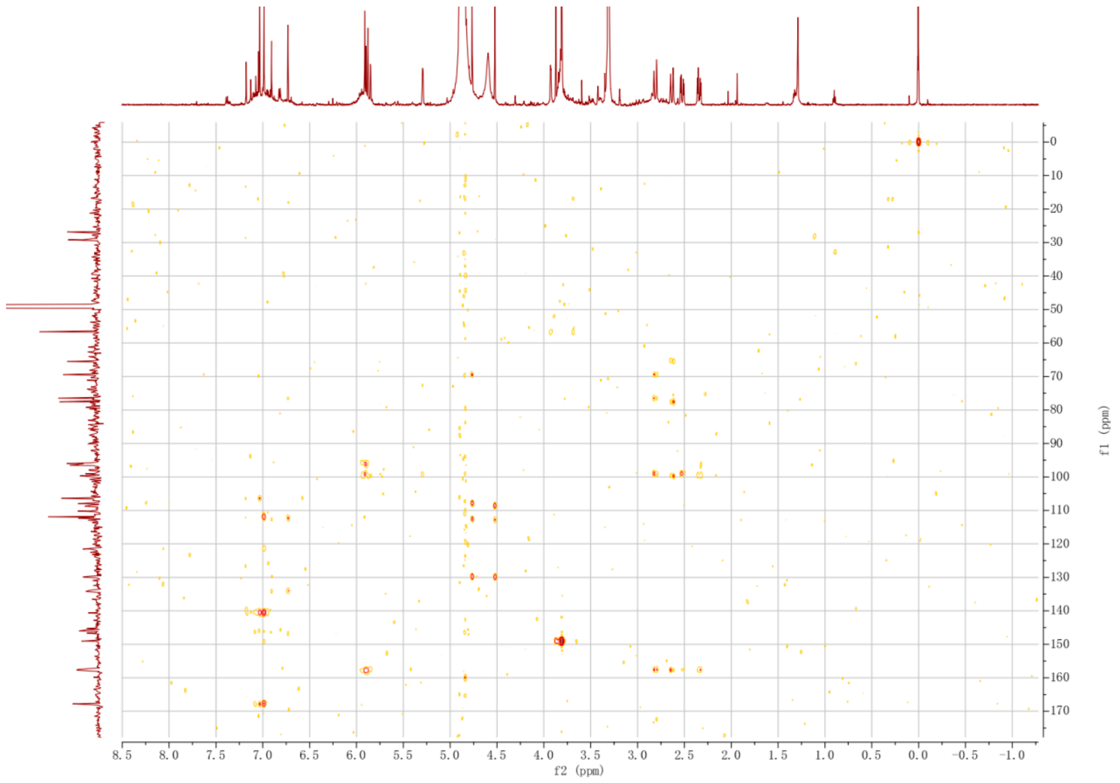


**Figure S24.** HMBC data of compound **20** in methanol-*d*4.

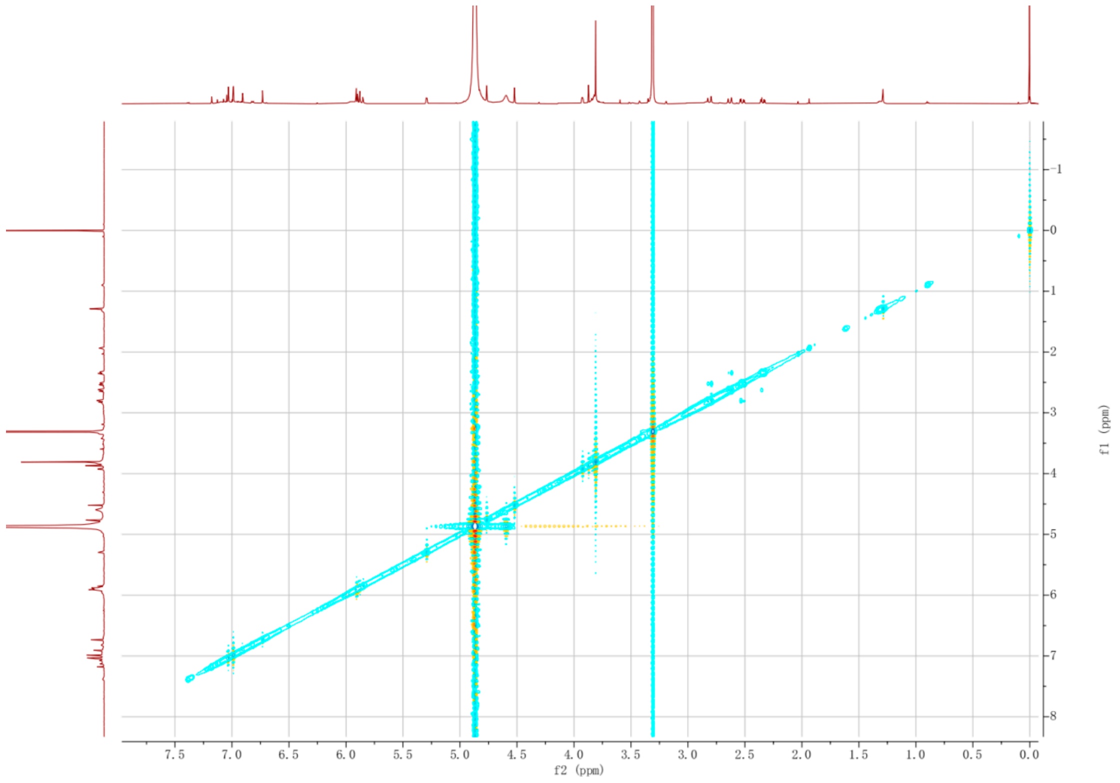


**Figure S25.** NOESY data of compound **20** in methanol-$d_4$.

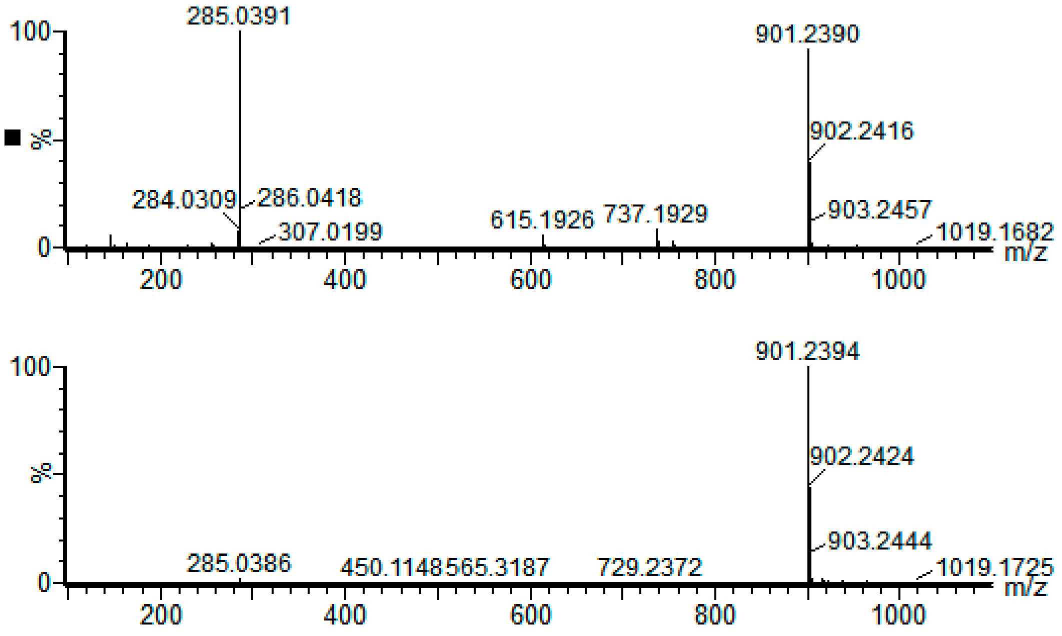


**Figure S26.** HR-ESI-MS data of compound **30** in the negative mode.

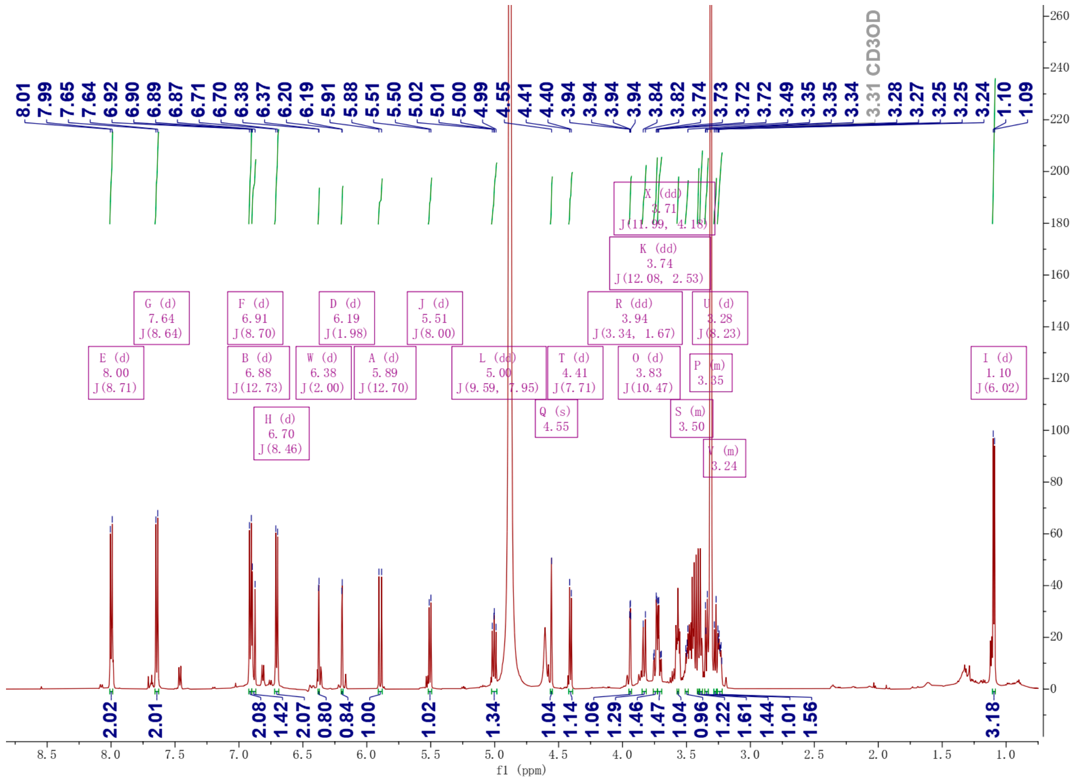


**Figure S27.** $^{1}$H NMR data of compound **30** in methanol-$d_4$.

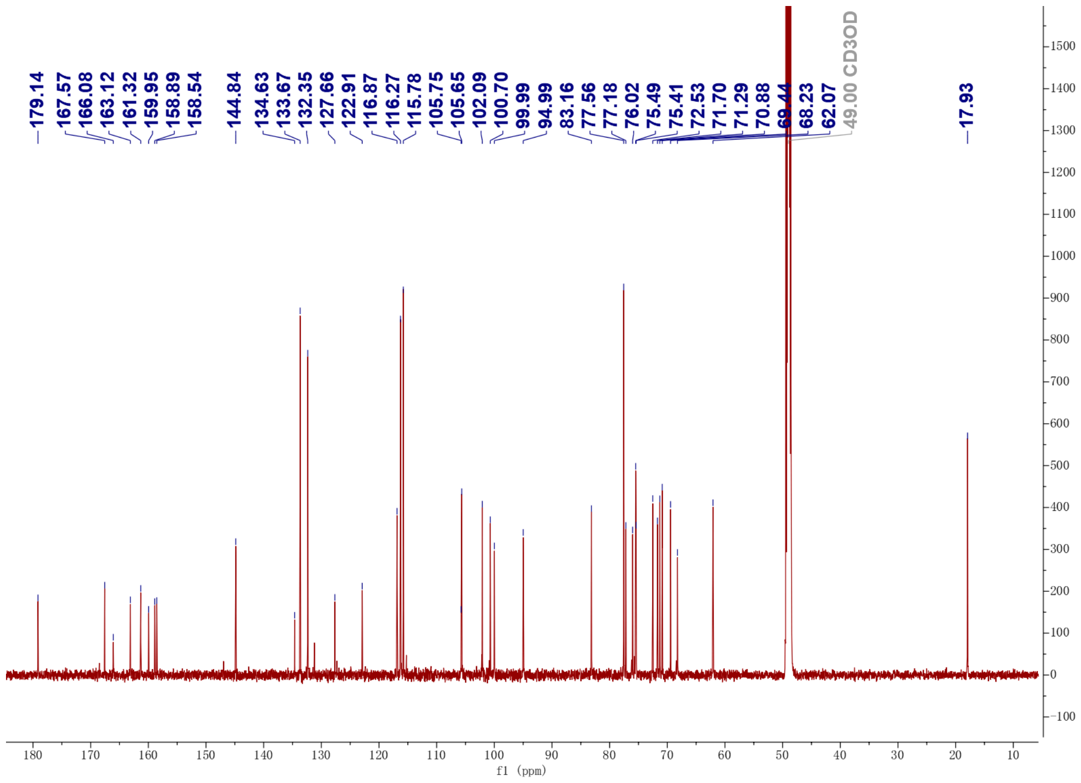


**Figure S28.** $^{13}$C NMR data of compound **30** in methanol-$d_4$.

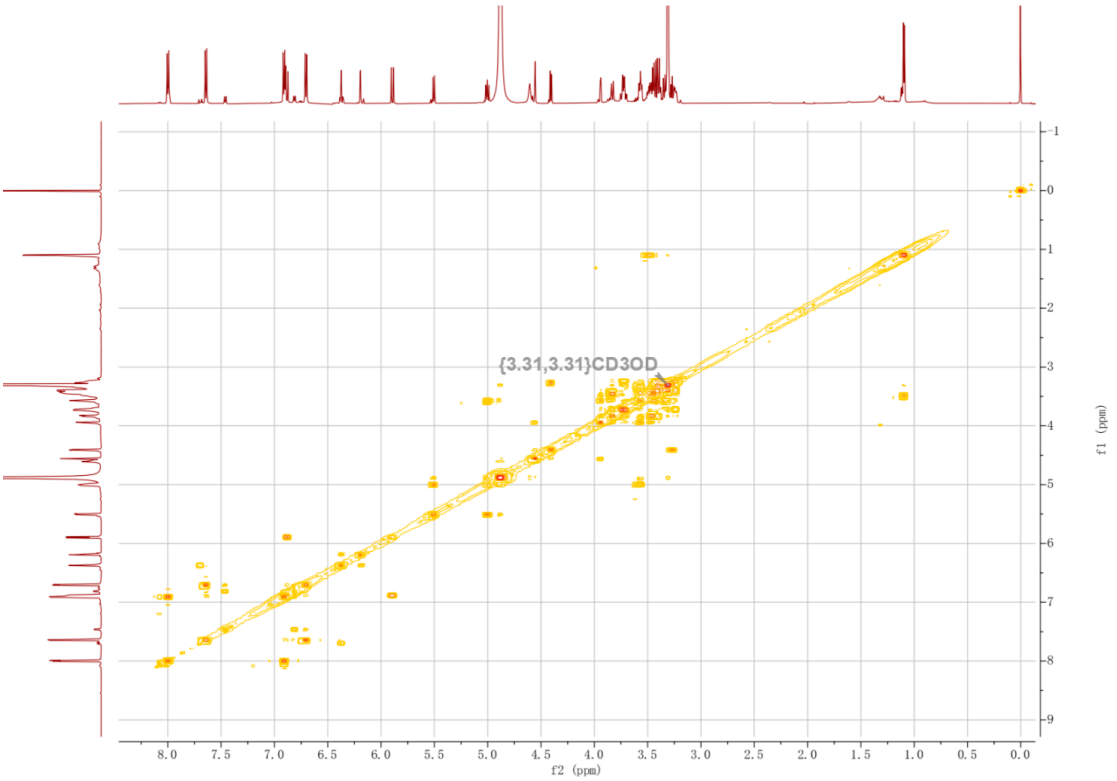


**Figure S29.** COSY data of compound **30** in methanol-*d*4.

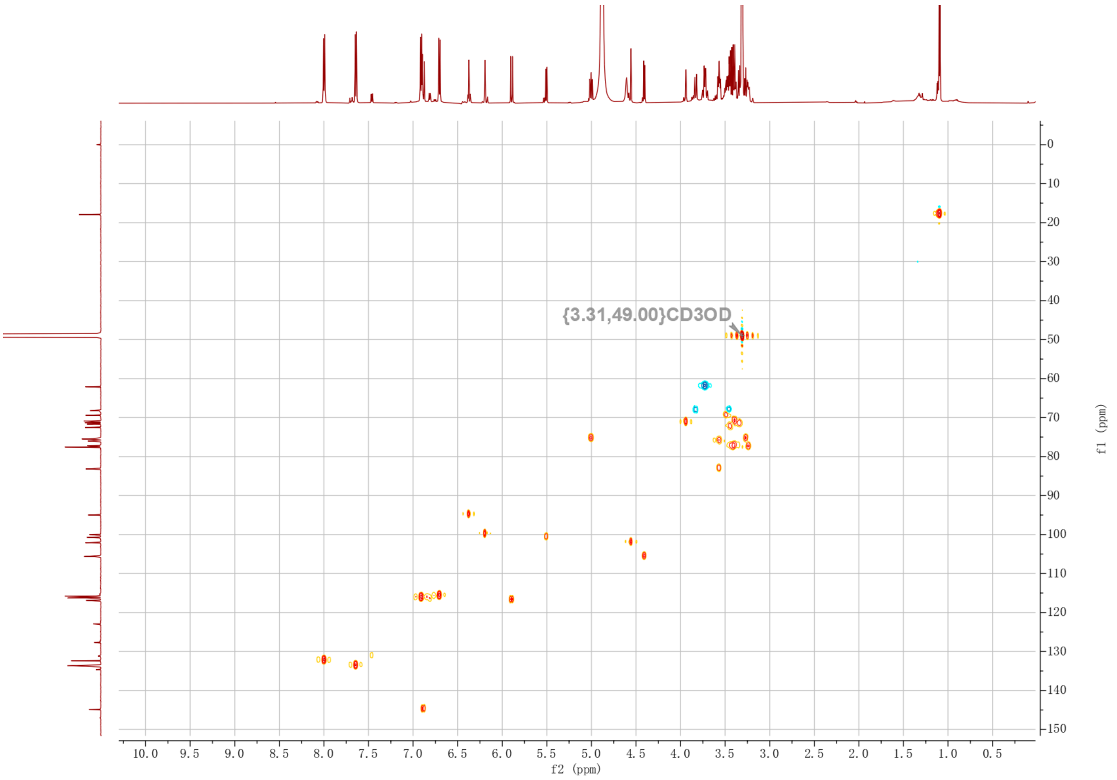


**Figure S30.** HSQC data of compound **30** in methanol-*d*4.

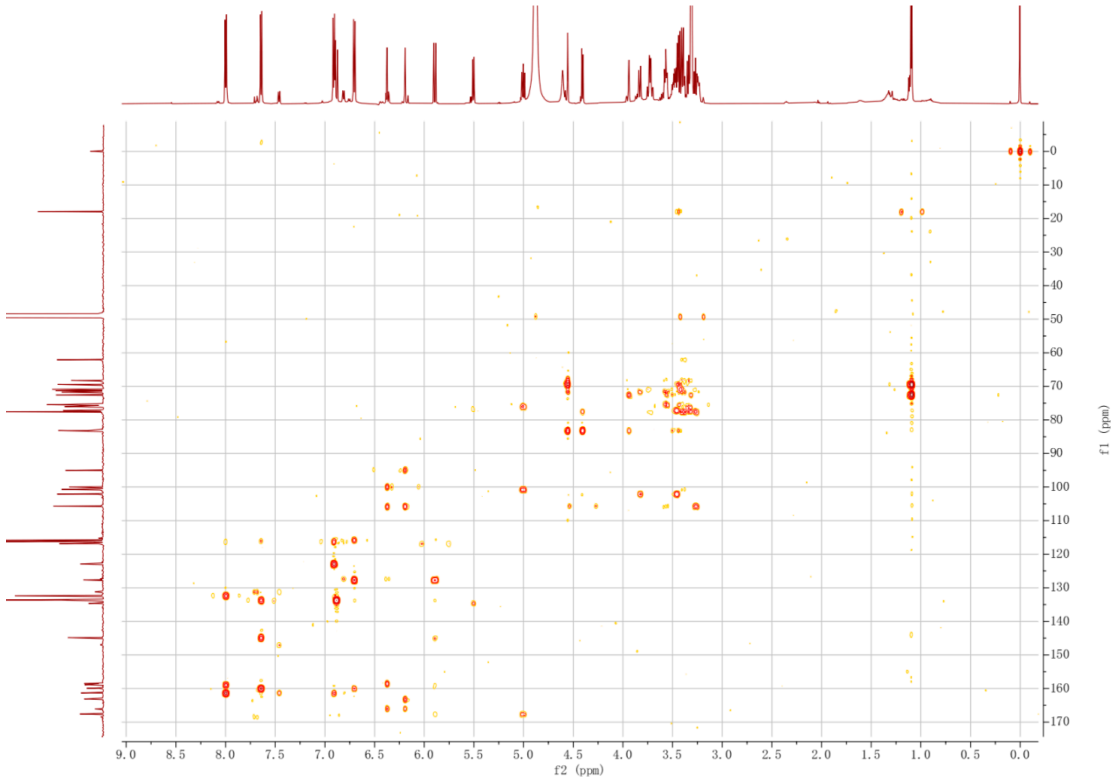


**Figure S31.** HMBC data of compound **30** in methanol-*d*4.

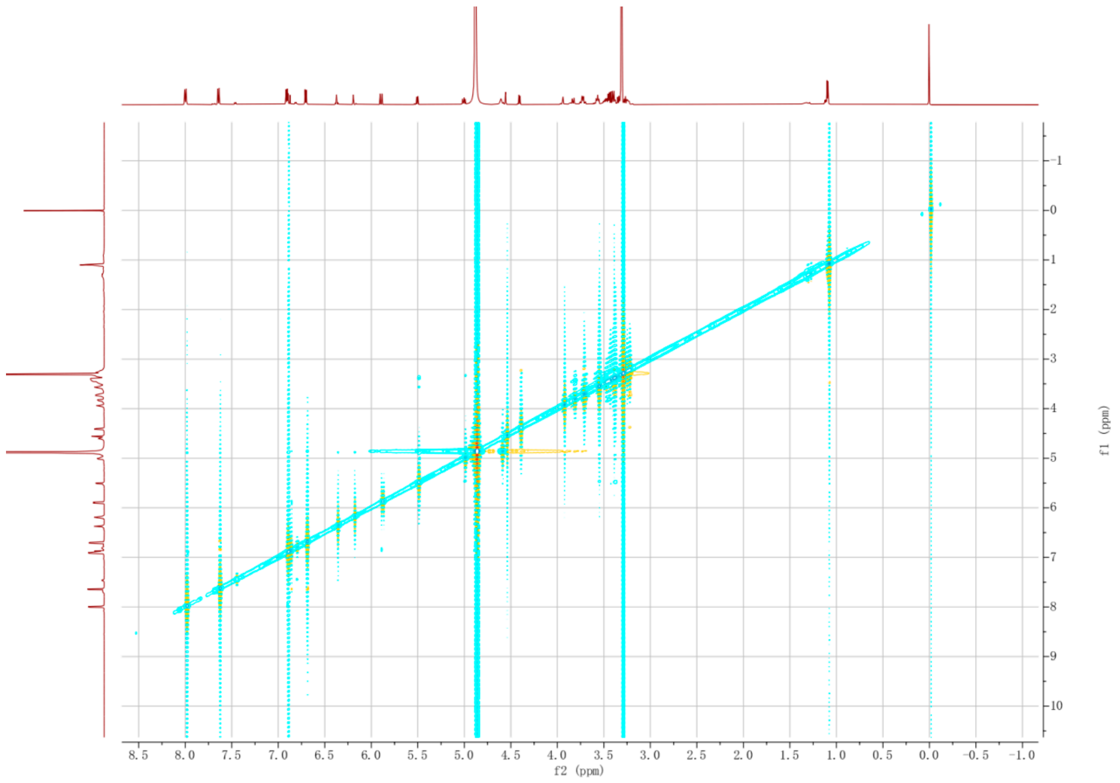


**Figure S32.** NOESY data of compound **30** in methanol-*d*4.

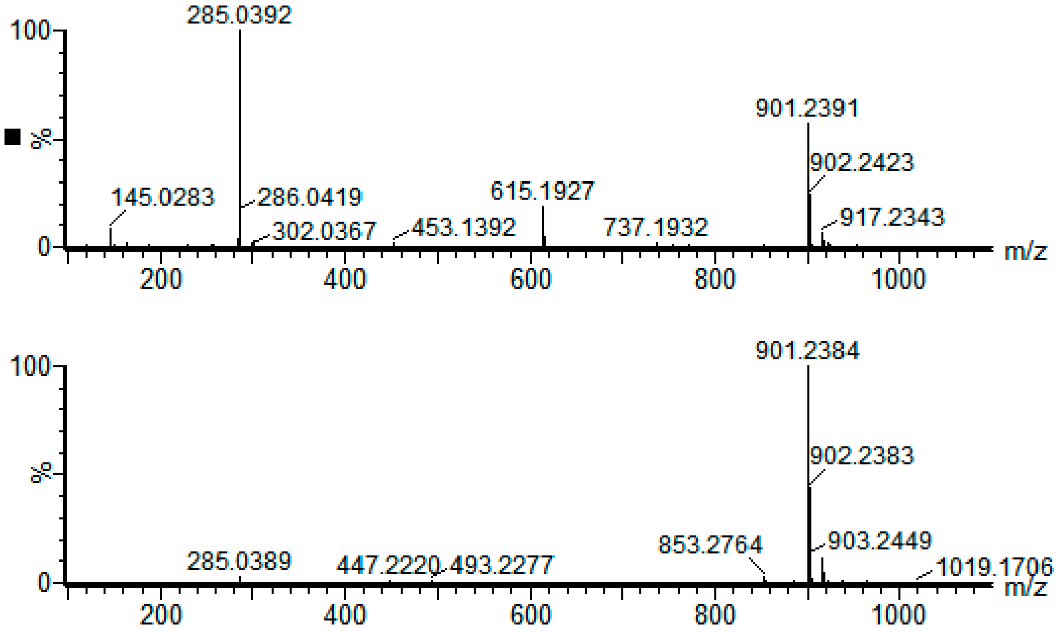


**Figure S33.** HR-ESI-MS data of compound **32** in the negative mode.

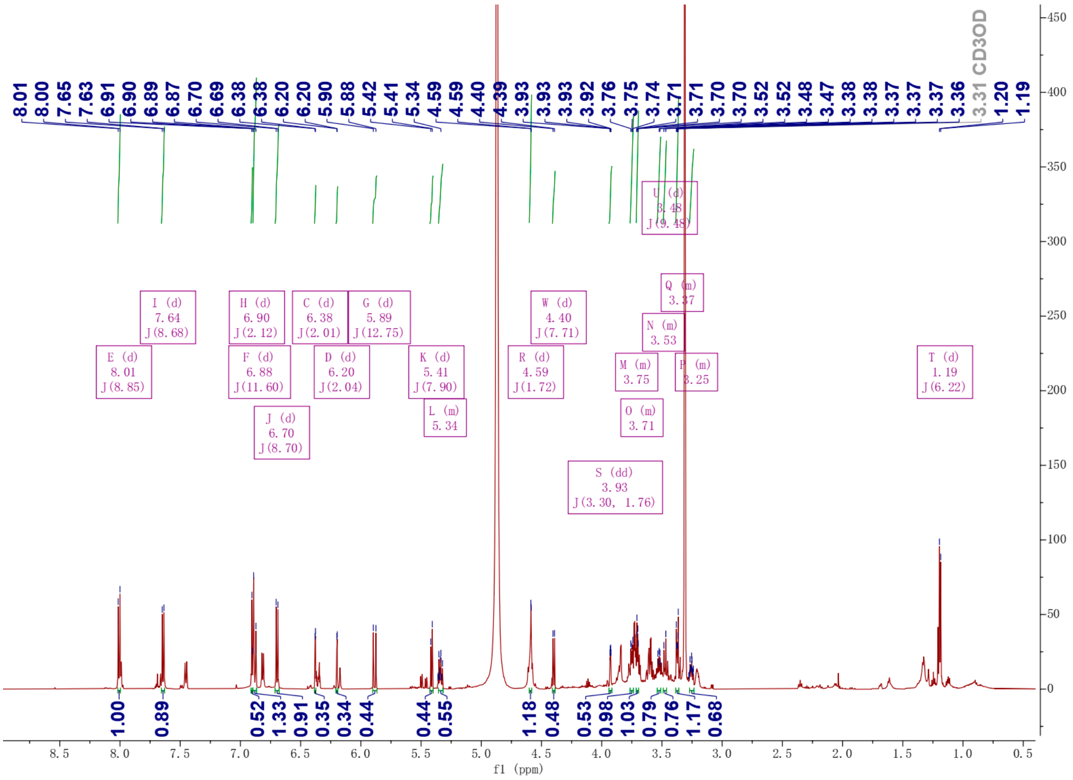


**Figure S34.** $^{1}$H NMR data of compound **32** in methanol-$d_4$.

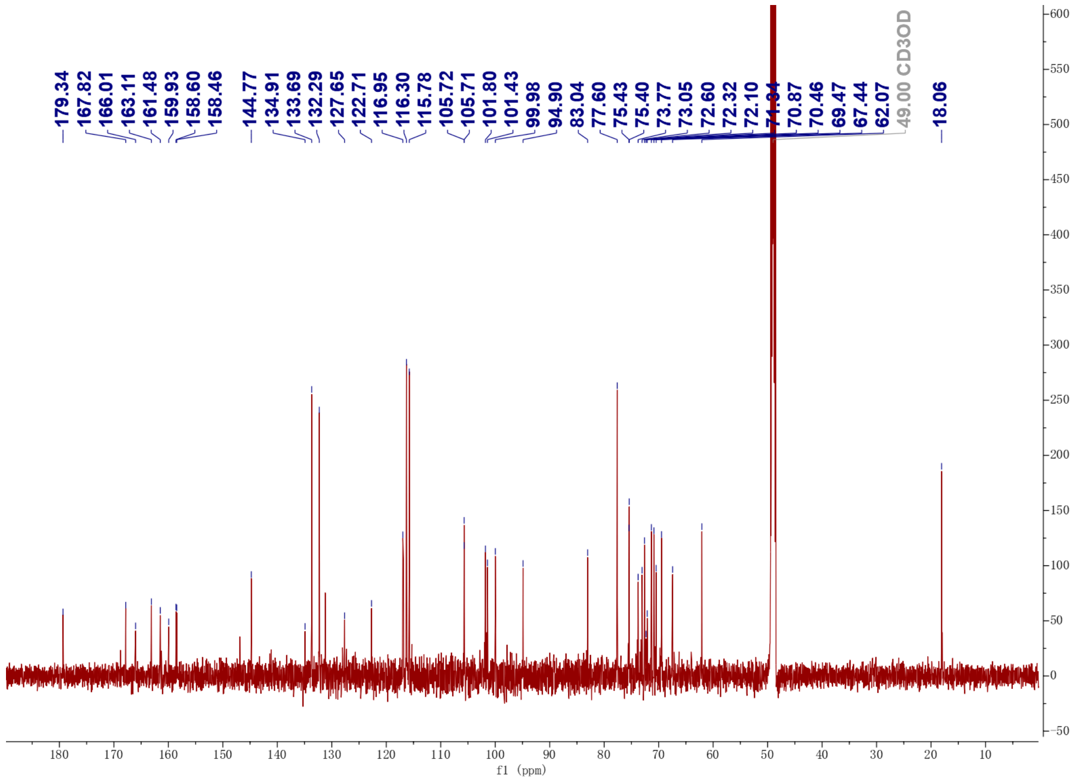


**Figure S35.** $^{13}$C NMR data of compound **32** in methanol-$d_4$.

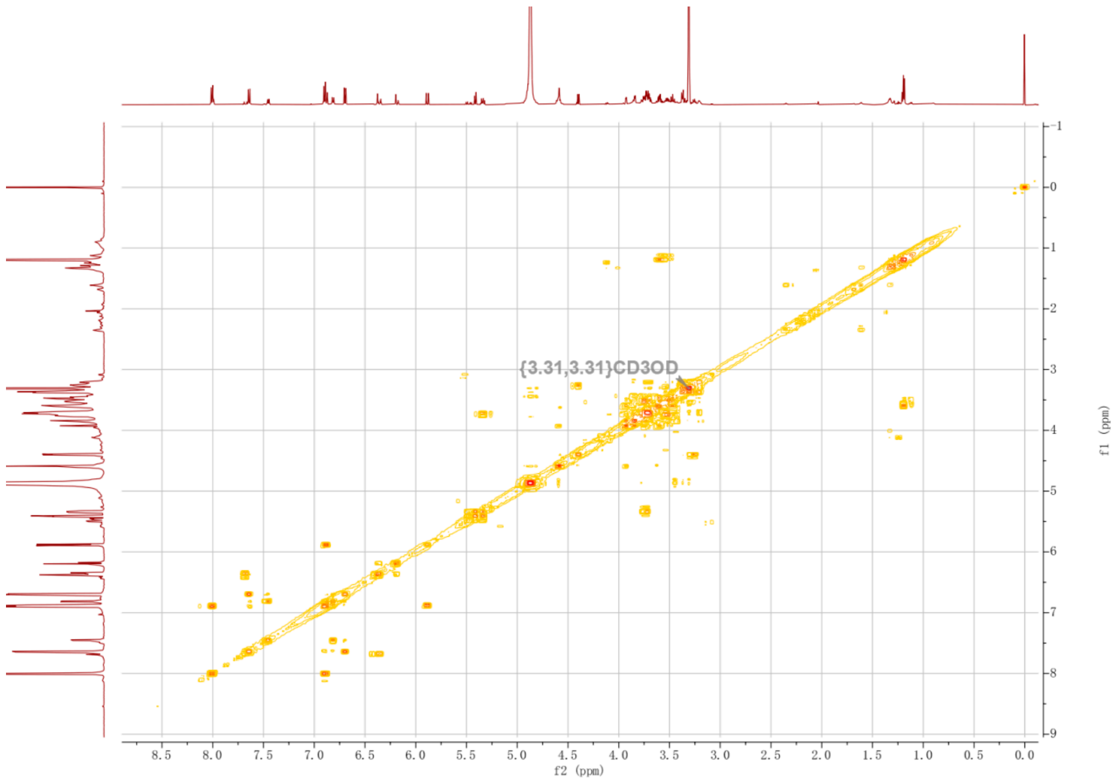


**Figure S36.** COSY data of compound **32** in methanol-$d_4$.

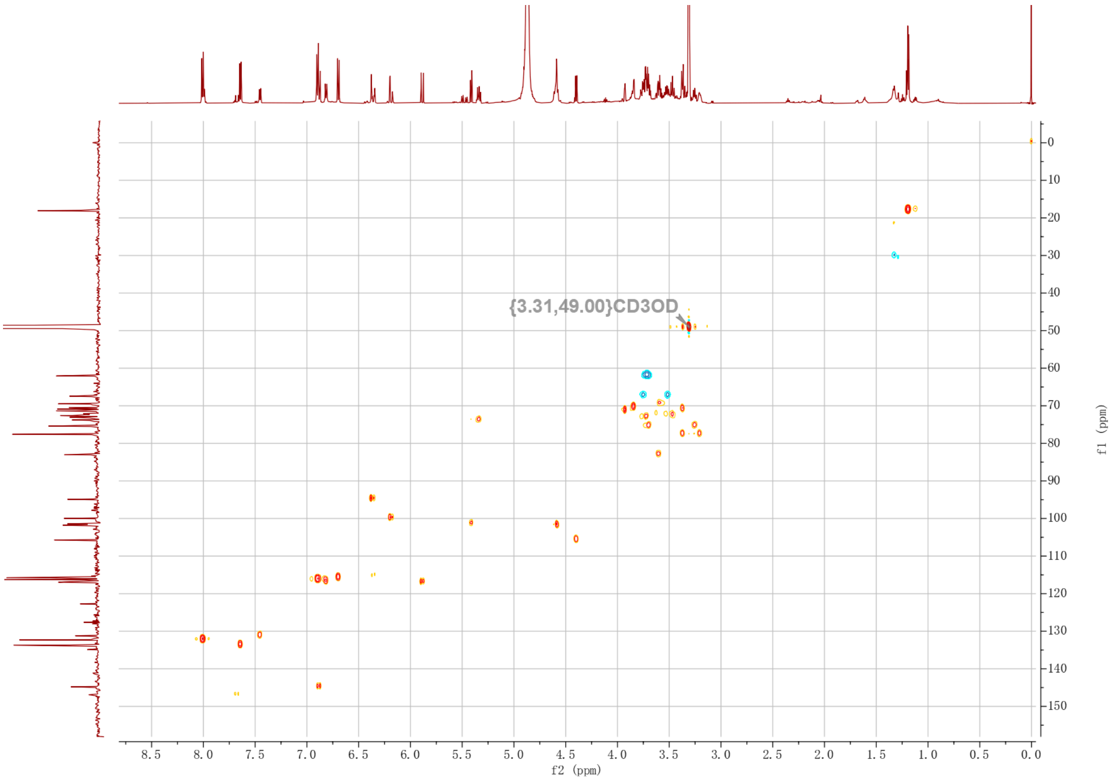


**Figure S37.** HSQC data of compound **32** in methanol-$d_4$.

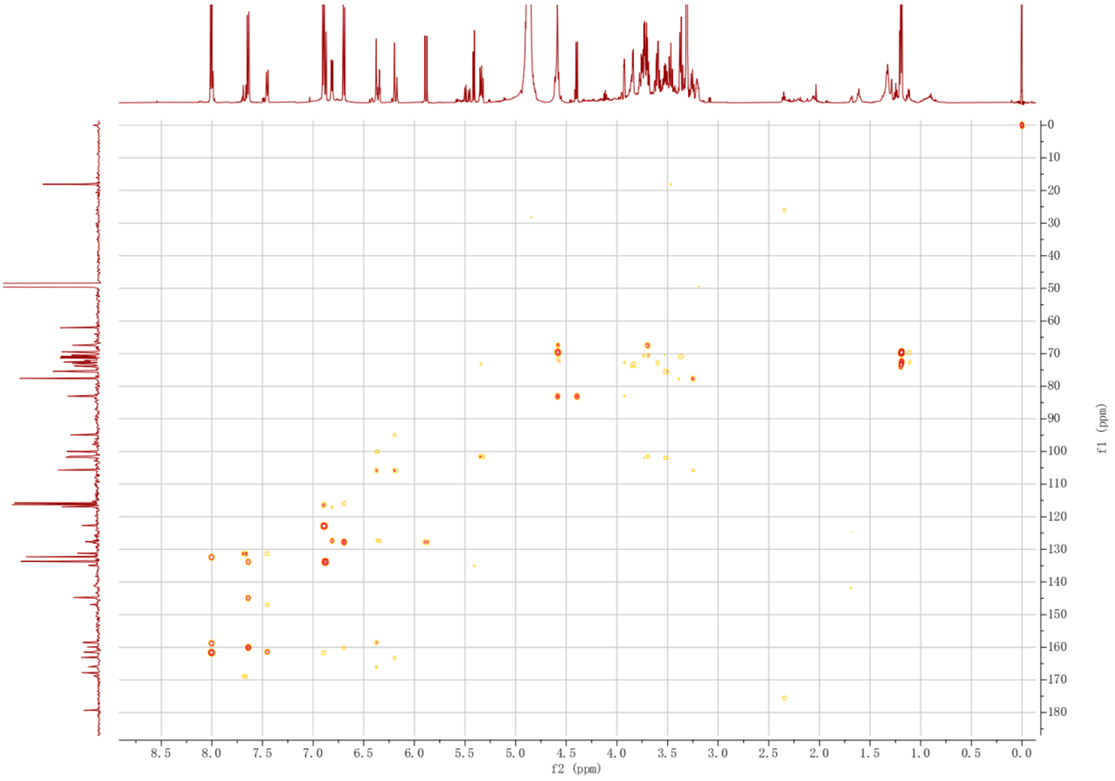


**Figure S38.** HMBC data of compound **32** in methanol-$d_4$.

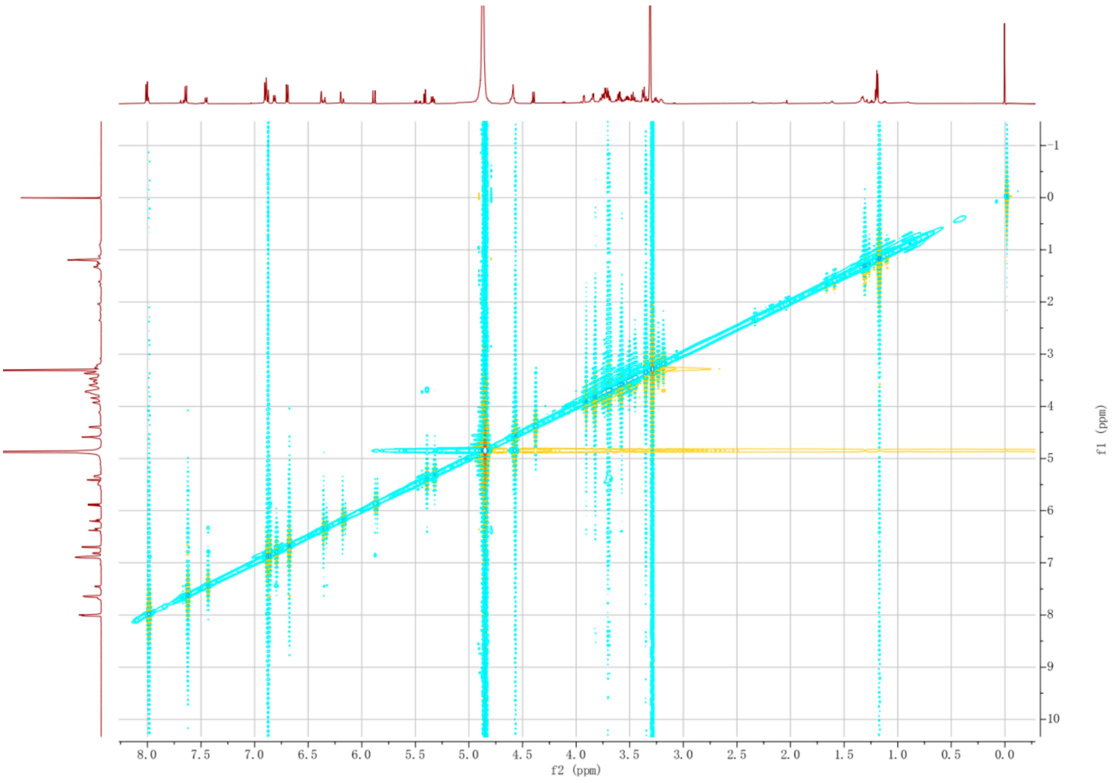


**Figure S39.** NOESY data of compound **32** in methanol-*d*4.

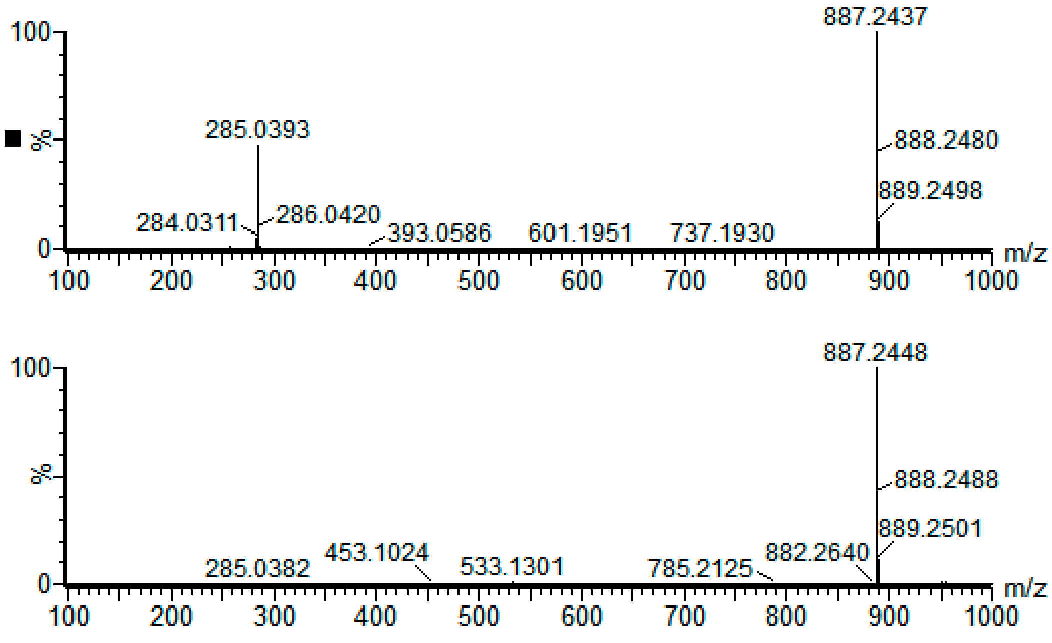


**Figure S40.** HR-ESI-MS data of compound **34** in the negative mode.

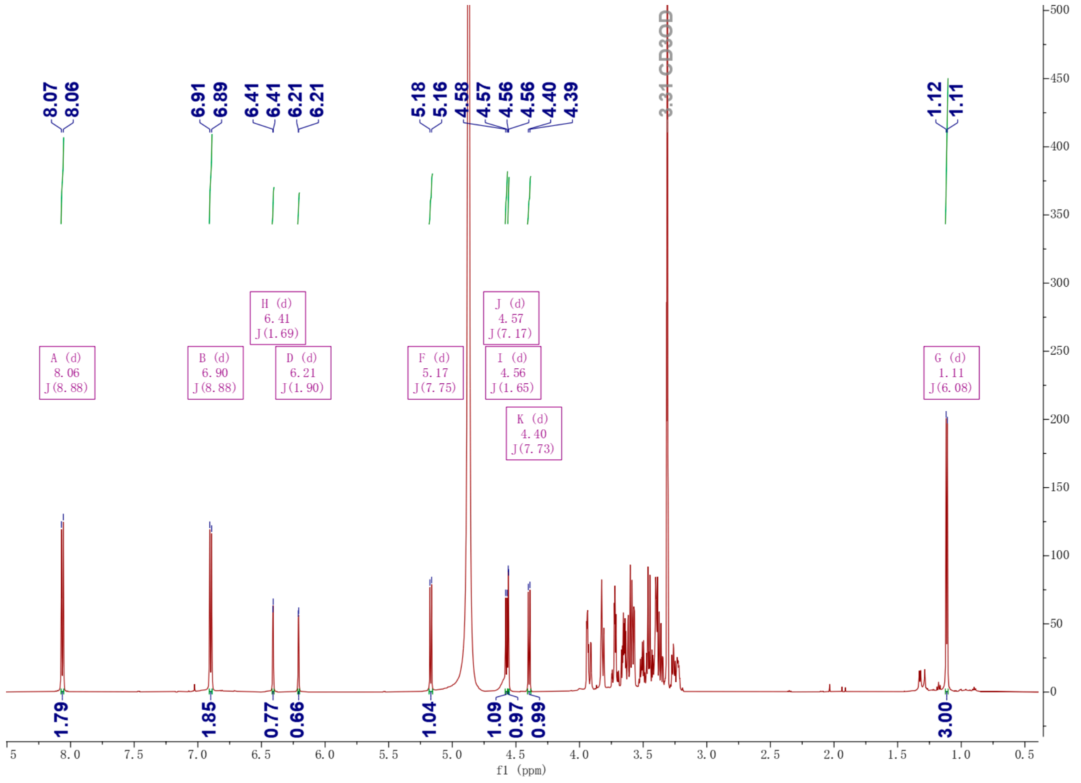

**Figure S41.** $^{1}$H NMR data of compound **34** in methanol-$d_4$.

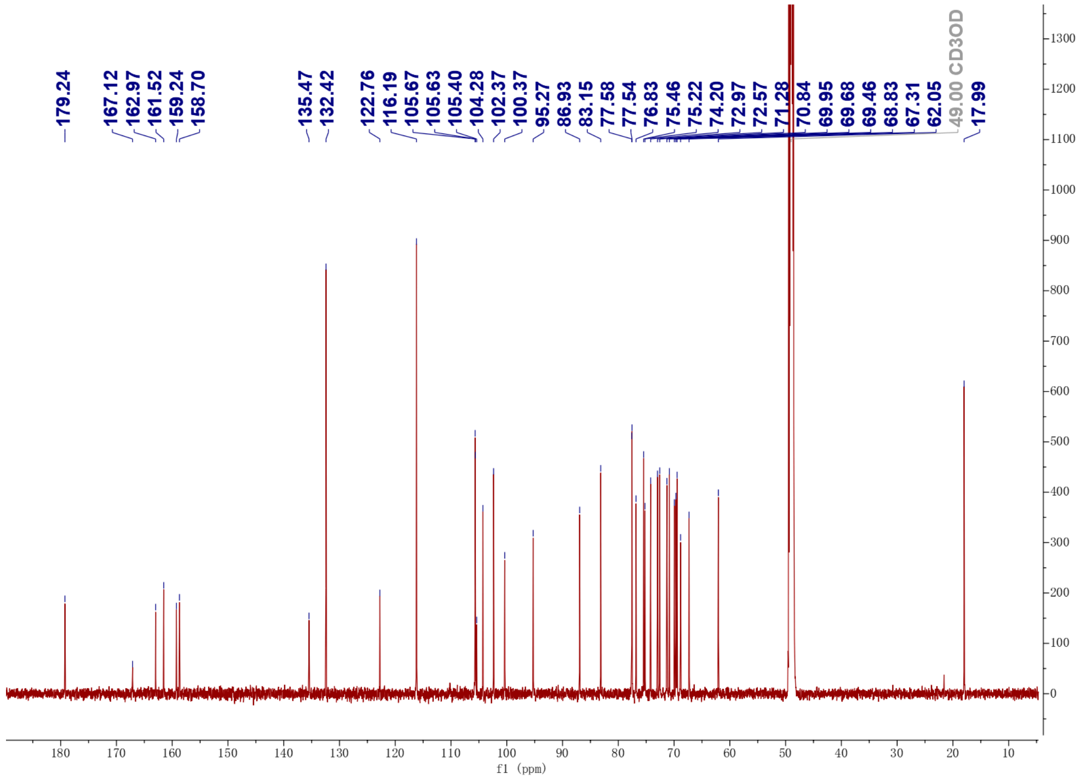

**Figure S42.** $^{13}$C NMR data of compound **34** in methanol-$d_4$.

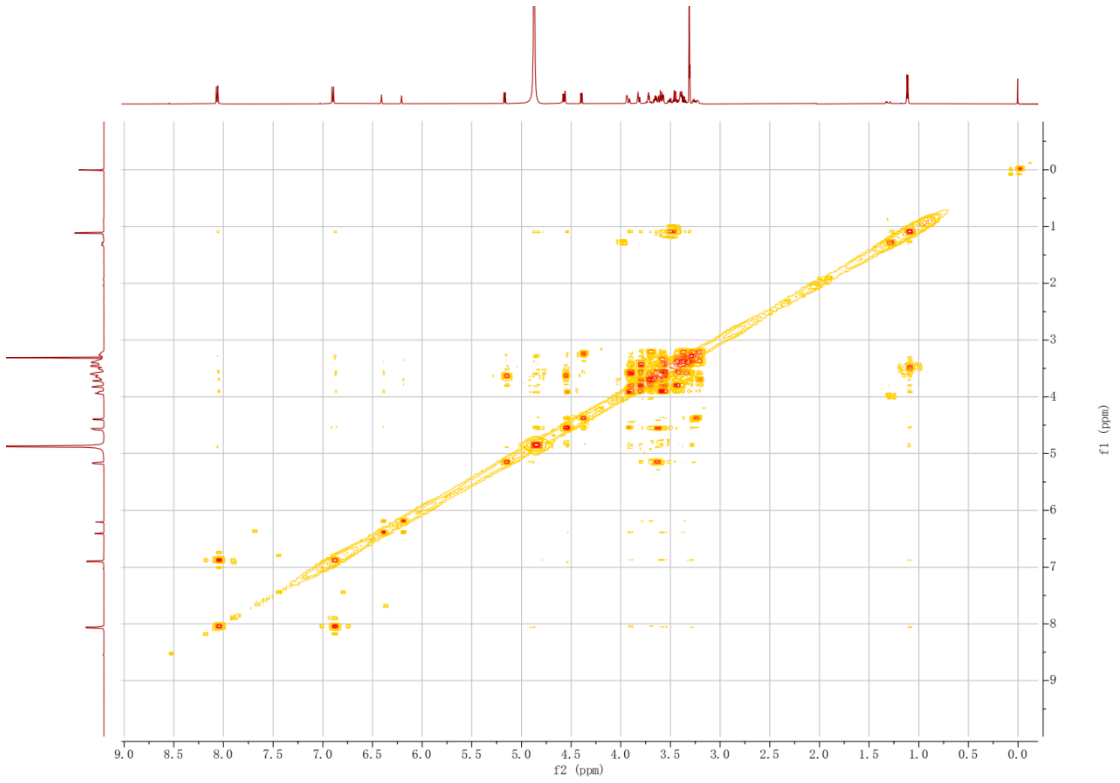


**Figure S43.** COSY data of compound **34** in methanol-$d_4$.

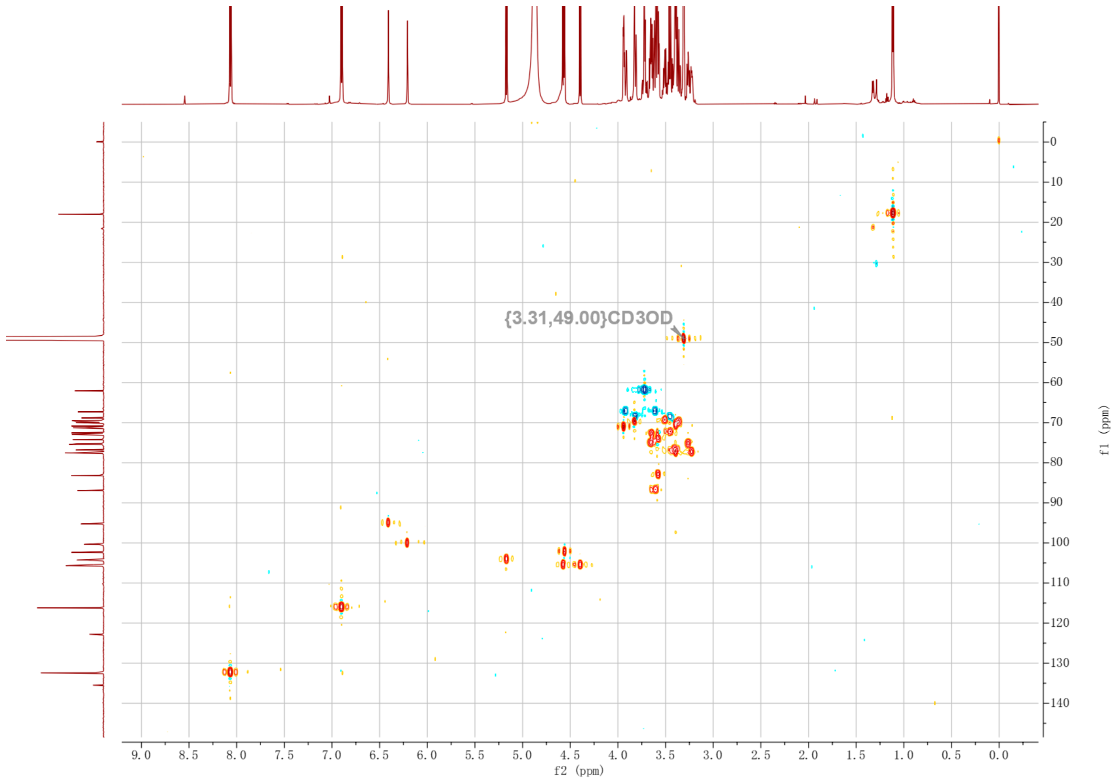


**Figure S44.** HSQC data of compound **34** in methanol-$d_4$.

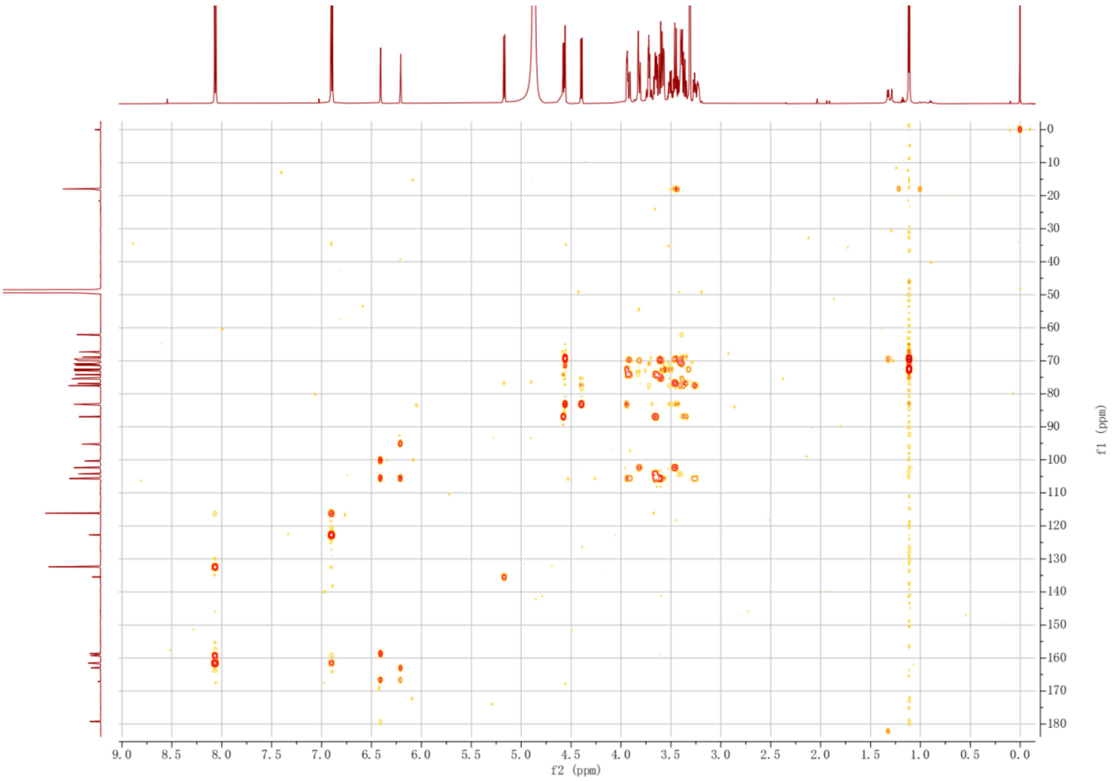


**Figure S45.** HMBC data of compound **34** in methanol-$d_4$.

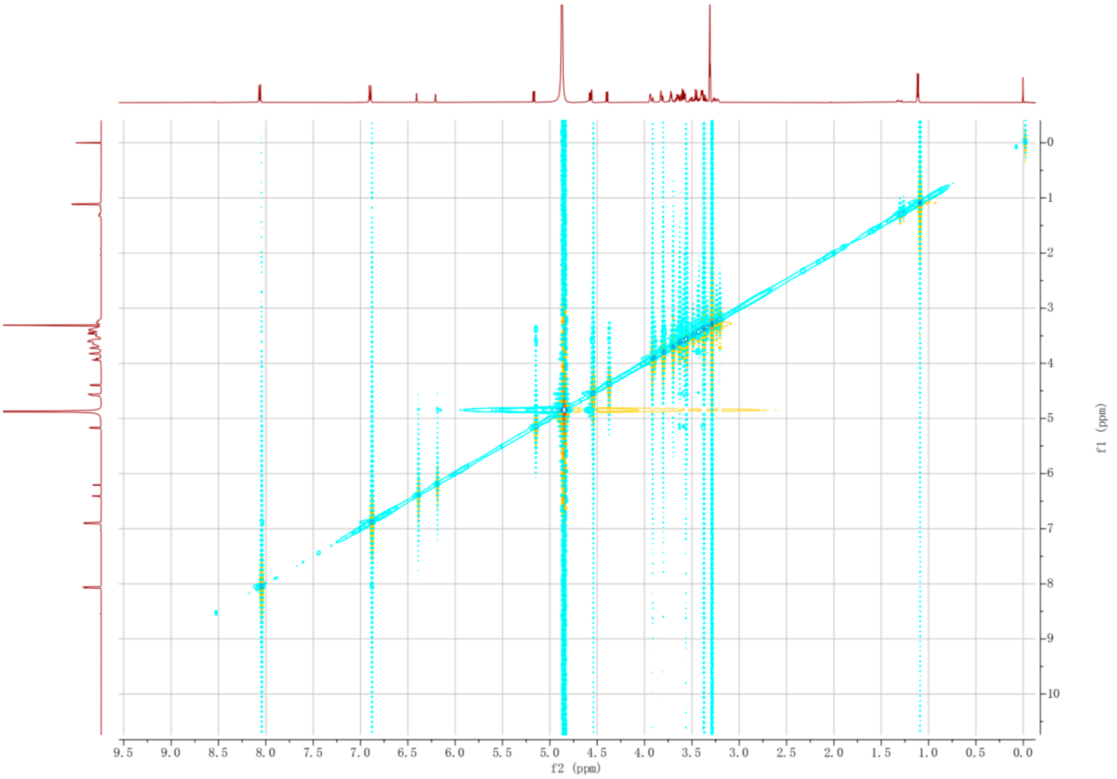


**Figure S46.** NOESY data of compound **34** in methanol-$d_4$.

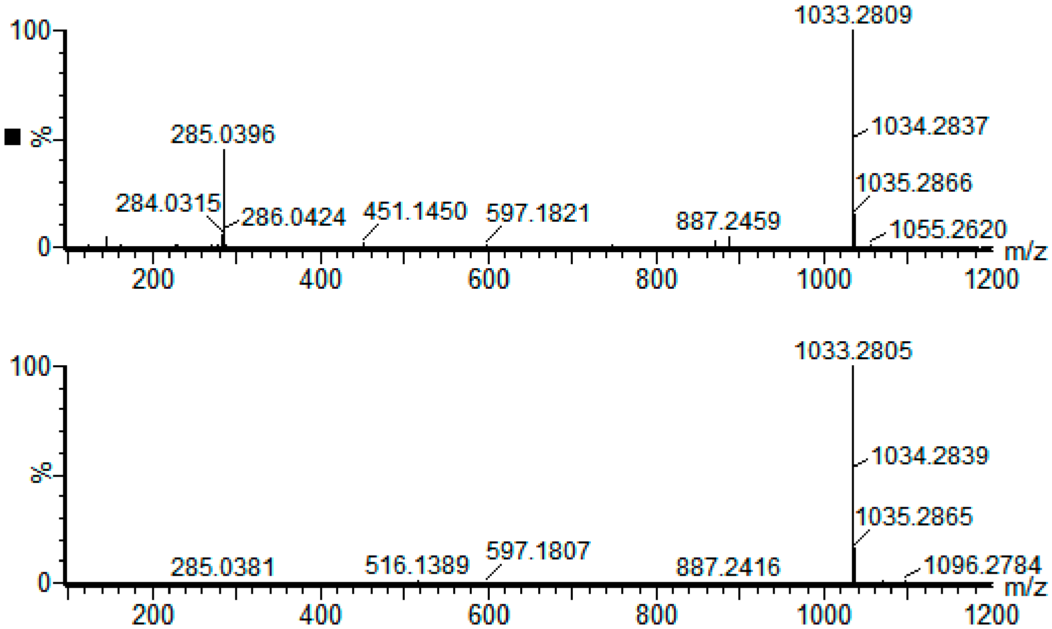


**Figure S47.** HR-ESI-MS data of compound **36** in the negative mode.

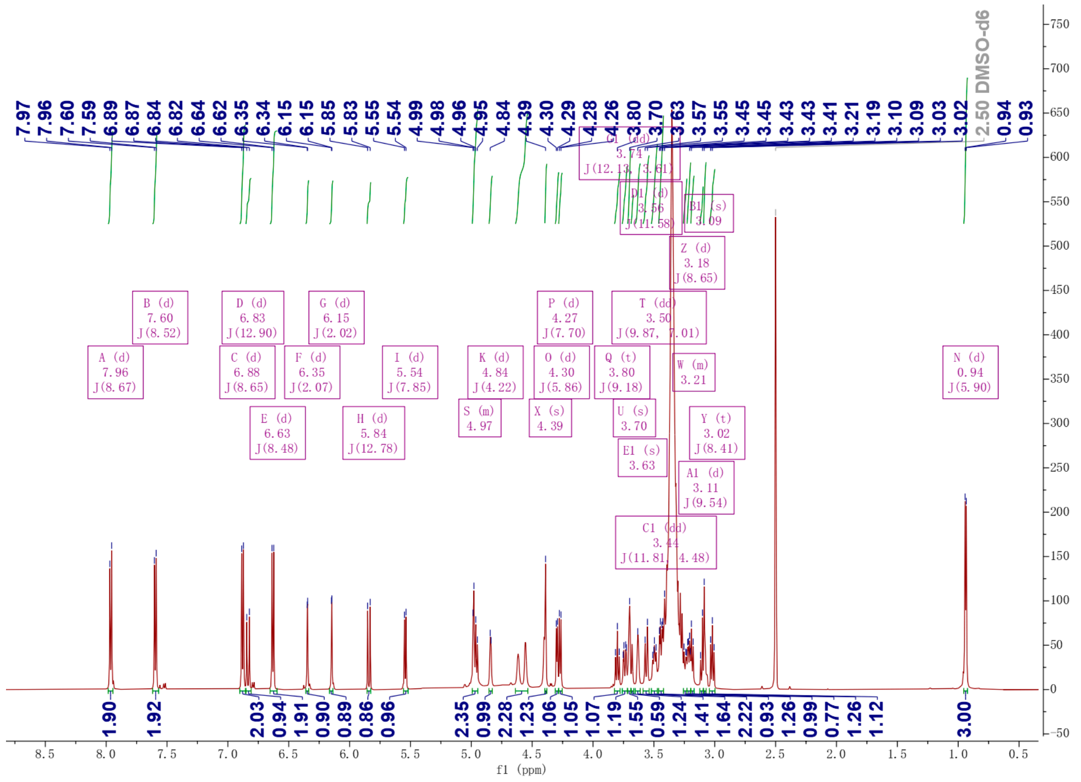


**Figure S48.** $^{1}$H NMR data of compound **36** in DMSO-$d_6$.

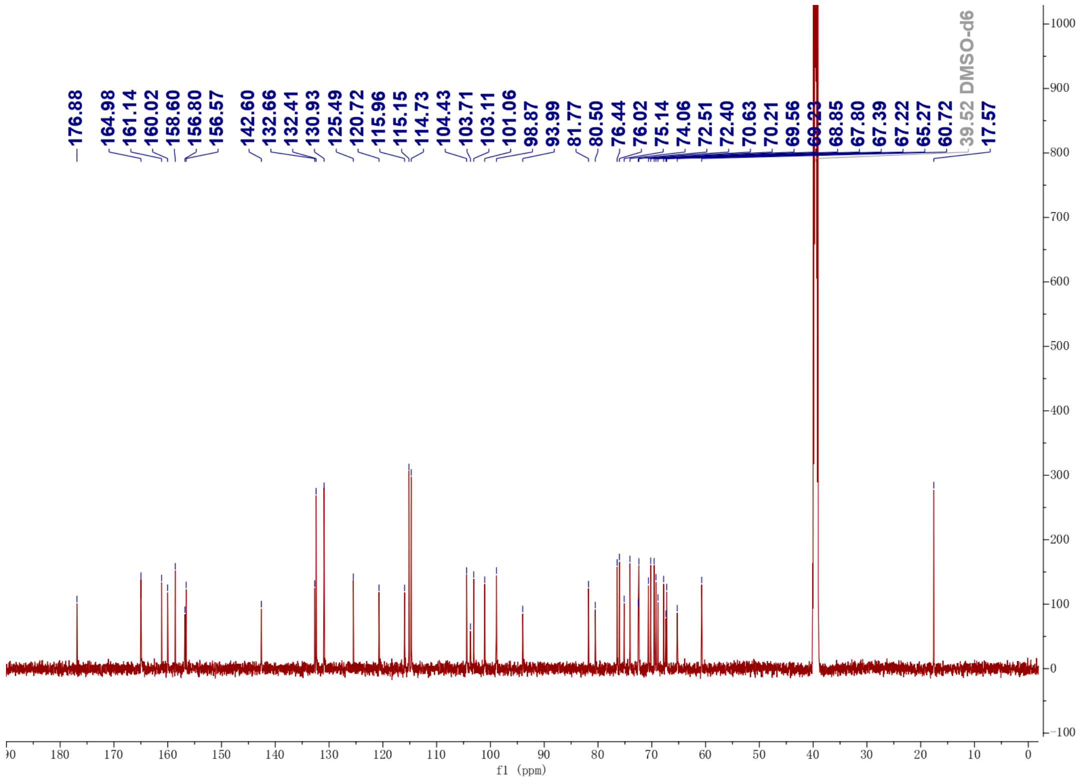

**Figure S49.** $^{13}C$ NMR data of compound **36** in DMSO-$d_6$.

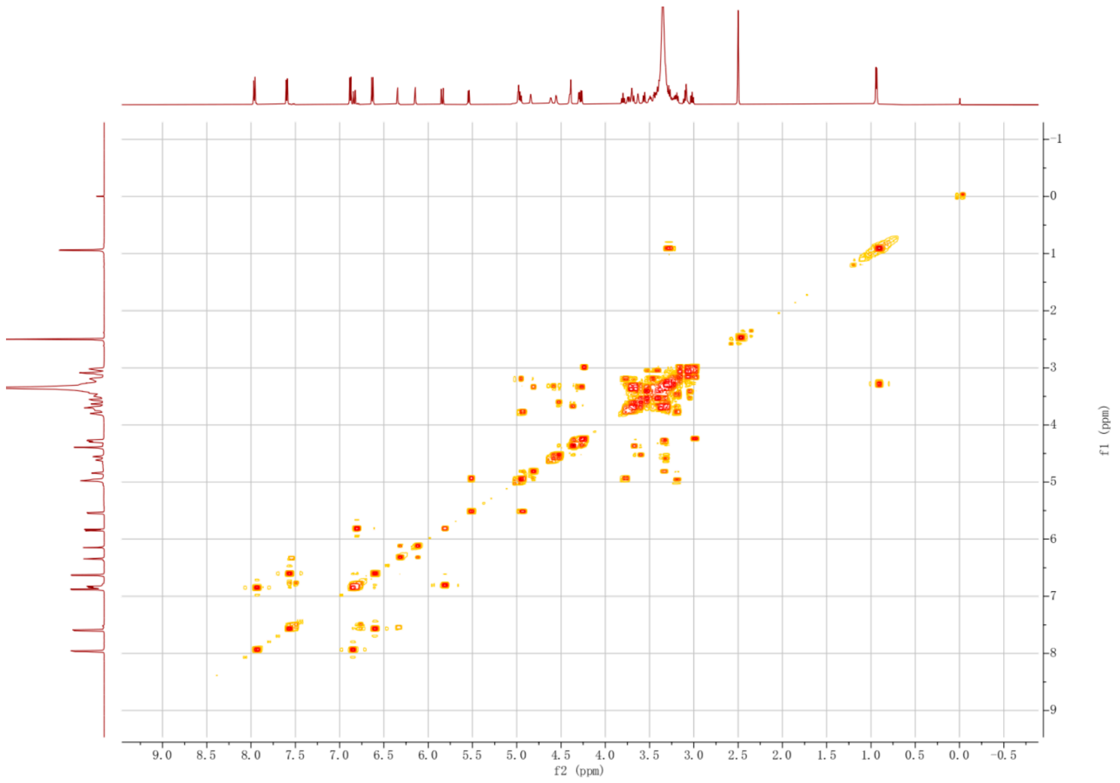

**Figure S50.** COSY data of compound **36** in DMSO-$d_6$.

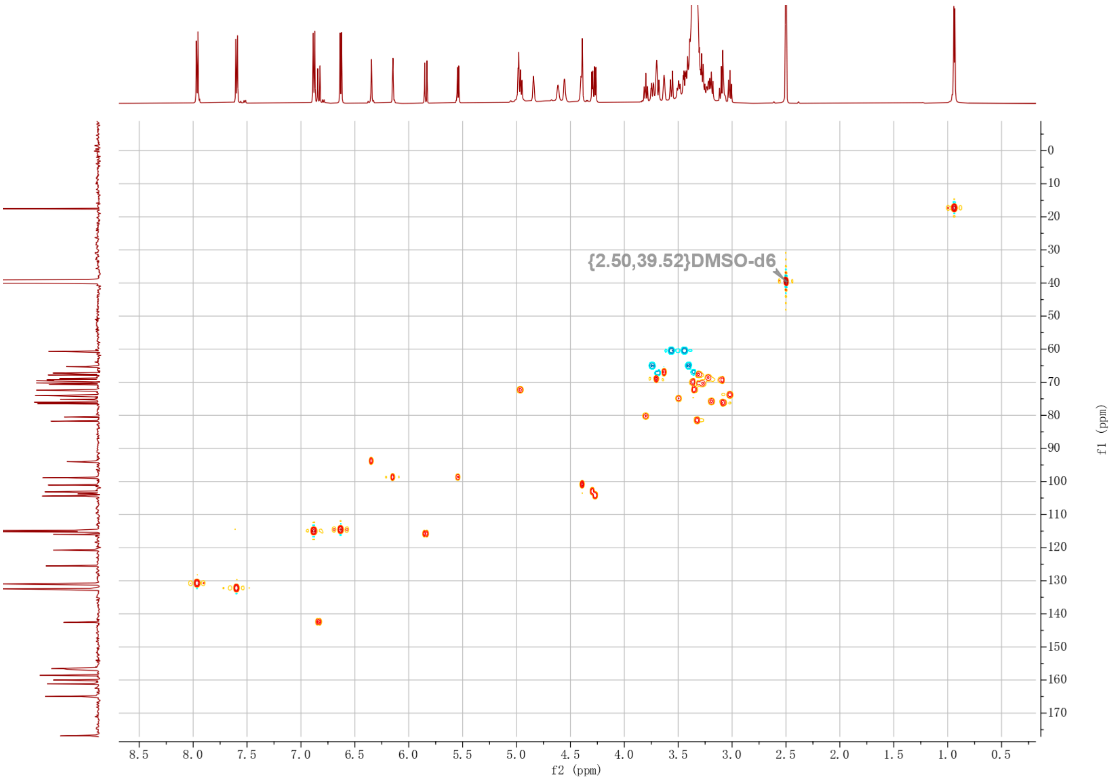


**Figure S51.** HSQC data of compound **36** in DMSO-*d*6.

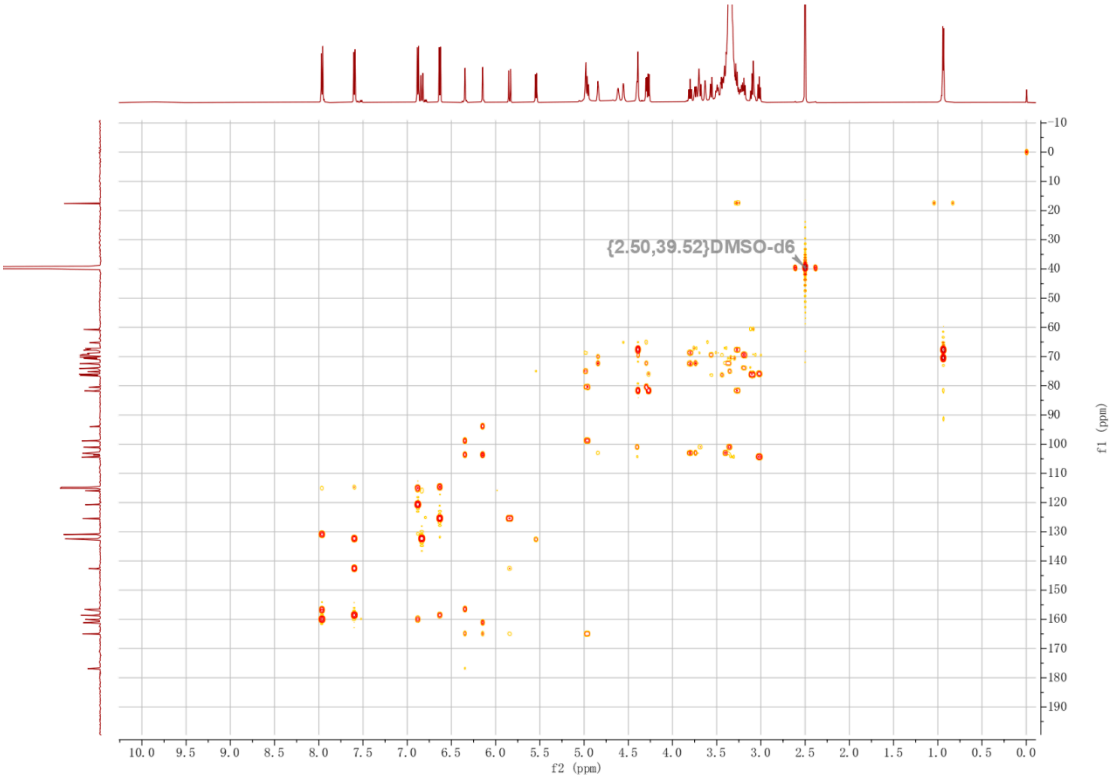


**Figure S52.** HMBC data of compound **36** in DMSO-*d*6.

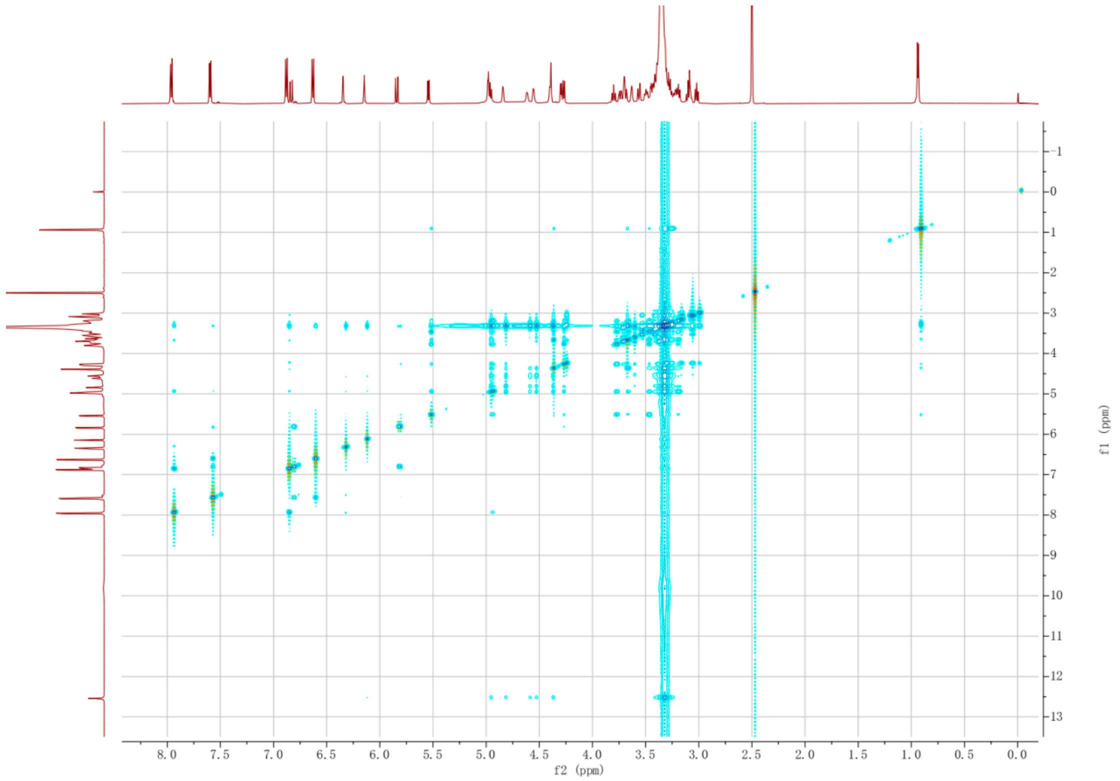


**Figure S53.** NOESY data of compound **36** in DMSO-$d_6$.

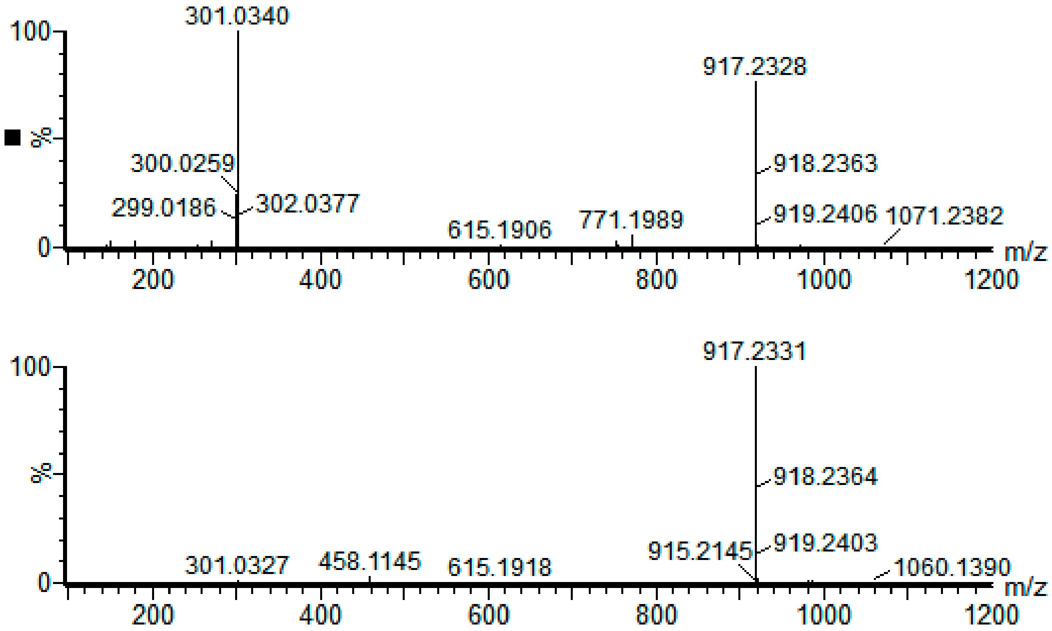


**Figure S54.** HR-ESI-MS data of compound **43** in the negative mode.

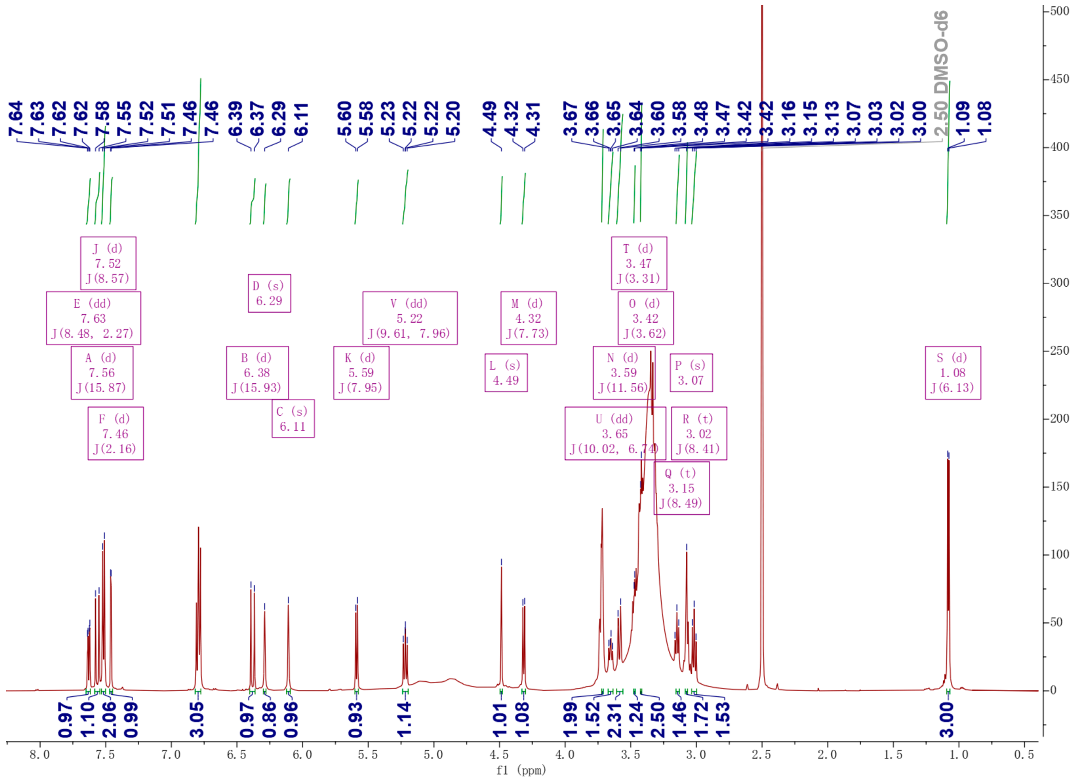


**Figure S55.** $^{1}$H NMR data of compound **43** in DMSO-$d_6$.

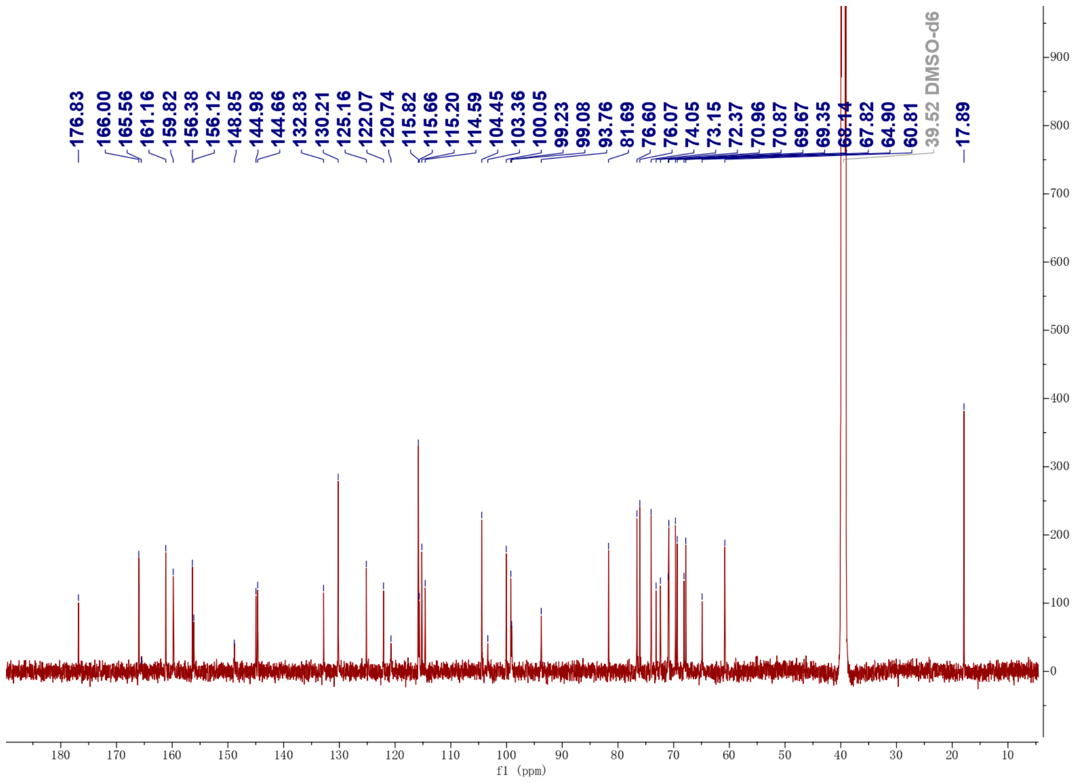


**Figure S56.** $^{13}$C NMR data of compound **43** in DMSO-$d_6$.

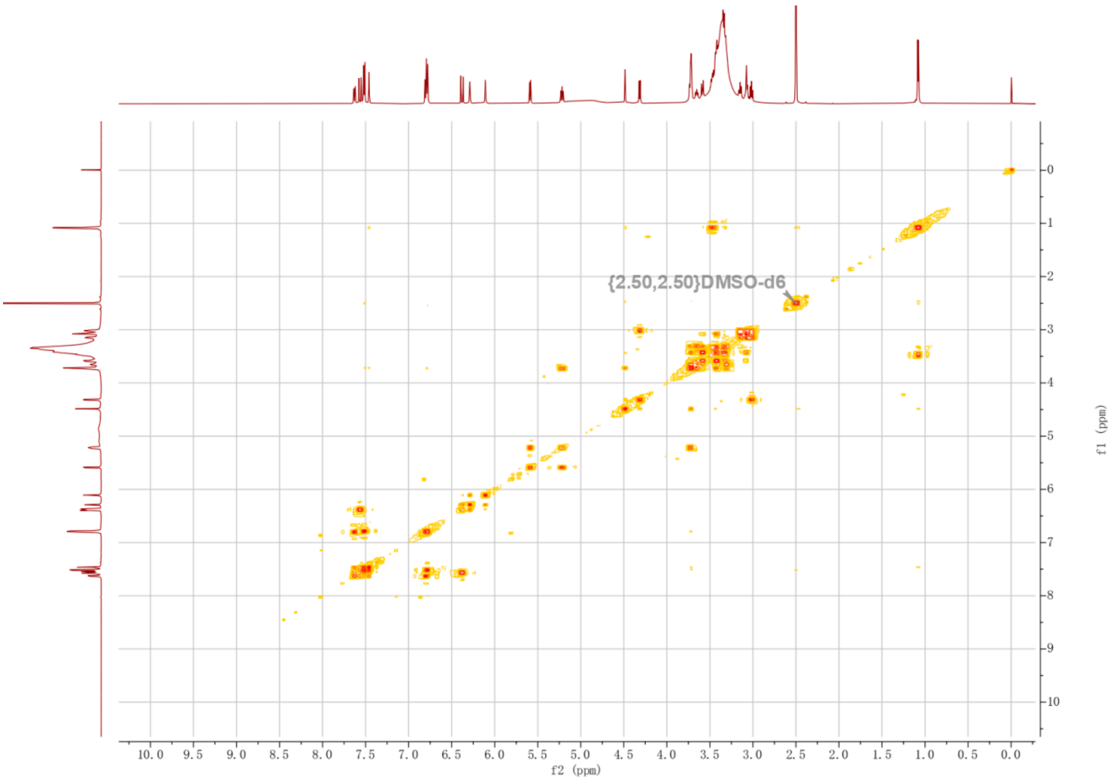


**Figure S57.** COSY data of compound **43** in DMSO-$d_6$.

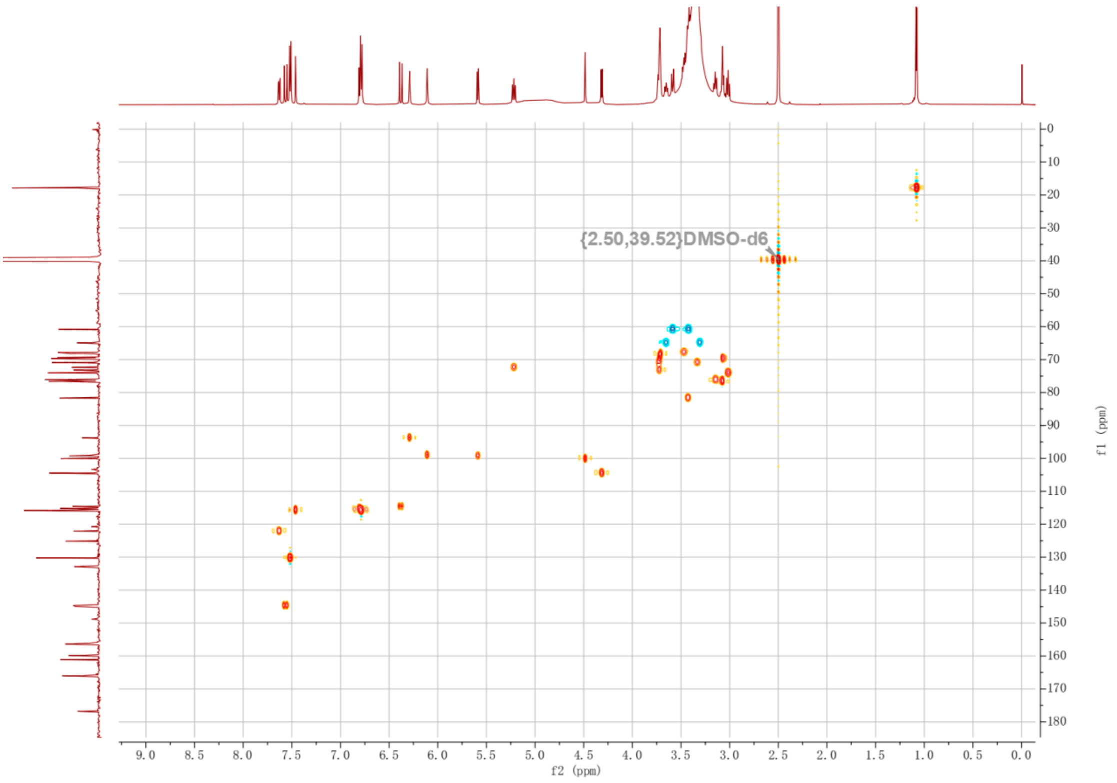


**Figure S58.** HSQC data of compound **43** in DMSO-$d_6$.

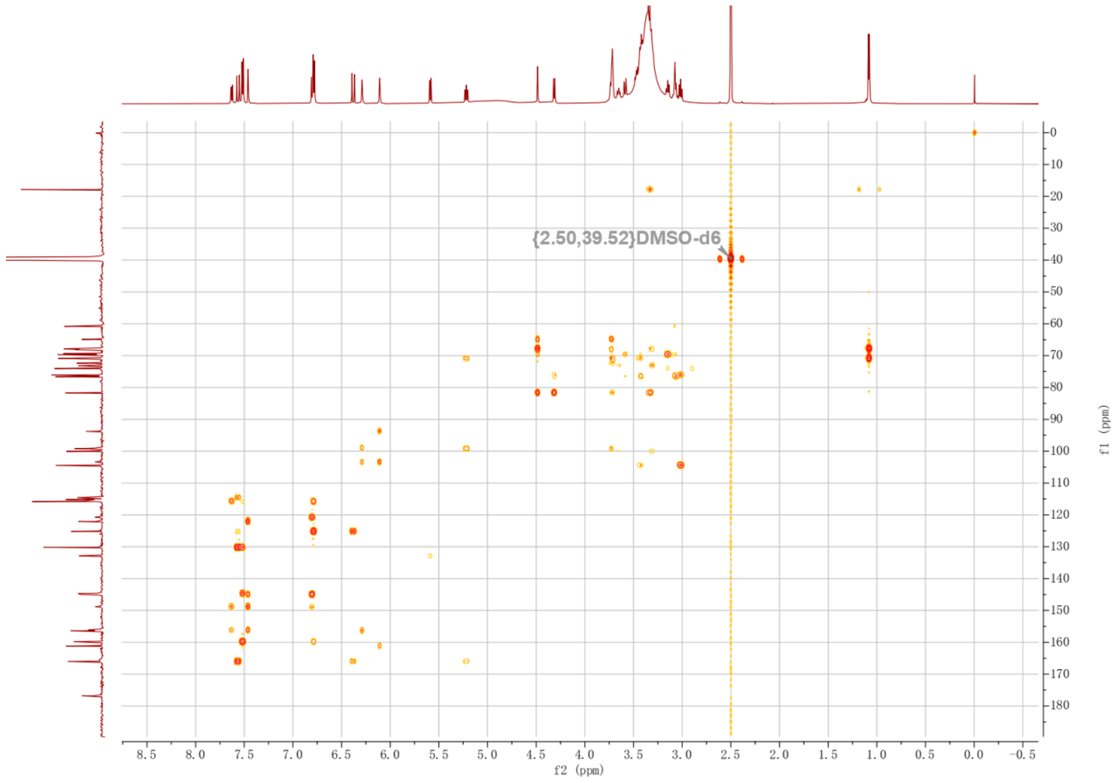


**Figure S59.** HMBC data of compound **43** in DMSO-*d*6.

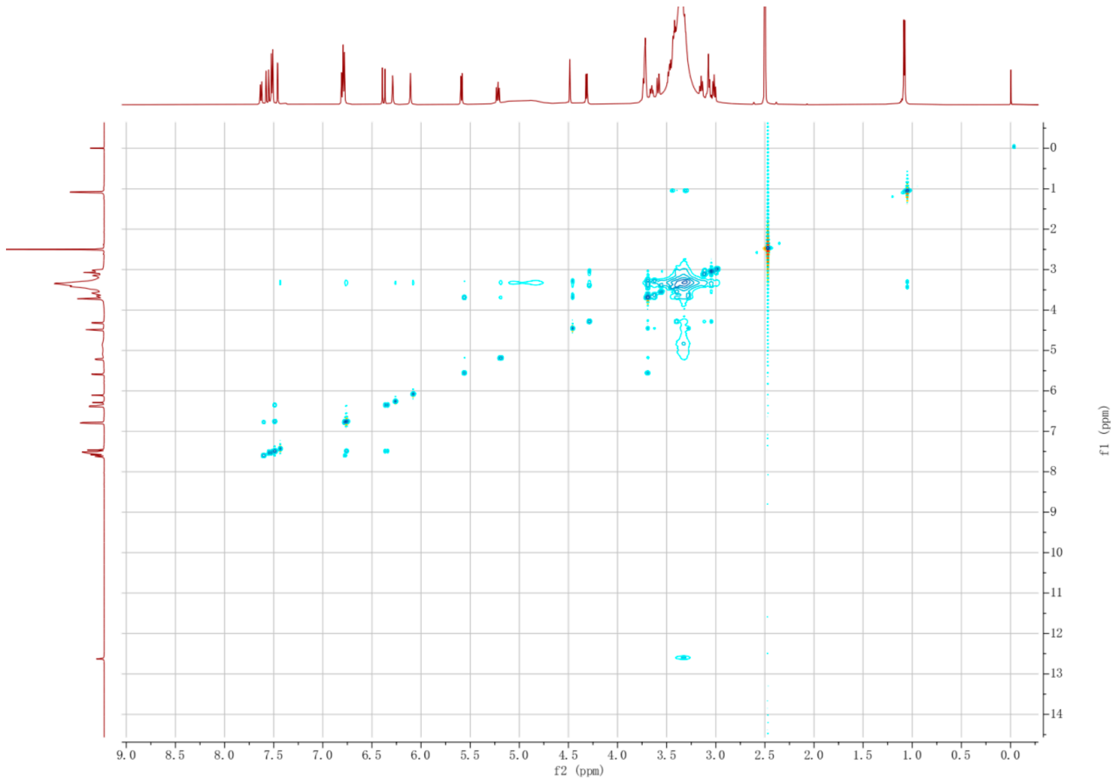


**Figure S60.** NOESY data of compound **43** in DMSO-*d*6.

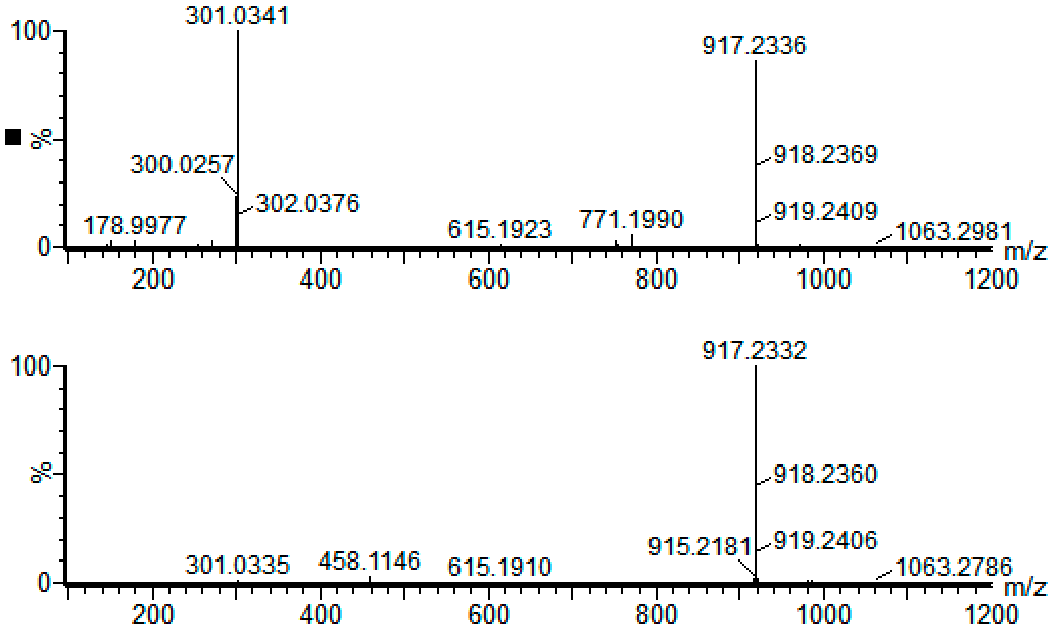


**Figure S61.** HR-ESI-MS data of compound **44** in the negative mode.

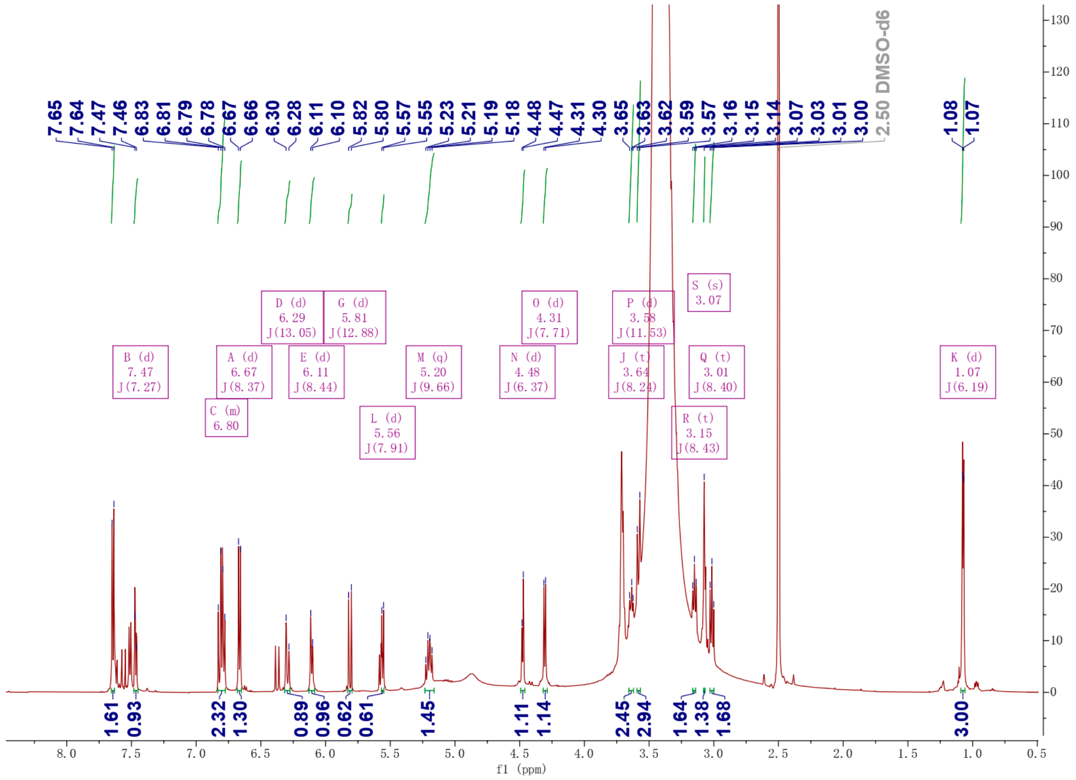


**Figure S62.** $^{1}$H NMR data of compound **44** in DMSO-$d_6$.

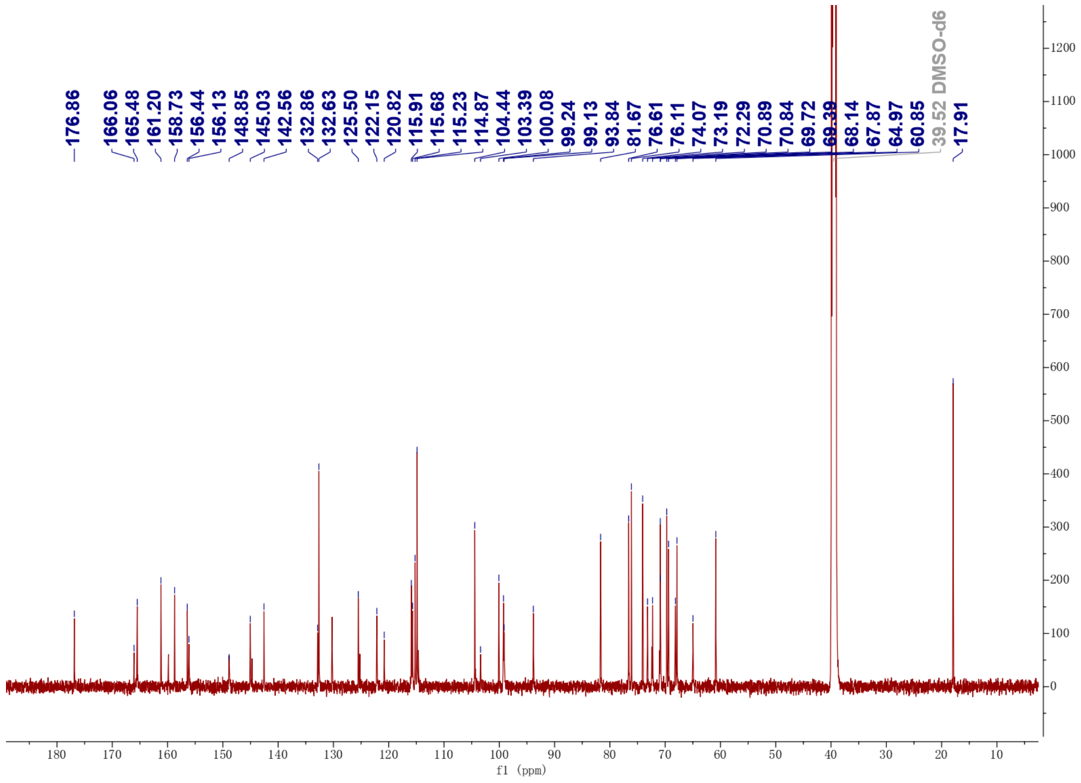


**Figure S63.** $^{13}$C NMR data of compound **44** in DMSO-$d_6$.

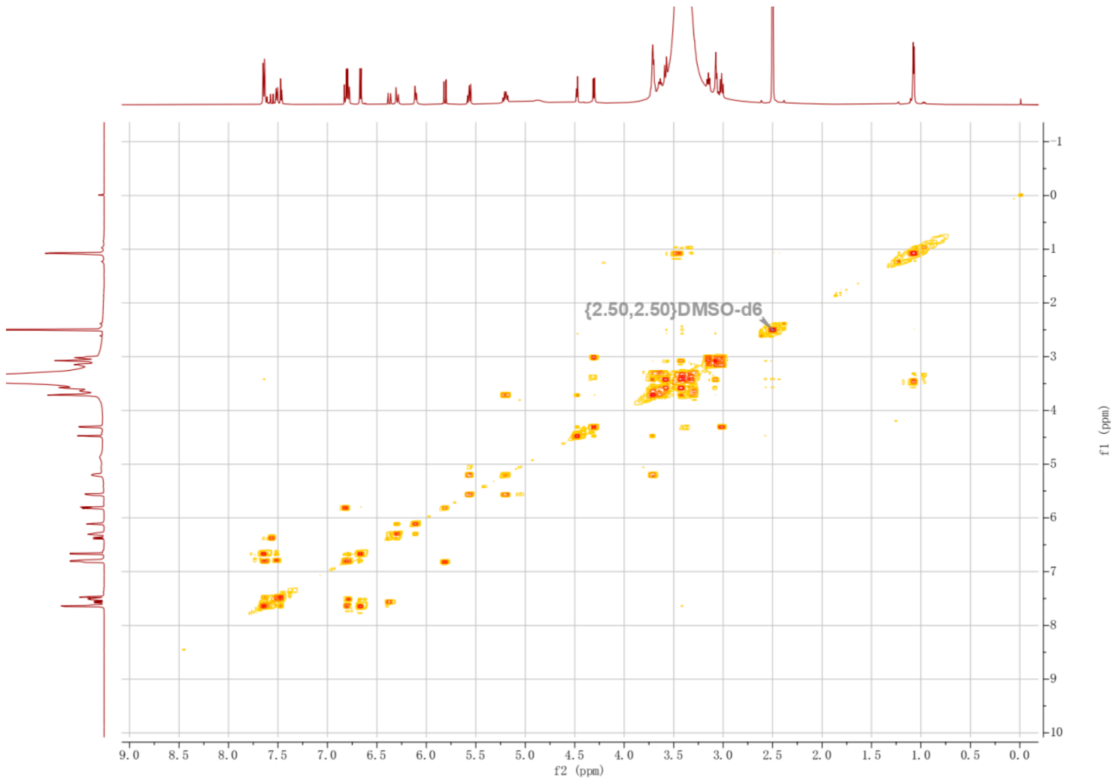


**Figure S64.** COSY data of compound **44** in DMSO-$d_6$.

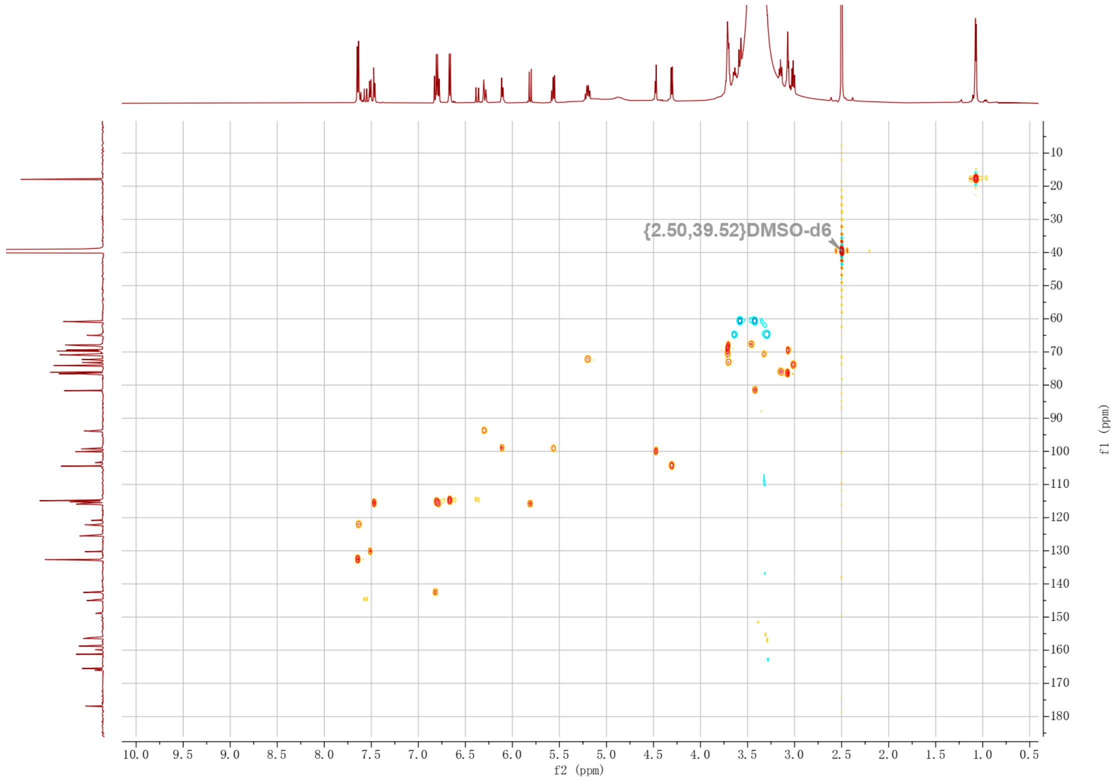


**Figure S65.** HSQC data of compound **44** in DMSO-$d_6$.

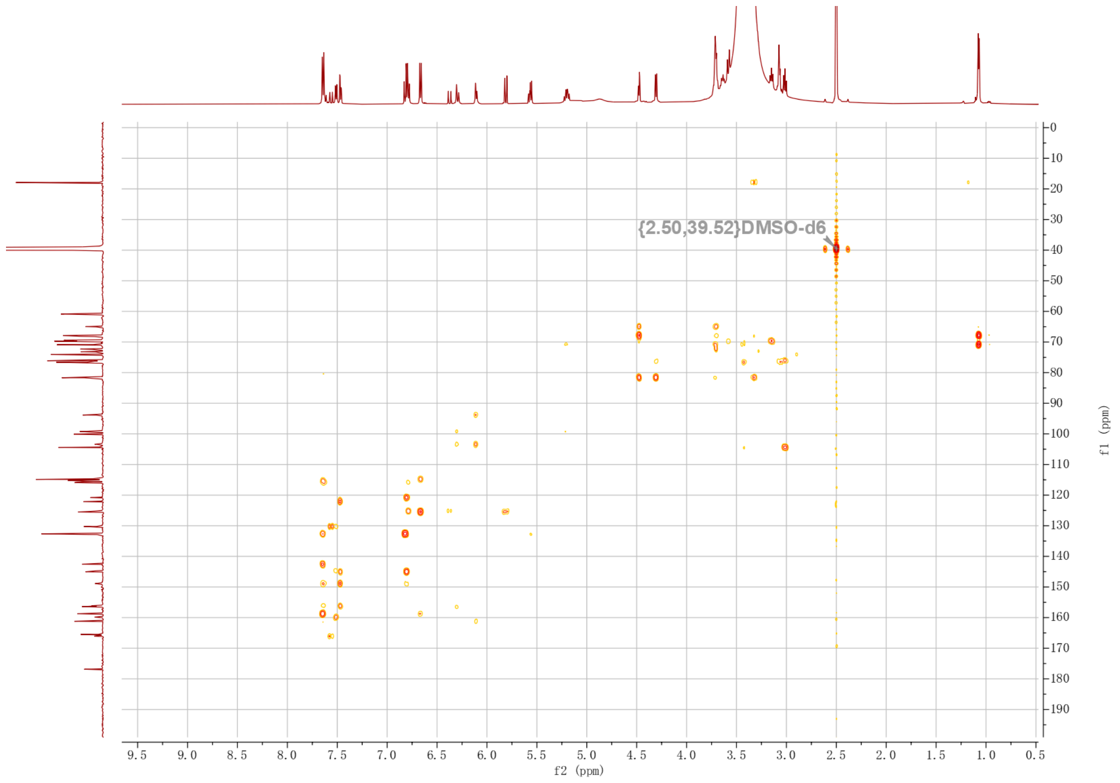


**Figure S66.** HMBC data of compound **44** in DMSO-$d_6$.

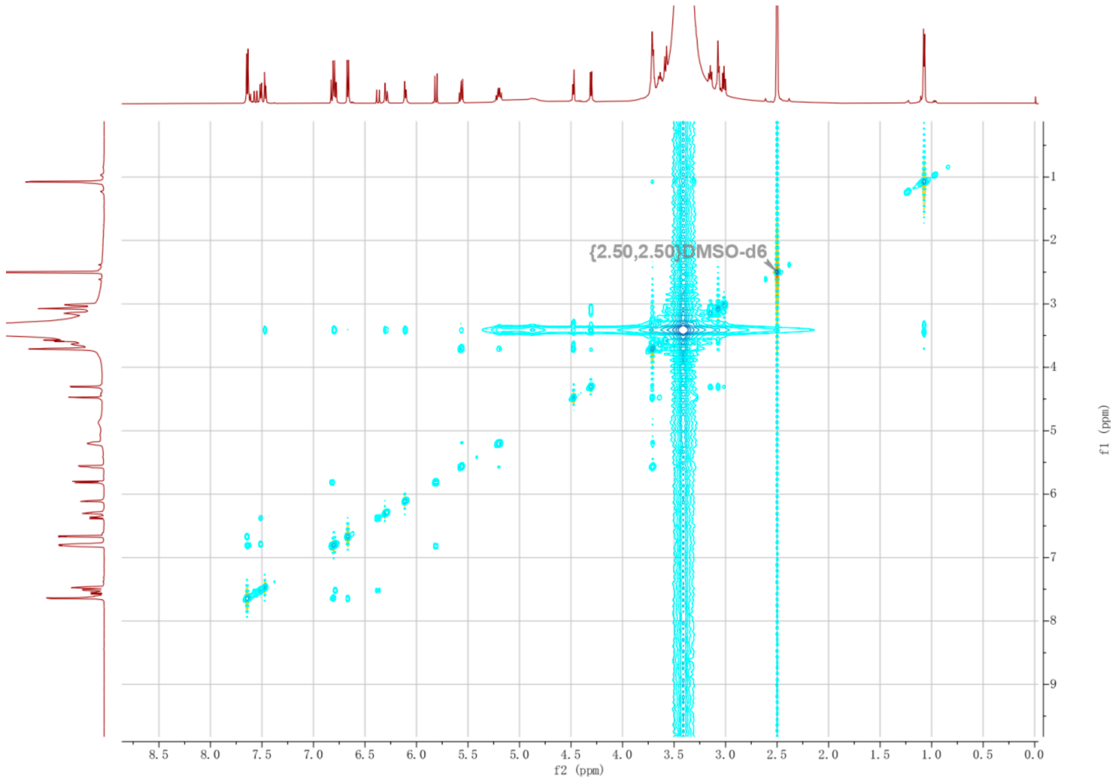


**Figure S67.** NOESY data of compound **44** in DMSO-*d*6.

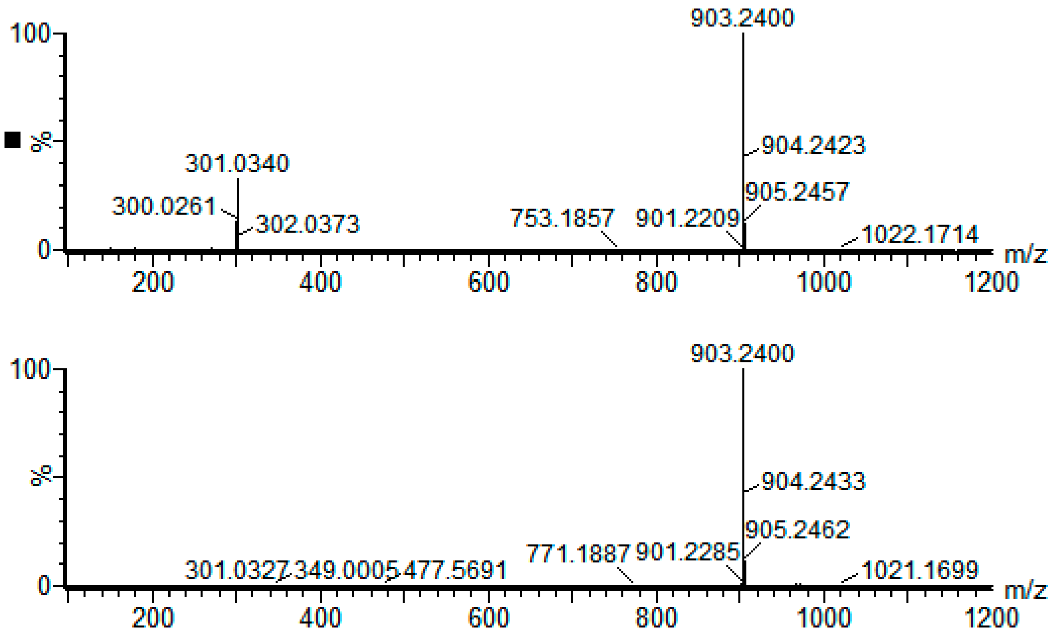


**Figure S68.** HR-ESI-MS data of compound **46** in the negative mode.

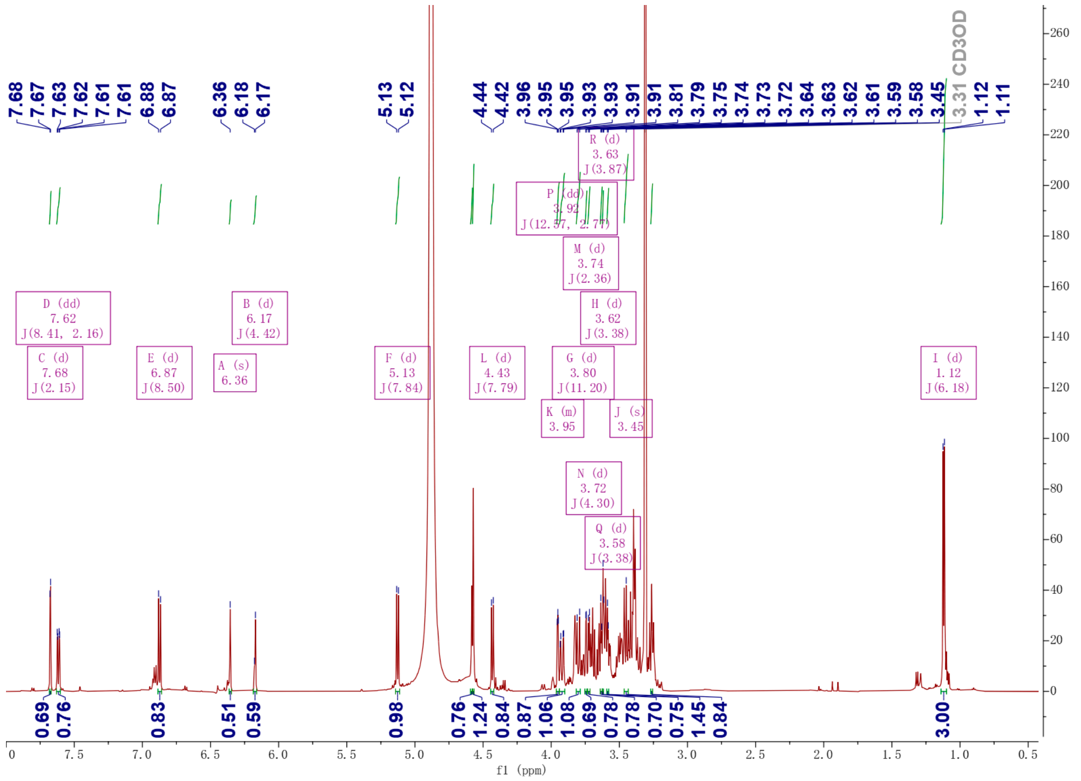


**Figure S69.** $^{1}$H NMR data of compound **46** in methanol-$d_4$.

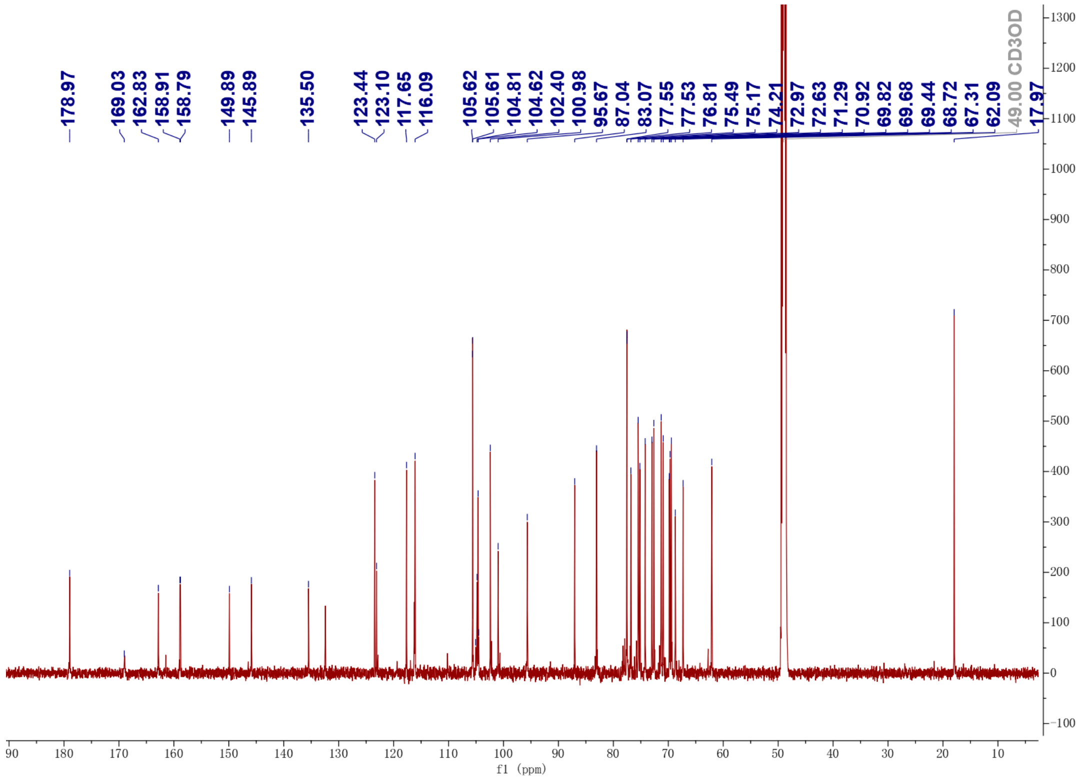


**Figure S70.** $^{13}$C NMR data of compound **46** in methanol-$d_4$.

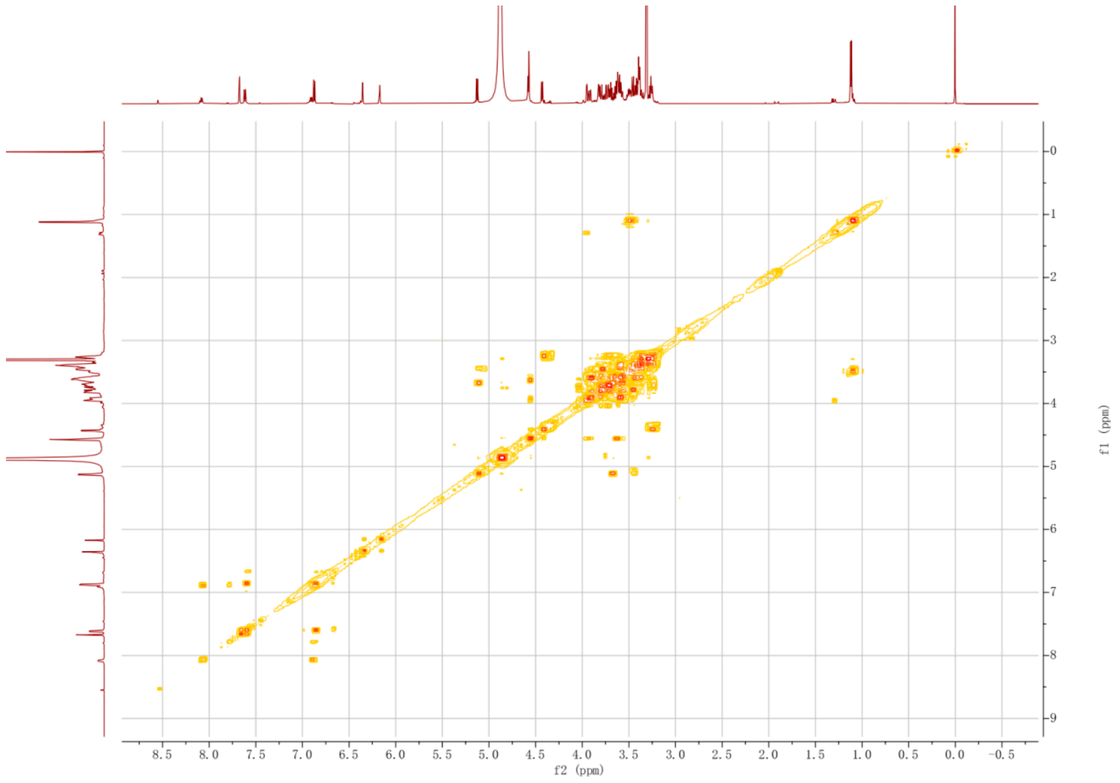


**Figure S71.** COSY data of compound **46** in methanol-$d_4$.

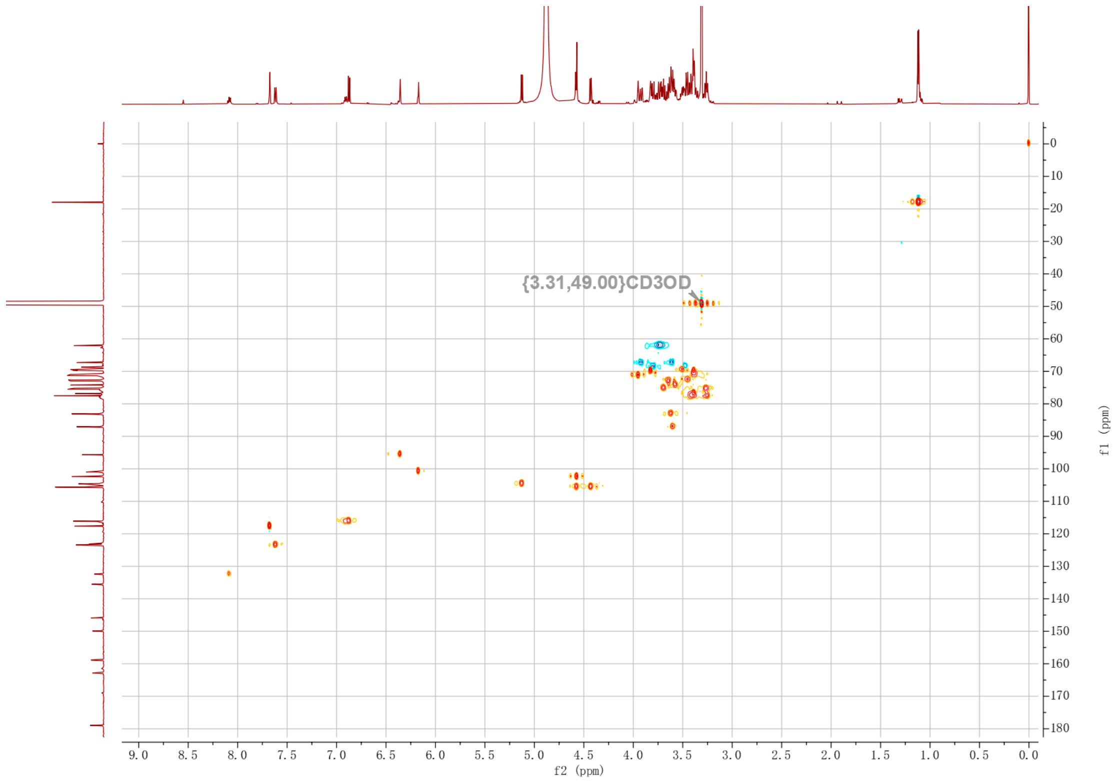


**Figure S72.** HSQC data of compound **46** in methanol-$d_4$.

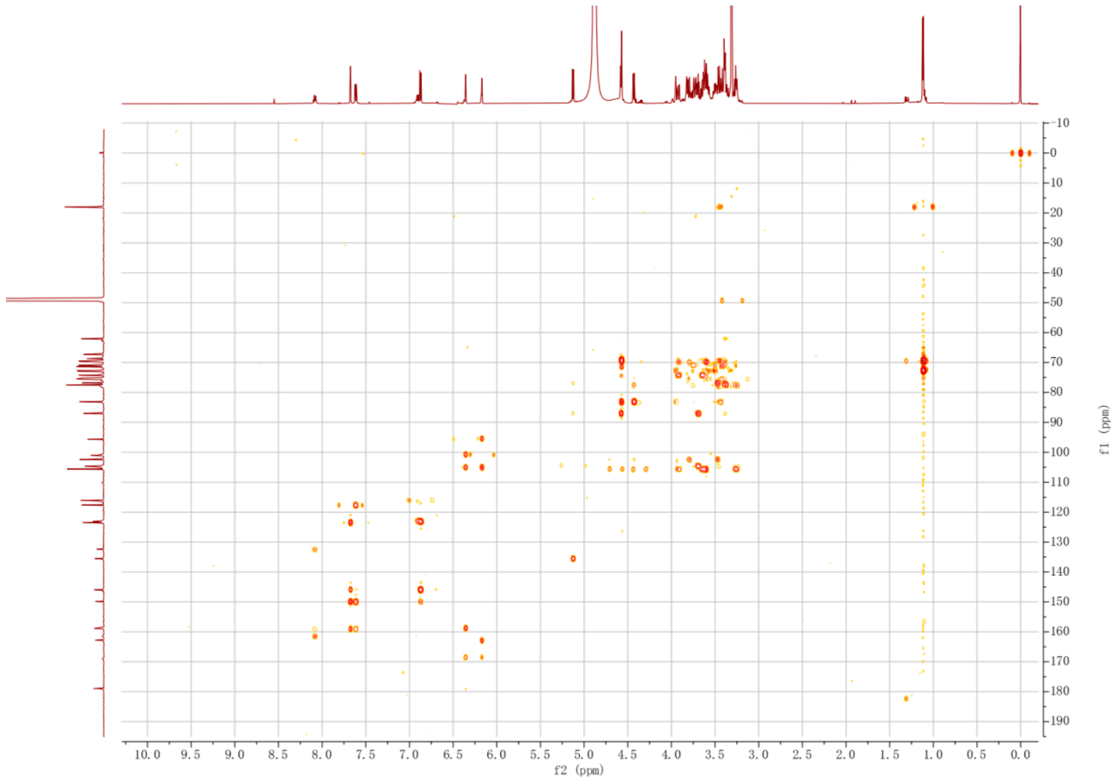


**Figure S73.** HMBC data of compound **46** in methanol-*d*4.

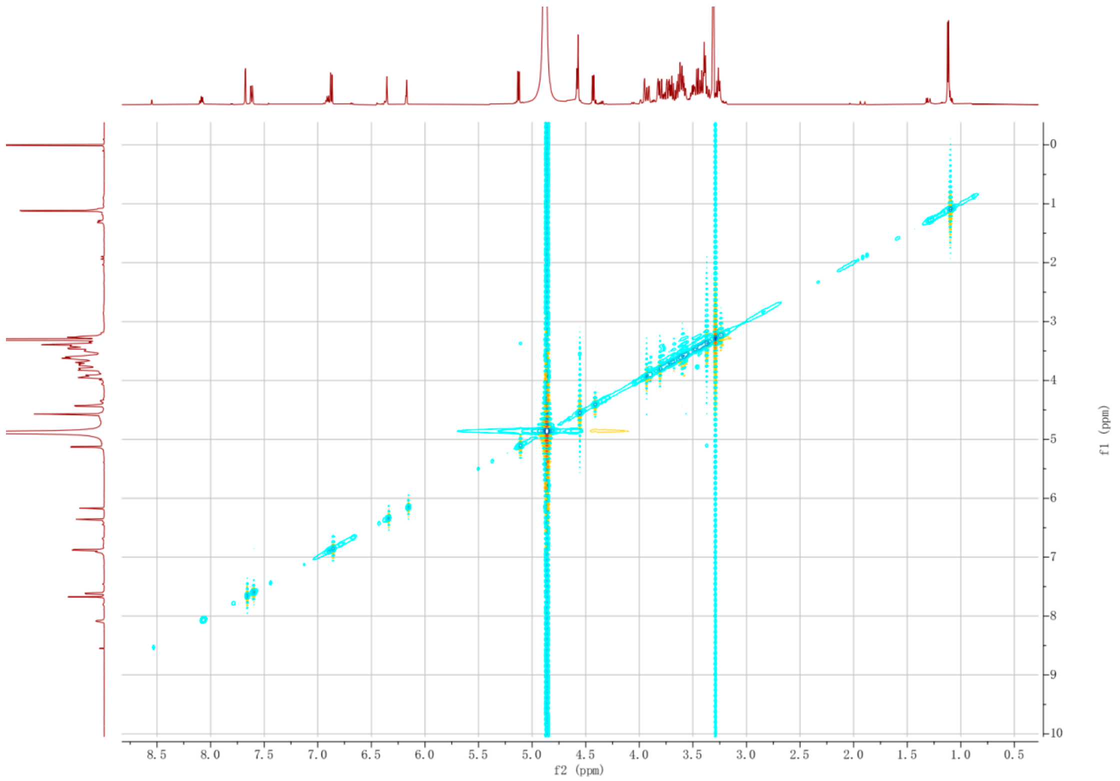


**Figure S74.** NOESY data of compound **46** in methanol-*d*4.

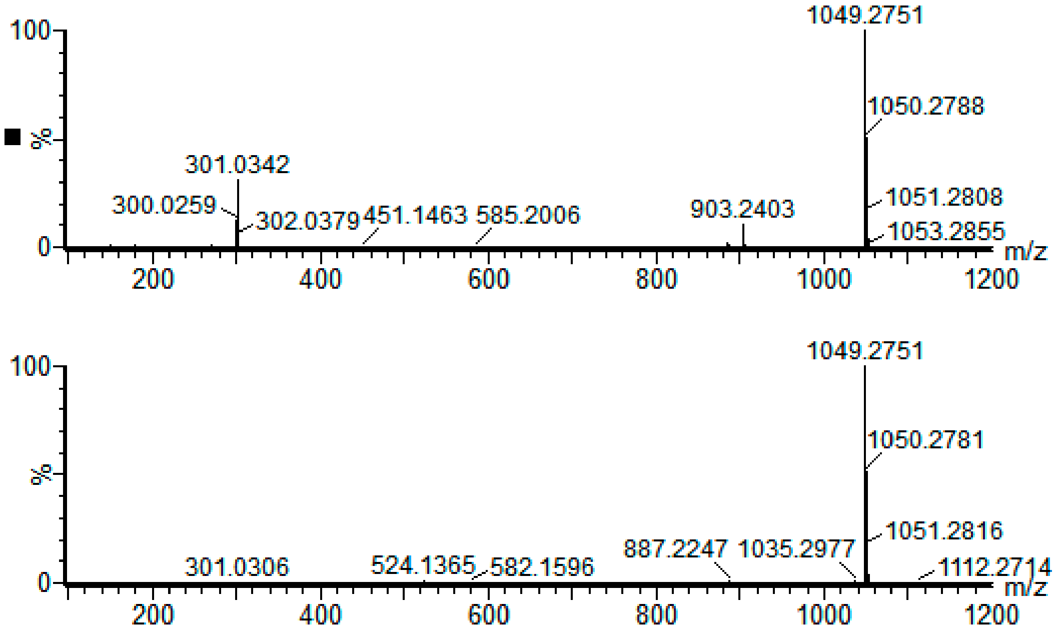


**Figure S75.** HR-ESI-MS data of compound **48** in the negative mode.

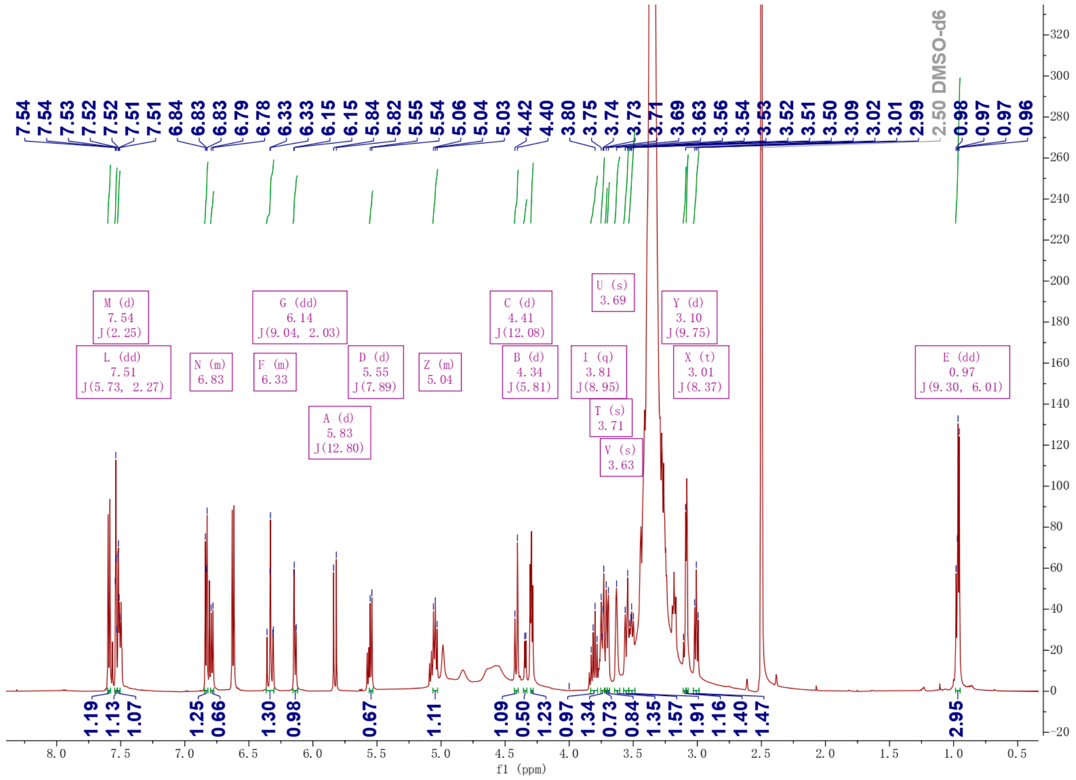


**Figure S76.** $^{1}$H NMR data of compound **48** in DMSO-$d_6$.

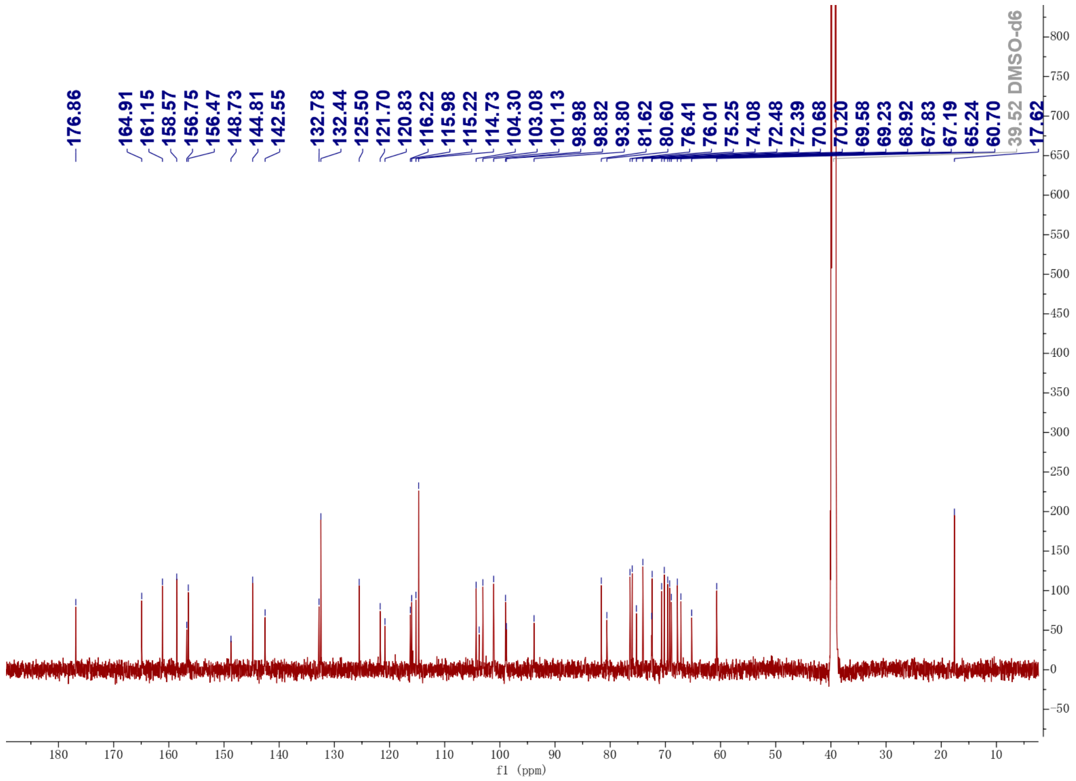


**Figure S77.** $^{13}C$ NMR data of compound **48** in DMSO-$d_6$.

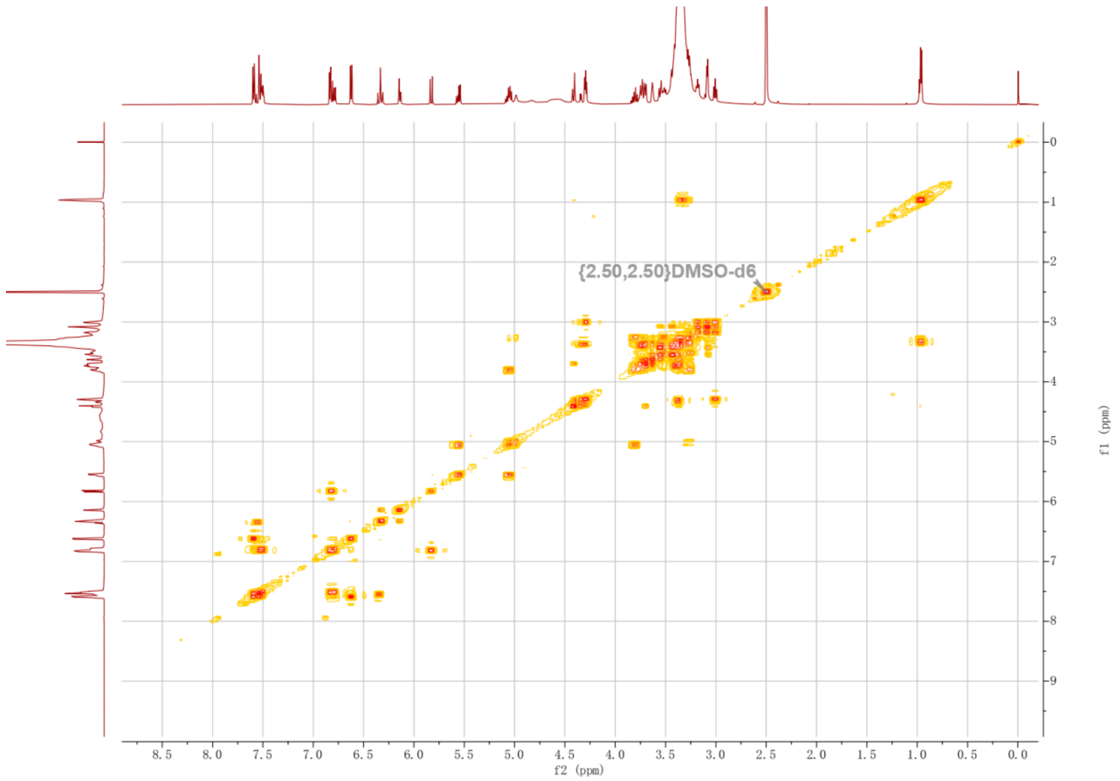


**Figure S78.** COSY data of compound **48** in DMSO-$d_6$.

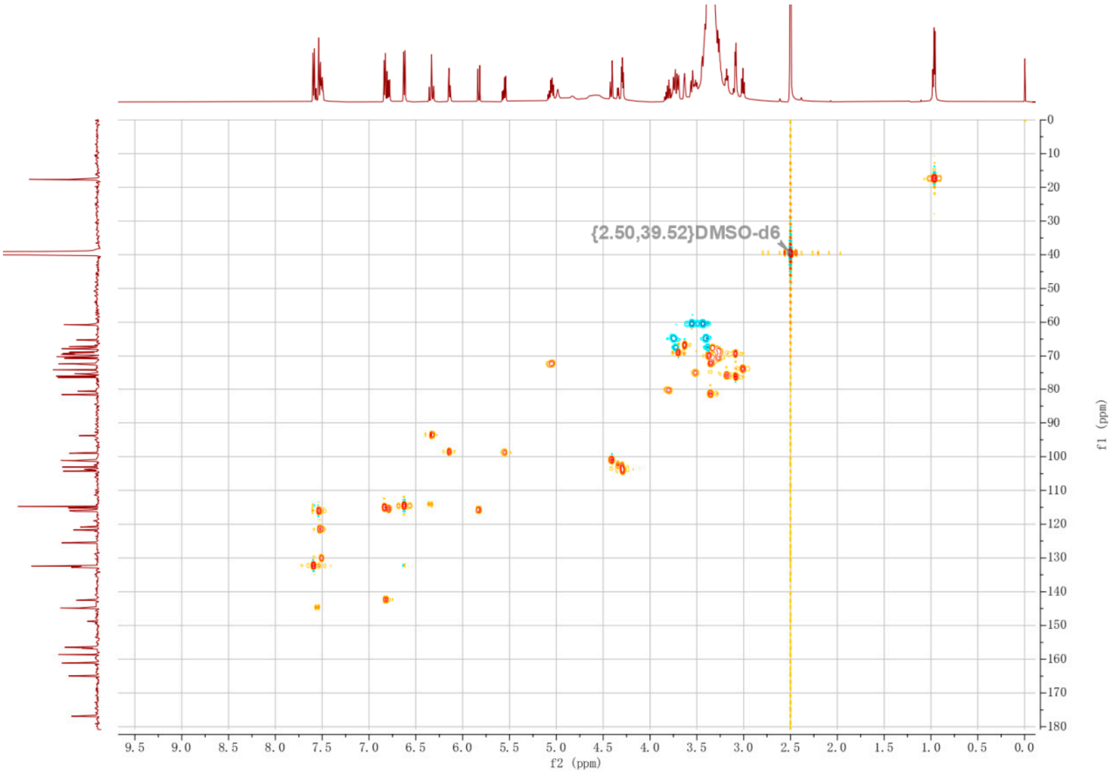


**Figure S79.** HSQC data of compound **48** in DMSO-$d_6$.

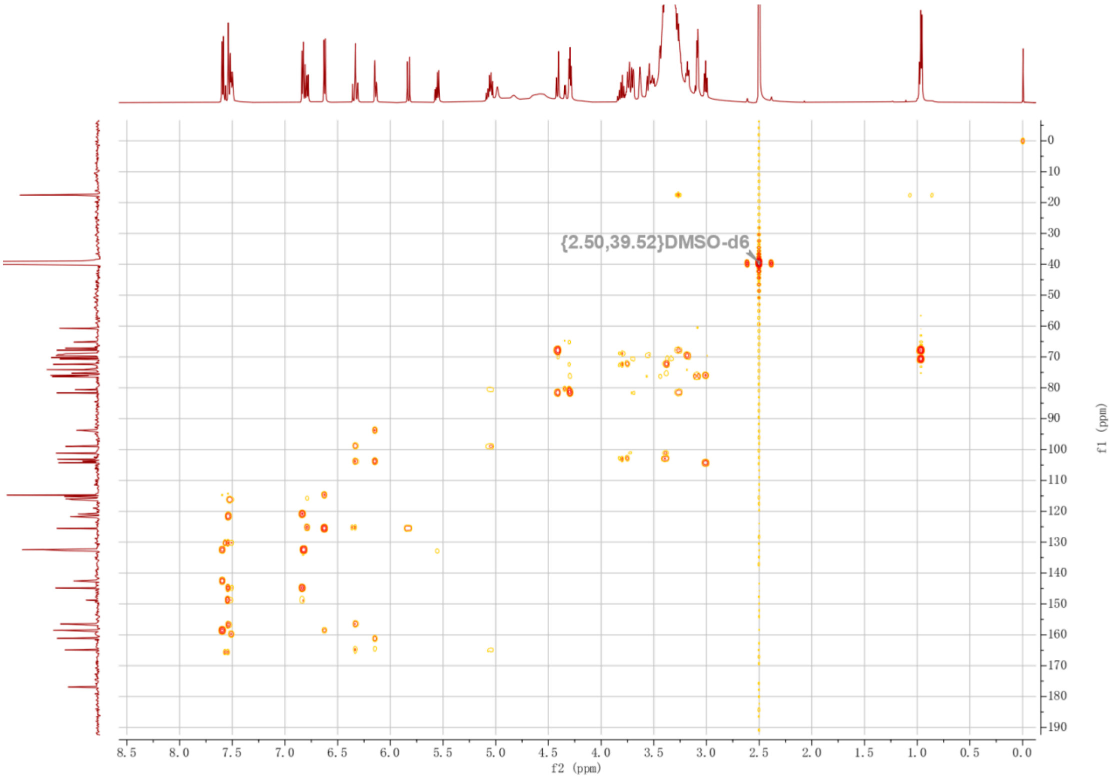


**Figure S80.** HMBC data of compound **48** in DMSO-$d_6$.

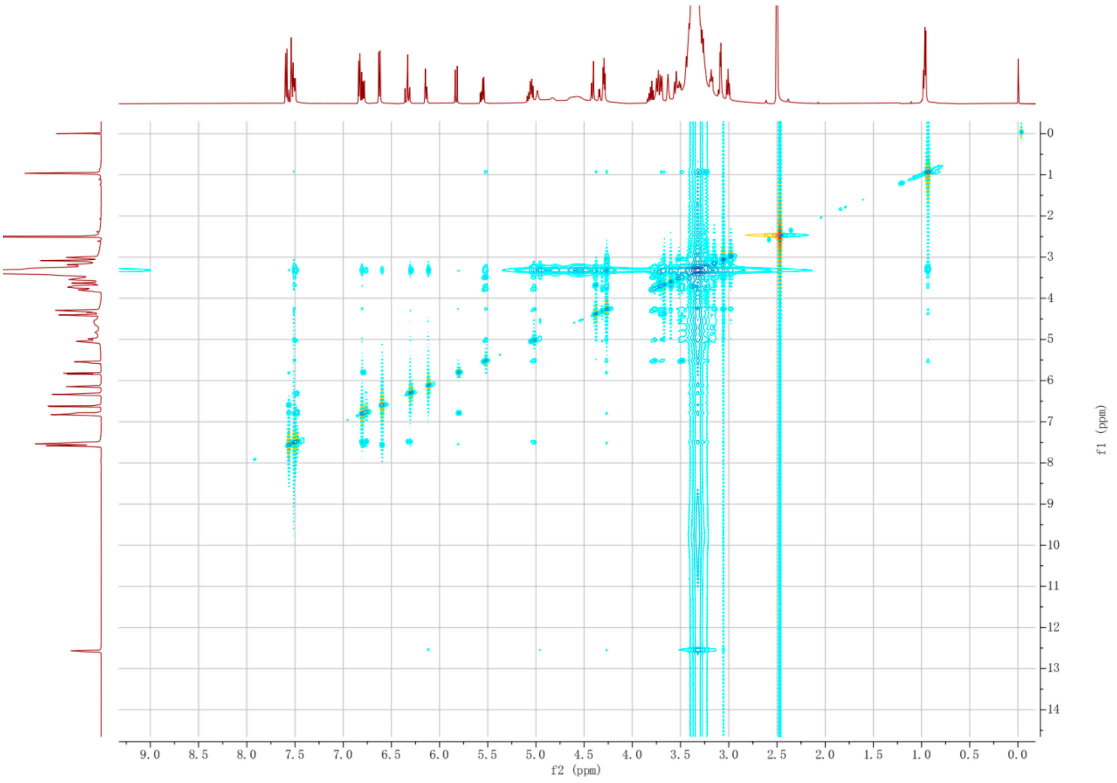


**Figure S81.** NOESY data of compound **48** in DMSO-$d_6$.

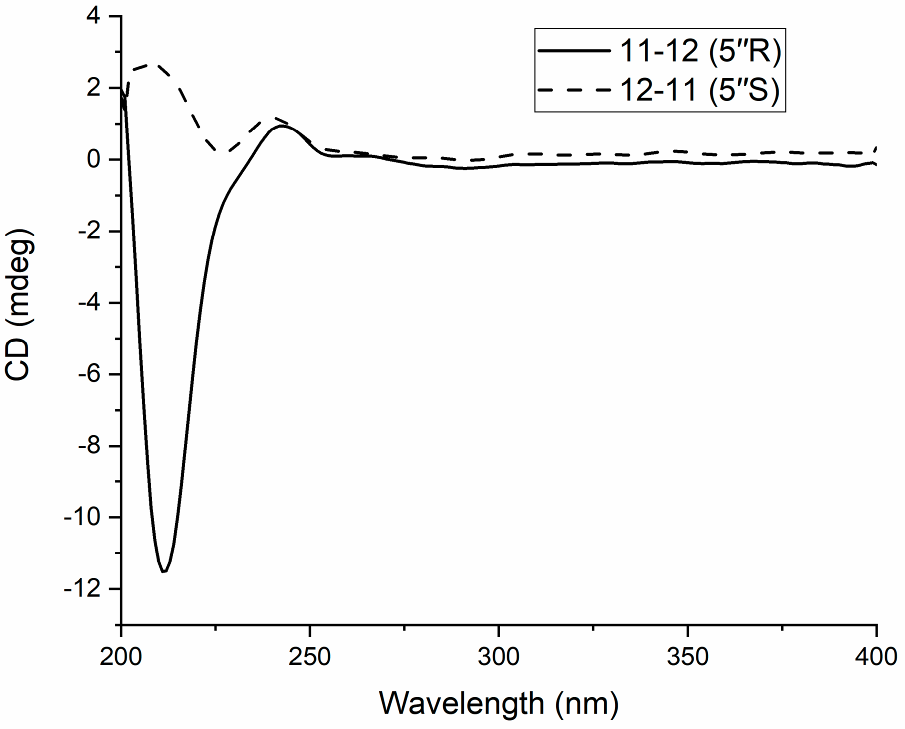


**Figure S82.** Circular dichroism (CD) spectra of **11** and **12**.
